# New DArT markers for oat provide enhanced map coverage and global germplasm characterization

**DOI:** 10.1186/1471-2164-10-39

**Published:** 2009-01-21

**Authors:** Nicholas A Tinker, Andrzej Kilian, Charlene P Wight, Katarzyna Heller-Uszynska, Peter Wenzl, Howard W Rines, Åsmund Bjørnstad, Catherine J Howarth, Jean-Luc Jannink, Joseph M Anderson, Brian G Rossnagel, Deon D Stuthman, Mark E Sorrells, Eric W Jackson, Stine Tuvesson, Frederic L Kolb, Olof Olsson, Luiz Carlos Federizzi, Marty L Carson, Herbert W Ohm, Stephen J Molnar, Graham J Scoles, Peter E Eckstein, J Michael Bonman, Alf Ceplitis, Tim Langdon

**Affiliations:** 1 Agriculture and Agri-Food Canada, ECORC, K.W. Neatby Bldg., 960 Carling Ave., C.E. Farm, Ottawa, ON K1A 0C6, Canada; 2 Diversity Arrays Technology P/L, 1 Wilf Crane Cr., Yarralumla, Canberra, ACT 2600, Australia; 3 USDA-ARS & University of Minnesota, Dept. of Agronomy and Plant Genetics, 411 Borlaug Hall, 1991 Upper Buford Circle, St. Paul, MN 55108, USA; 4 Norwegian University of Life Sciences, Department of Plant and Environmental Sciences, N-1432 Ås, Norway; 5 Institute of Biological, Environmental and Rural Sciences, Aberystwyth University, Plas Gogerddan, Aberystwyth, SY23 3EB, UK; 6 USDA-ARS, Robert W. Holley Center for Agriculture and Health, Cornell University Dept. of Plant Breeding and Genetics, 407 Bradfield Hall, Ithaca, NY 14853, USA; 7 USDA-ARS, Purdue University, Agronomy Department, 915 W. State St., West Lafayette, IN 47907-2054, USA; 8 Department of Plant Sciences & Crop Development Centre, University of Saskatchewan, 51 Campus Drive, Saskatoon, SK S7N 5A8, Canada; 9 University of Minnesota, Dept. Of Agronomy and Plant Genetics, 411 Borlaug Hall, 1991 Upper Buford Circle, St. Paul, MN 55108, USA; 10 Cornell University, Dept. of Plant Breeding and Biometry, 252 Emerson Hall, Ithaca, NY 14853-1902, USA; 11 USDA-ARS, 1691 South 2700 West, Aberdeen, ID 83210, USA; 12 Svalöf Weibull AB, Cereal Breeding Department, S-268 81 Svalöv, Sweden; 13 University of Illinois, Department of Crop Sciences, 1102 S. Goodwin Avenue, Urbana, IL 61801, USA; 14 Department of Plant and Environmental Sciences, Carl Skottsbergs gata 22B, Box 461, Gothenburg University, SE40530 Göteborg, Sweden; 15 Universidade Federal do Rio Grande do Sul, Departamento de Plantas de Lavoura, Caixa Postal 776, 91.501-970 – Porto Alegre – RS, Brazil; 16 USDA-ARS, Cereal Disease Lab, & University of Minnesota, 1551 Lindig Ave., St. Paul, MN 55108, USA; 17 Department of Agronomy, 915 W. State St., Purdue University, West Lafayette, IN 47907-2054, USA

## Abstract

**Background:**

Genomic discovery in oat and its application to oat improvement have been hindered by a lack of genetic markers common to different genetic maps, and by the difficulty of conducting whole-genome analysis using high-throughput markers. This study was intended to develop, characterize, and apply a large set of oat genetic markers based on Diversity Array Technology (DArT).

**Results:**

Approximately 19,000 genomic clones were isolated from complexity-reduced genomic representations of pooled DNA samples from 60 oat varieties of global origin. These were screened on three discovery arrays, with more than 2000 polymorphic markers being identified for use in this study, and approximately 2700 potentially polymorphic markers being identified for use in future studies. DNA sequence was obtained for 2573 clones and assembled into a non-redundant set of 1770 contigs and singletons. Of these, 705 showed highly significant (Expectation < 10E-10) BLAST similarity to gene sequences in public databases. Based on marker scores in 80 recombinant inbred lines, 1010 new DArT markers were used to saturate and improve the 'Kanota' × 'Ogle' genetic map. DArT markers provided map coverage approximately equivalent to existing markers. After binning markers from similar clones, as well as those with 99% scoring similarity, a set of 1295 non-redundant markers was used to analyze genetic diversity in 182 accessions of cultivated oat of worldwide origin. Results of this analysis confirmed that major clusters of oat diversity are related to spring *vs*. winter type, and to the presence of major breeding programs within geographical regions. Secondary clusters revealed groups that were often related to known pedigree structure.

**Conclusion:**

These markers will provide a solid basis for future efforts in genomic discovery, comparative mapping, and the generation of an oat consensus map. They will also provide new opportunities for directed breeding of superior oat varieties, and guidance in the maintenance of oat genetic diversity.

## Background

Oat is a cereal crop of global importance used for food, feed, and forage. It is adapted to cool climates and is cultivated predominantly in temperate regions or in winter seasons. Most cultivated varieties of oat belong to *Avena sativa *L., an allohexaploid species with 2n = 6× = 42. Other species in the genus *Avena *have ploidy levels ranging from diploid to hexaploid [1,2], and some of these species have been used as sources of new traits for cultivated oat.

In oat, as in other crop species, there is growing recognition of the need to identify patterns of global genetic diversity, and to use this information in concert with tools for genomic discovery and molecular breeding. Genetic diversity (and associated population structure) depends largely on historical patterns of deliberate and passive efforts to create improved crop varieties [3,4]. For pragmatic reasons, most oat breeders tend to favour crosses among locally adapted varieties, which may erode genetic diversity within a breeding program and create geographically-dependent population structures. Patterns of diversity also develop as a result of breeding objectives that are targeted toward specific adaptations and commercial uses. Both spring and winter forms of *A. sativa *exist, but the characteristics that define winter oat (requirement for vernalization and tolerance to freezing) are expressed to varying degrees, and many winter oat varieties can also be grown as spring-seeded annuals. Another distinction is made between varieties with groats (oat kernels) that thresh free from their hulls (hulless, or naked oat) *vs. *those with hulls that adhere to the seed (covered oat). Further distinctions are made based on hull colour and grain composition, and these characteristics can be relevant to commercial use or adaptation. However, most types of hexaploid oat are fully cross-fertile, and cross-hybridizations are made among different categories to varying degrees by different breeders.

A study of AFLP markers in a core set of cultivated oat germplasm [5] indicated that most genetic relatedness in cultivated oat is associated with geographical origin and with the presence of a distinct, red-seeded, *byzantina*-type that has sometimes been considered as a separate species or subspecies. Although the hulless character results in a distinct market class, this trait is affected primarily by a single locus [6], and frequent inter-mating among covered and hulless types has apparently reduced this as a factor in population structure. A distinction between spring and winter types was not made in the above study [5], but parallel development of spring and winter types within the same breeding program is rare. Cross-hybridization of spring and winter types is not common, due to the genetic complexity of these different adaptations, and also due to the technical difficulty in hybridizing varieties with different flowering times.

Modern genomics research in oat was inaugurated in 1992 with the publication of the first RFLP map in diploid *Avena *[7]. This was followed by original and updated versions of hexaploid maps based on the 'Kanota' × 'Ogle' (KxO) recombinant inbred line (RIL) population [8,9] and by the addition of new sets of mapped markers [10-16]. Many additional maps, both partial and complete, have been published in hexaploid oat, as reviewed by Rines *et al*. [17] and compiled in an online database [18]. However, most maps contain very few markers that are shared among other populations. The KxO map contains the most complete set of markers and has been the primary reference for comparative map analysis in oat. However, the KxO population presents some challenges as a reference population; notably, the population is relatively small, and contains at least one major translocation [19] that causes clustering and pseudo-linkage of markers from two different chromosomes [9]. Unlike wheat, where a combination of consensus mapping and physical mapping has resolved linkage groups that correspond to 21 chromosomes [20], efforts in oat have not yet produced a true consensus map in which all linkage groups are assigned to the expected 21 oat chromosomes.

The current arsenal of molecular markers in oat is based on technologies that include SCAR, SSR, AFLP, and RFLP. Unfortunately, this diversity of technologies creates difficulties in performing comparative genomics within the oat community. Technologies based on PCR are the easiest to implement, but they require a multitude of different primers, and conditions for amplification may need to be re-optimized in different laboratories. Efforts to increase the availability of SSR markers in oat are ongoing, but only a limited number of these markers have been published, and only a subset of these are polymorphic in any given oat population [10,16,21].

The above factors highlight the urgent need for a set of molecular markers that provide complete genome coverage, that are based on a homogeneous technology, and that can be scored readily in new germplasm by any member of the global oat research community. Such a resource would accelerate the development of new maps, and would allow the integration of existing maps into a single consensus map. It would also allow oat researchers to conduct routine marker analysis for breeding applications, for mapping novel traits, for studying genetic diversity, and for other diagnostic applications. Future advances in oat research, including sequencing and functional genomics, will depend on the availability of a robust consensus map containing reliable markers that can be scored on a high-throughput basis. Furthermore, there is mounting evidence that whole genome association studies can yield informative results in an inbreeding species such as wheat [4], and this strategy has shown good potential in oat [22]. This work would benefit greatly from increased map coverage, and from markers that can be scored efficiently and consistently.

Recently, a novel technique for the development and application of microarray-based molecular markers has been described [23]. The patent for this technique, known as diversity array technology (DArT), is licensed freely under an open-source model [24]. DArT has been applied successfully in several crops including barley [25] and wheat [26], and information on the current status of technology development is available online [27]. Briefly, the DArT technique is based on isolating a random set of cloned DNA fragments from a complexity-reduced, pooled DNA sample. These clones are arrayed on a solid phase slide, where they selectively hybridize to complexity-reduced, PCR-amplified, genomic samples. Differential hybridization is usually a result of single-nucleotide polymorphisms (SNP) that affect the presence of restriction sites (and, therefore, certain amplified fragments) in the complexity reduction. A major advantage of DArT is that it provides a consistent high-throughput method whereby a complete set of markers with full genome coverage can be surveyed in parallel across many genomic samples. Because this technology is based on a set of cloned DNA fragments, these fragments can be sequenced and made accessible to the international research community. Furthermore, since the technology is freely available, the assay can be performed by an experienced provider at reasonable cost. However, the prerequisite for DArT analysis is the development and validation of a diagnostic DArT array.

The objectives of this study were: (1) to develop and describe a set of DArT markers giving complete genome coverage in hexaploid oat, (2) to revise and improve a hexaploid oat linkage map through incorporation of these new markers, and (3) to use these markers to analyse genetic diversity in a global collection of oat germplasm. Because this is the first report describing DArT marker analysis in oat, we also provide a detailed set of additional reference material to support future analyses utilizing these markers. Throughout this study, we refer to oat germplasm accessions collectively as 'varieties', regardless of whether they are cultivated varieties (cultivars), breeding lines, or experimental varieties.

## Results and discussion

### DArT marker development

Before describing the results of the mapping and diversity analyses, we present a general account of DArT marker discovery in oat and a characterization of the clones on which these markers are based. The objectives within this topic are: (1) to describe general results of DArT polymorphism analysis and discuss details necessary for future work based on these markers, (2) to characterize redundancy among DArT markers and the associated clones, (3) to characterize the sequences and potential gene content of DArT clones, (4) to describe the resulting data sets upon which the mapping and diversity analyses were based.

#### Clone generation, array production, and hybridization

After initial tests of five complexity reduction methods through gel electrophoresis of amplified products (data not presented), the '*Pst*I/*Taq*I' method was chosen for library construction. Genome complexity was reduced by double digesting DNA with the restriction enzymes *Pst*I and *Taq*I while simultaneously ligating adaptors complementary to the *Pst*I overhangs. This was followed by PCR amplification of intact *Pst*I (without the *Taq*I site) fragments having adaptors ligated at both ends. Interestingly, this complexity reduction method is among the most successful ways of generating genomic representations for DArT from many plant species for which this technology has been adapted, including barley [25], wheat [26], cassava [28], and *Arabidopsis *[29]. Concerns regarding the stable inheritance of markers generated using *Pst*I, a methylation sensitive enzyme, have been addressed in earlier work done on barley [25], where markers were stably inherited across developmental stages and environments. In the same study [25], the construction of a high density consensus map was based on allelic states that were maintained consistently through several rounds of growing DH lines in various environments. In that work, there were no substantial changes in allelic state or appearance of double crossovers, either of which would have indicated a significant resetting of methylation status. In the current work, there were also no observable symptoms of methylation changes, such as map expansion. Nevertheless, we recommend ongoing monitoring of DArT-based results to flag any markers that demonstrate symptoms of instability.

The DArT marker discovery phase took place over a period of several months and produced five independent DArT clone libraries. Three libraries were derived from cultivated materials originating from diverse countries, one was from a mixture of wild and cultivated materials, and one was primarily from wild oat relatives. The pooled DNA used to construct all cultivated material libraries contained 60 oat varieties from diverse countries of origin (see Table 1 and Additional file 1). The inserts generated from the clones in these libraries were arrayed on three separate discovery arrays (identified in Additional file 2), each containing 6144 clones arrayed in duplicate.

**Table 1:** Countries, country codes (CC), and number of varieties (N) from each country (further details are listed in Additional file 1)

**Country**	**CC**	**N**	**Country**	**CC**	**N**	**Country**	**CC**	**N**
Algeria	AR	1	Ecuador	EC	1	Norway	NO	8
Argentina	DZ	1	Finland	FI	5	Portugal	PT	1
Australia	AU	4	Germany	DE	3	Sweden	SE	15
Brazil	BR	9	Netherlands	NL	1	UK	GB	23
Canada	CA	42	New Zealand	NZ	1	USA	US	67

We also tested a number of assay components for the ability to increase data quality in oat (data not presented), but the methods established earlier for several other cereals proved to be equally efficient in oat. However, the first array (Array I) did not perform as well as Arrays II and III. While the composition of Array I (approximately 2,000 clones from wild relatives) contributed to this poorer performance with cultivated materials, there was also a negative contribution from a set of approximately 100 clones containing tandem repeat sequences (discussed later). The presence of DNA derived from these clones on the array resulted in overly strong fluorescent signals for accessions containing the corresponding sequences. We managed to improve the performance of Arrays II and III by eliminating most clones containing the tandem repeat sequence using the restriction enzyme *Ssp*I, for which there was a recognition site in most of the repeats (data not presented).

#### Scoring of DArT polymorphisms

After primary quality filtering by established methods (as *per *methods and [26]), 1958 DArT markers were scored across a diversity panel of 182 cultivated germplasm accessions, and 1010 DArT markers were scored on 80 RILs from the KxO population (Additional file 2). Due to differences in quality-filtering, 241 of the 1010 markers scored on the KxO population were not among the 1958 markers scored across the diversity panel. This reflects two sources of increased precision in the mapping population. Firstly, the DNA samples in this set came from a single homogeneous set of laboratory isolations, rather than from several isolations at various laboratories, as was the case for the diversity samples. Secondly, because alleles in the mapping population segregate at approximately equal frequencies, the bimodality of the signal intensity among tested targets (the basis for DArT marker discovery) is more reliably detected as compared to diversity panel analysis, where markers with minor alleles at low frequencies are not discovered as efficiently. The union of the two sets of marker scores included 2199 distinct markers. Of these, 504 were based on clones from the first array, 749 from the second, and 946 from the third. A larger set of 2688 markers (see Additional file 2) was selected for future work based on relaxed quality-filtering thresholds. These additional markers were included in the sequence analysis work, and will be used in the development of a second generation genotyping array by DArT P/L [27].

#### DArT clone sequence analysis

Seven 384-well plates of DArT clones showing putative polymorphism were sequenced in both directions, yielding 5376 sequence reads from 2688 clones. Of these, 4907 sequences remained after quality trimming and vector clipping, and these were merged into 2237 paired assemblies, leaving 433 unmatched single reads. This provided a total of 2670 non-redundant sequences corresponding to 2573 unique clones, 97 of which had two unique reads, and 239 of which had a single unique read (see Additional file 3). Trimmed sequences less than 20 bp in length were ignored, but 88 sequences less than 50 bp in length were retained for further analysis. The average length of the 2670 non-redundant sequences was 496 bp, while the average length of the 2237 two-sequence builds (an estimate of the average DArT clone length) was 533 bp.

The 2670 unique sequences have been submitted to the NCBI GSS division (GenBank accession numbers FI157274 through FI159943, to be released upon publication), with clone identifiers corresponding to the DArT marker naming convention presented in this report. Clone identifiers that are appended with an M or T indicate multiple un-matched reads from the same DArT clone. Secondary identifiers (GSS#) were formed from each clone name appended with a number indicating its membership in sequence assembly A3 (see below).

#### DArT clone sequence assembly

**Figure 1:**
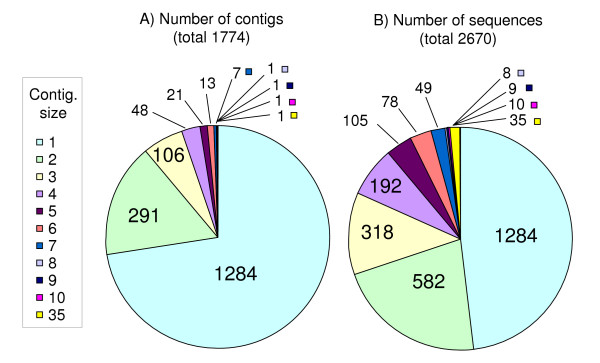
**Sequence assembly**. Assembly of 2670 DArT clone sequences showing (A) number of contig assemblies of different sizes and (B) number of sequences belonging to contigs of different size.

Sequences for 2670 DArT clones were assembled at three different levels of stringency. In general, the assembly conducted with an 80% similarity criterion showed the best agreement with clusters based on marker scores (see Additional file 2, and later discussion of marker bins), indicating a tolerance for a moderate level of sequence divergence in diagnosing identical marker loci. We have, therefore, used the 80% sequence assembly (A3) for all subsequent analyses. Based on the A3 assembly, there were 1774 unique classes of DArT clones, including 1284 clones giving unmatched singletons (Figure 1A). However, slightly more than half of the 2670 sequences fall into multi-sequence contigs (Figure 1B), indicating a moderate level of redundancy in DArT marker identity. The analysis of SNP and SSR polymorphisms within DArT sequence assemblies will be part of a future study. Consensus sequences and singletons from the non-redundant set of 1774 contigs (Figure 1A) were used in subsequent analyses.

Although a detailed analysis of sequence assemblies is beyond the scope of this paper, it is relevant to discuss whether the assemblies are likely to contain sequences from duplicated or homoeologous regions. This could affect the probability of success in developing new SNP or microsatellite markers directly from the sequences presented in this study. Firstly, it is important to note that the complexity reduction step in DArT analysis is the same step that is applied in clone discovery. This step amplifies a very small proportion of the genome. Although co-amplification of homoeologous loci may be favoured, one would still expect to generate a large number of single-locus clones. Secondly, all of the sequenced clones belong to markers that have been selected based on stringent criteria that favour non-duplicated loci. These criteria are discussed further under the heading "Presence and effects of homoeologous loci". An inspection of the assemblies does reveal several patterns of polymorphisms that might arise from homoeologues, but the sequencing depth is not adequate for any conclusions to be drawn. Furthermore, since all sequences represent anonymous genotypes that could belong to any of the 60 entries that were used for clone discovery, a polymorphic sequence that looks strikingly different from others within an assembly could simply belong to a more diverse genotype. A deeper sequencing or re-sequencing effort will be required to explore this question thoroughly.

#### High frequency of tandemly-repeated sequences

Although the number of DArT clone sequences per contig tapered off at about 10 (Figure 1A), one contig contained 35 DArT clone sequences (identified as A3_19 in additional files and in Genbank submissions). The number of sequences in this contig would have been greater had this not been addressed early in the marker development phase (see earlier discussion). Sequences belonging to this contig were characterized by varying numbers of tandemly-repeated 171 bp elements. The frequency at which this sequence was isolated possibly results from the presence of one or more highly-repeated regions in the oat genome with a common occurrence of *Pst*I restriction sites. Although these sequences may have been isolated from multiple genomic regions, the diagnostic DArT polymorphisms associated with these clones appear to be limited to a single genetic locus. This is supported both by a nearly-identical pattern of scores in the diversity analysis (near complete linkage dissequilibrium), as well as by a single map location (reported later). These redundant sequences share some structural similarity (tandem arrangement of repeats and the unit length) with the so-called telomere-associated sequences (TAS) reported in barley by Kilian and Kleinhofs [30]. While this sequence occurs at a tightly linked series of loci that are not associated with a telomere in the oat genome, a number of TAS loci were also located interstitially in barley [31]. Alternatively, this tandem repeat sequence may be associated with an oat centromere, as similar length tandem repeats have been identified as components of centromeres in many organisms, including human (171 bp alphoid repeat) and many plant species [32]. More detailed analysis of these sequences and the associated loci in oat will be the subject of a separate study.

#### BLAST analysis of DArT clone sequences

**Figure 2:**
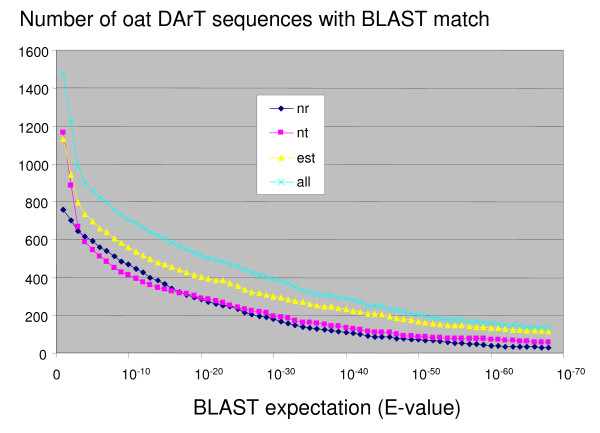
**BLAST similarity**. Number of non-redundant DArT clone sequences (consensus from contigs plus singletons) from a set of 1774 with BLAST hits having E-values smaller (more significant) than a given value when tested against the non-redundant nucleotide database (nt), the non-redundant protein database (nr), the EST database (est) or the concatenation of all three (all). All BLAST databases were downloaded from NCBI [33] on May 6, 2008, and searches were performed locally using BLASTX and BLASTN 2.2.18 [47]

The non-redundant set of 1774 unique sequences (consensus sequences plus singletons) was searched against the complete non-redundant protein (nr), DNA (nt), EST, and Swissprot databases on NCBI [33] (downloaded on May 6, 2008). A compilation of the best BLAST hits from each searched database is shown in Additional file 4. Figure 2 illustrates how the number of sequences with BLAST hits depends on the significance criteria that are applied, allowing the reader to visualize the number of potentially annotated sequences at varying levels of stringency. Although the Swissprot database often provides a well annotated hit, it is the least complete of the databases and is therefore not represented in Figure 2. The number of significant BLAST hits at high levels of significance demonstrates that a large proportion of these sequences are homologous to known or predicted gene sequences. For example, at an expectation (E-value) of 10^-5^, approximately half of the 1774 sequences had significant BLAST matches, while an expectation of 10^-10 ^identified 705 matches. Of the matches at E < 10^-10^, 471 showed similarity to a protein sequence based on a translated search. A detailed functional annotation of these genes has not yet been attempted. However, given that the oat genome probably has a low gene density similar to those of wheat and barley, the high frequency of gene similarity among these DArT clones provides a good indication that many DArT markers are within genes or gene-rich regions. Furthermore, the availability of these DArT clone sequences will provide future opportunities to identify polymorphisms in candidate gene loci, or to isolate series of alleles from these loci.

#### DArT marker redundancy and generation of consensus markers

**Figure 3:**
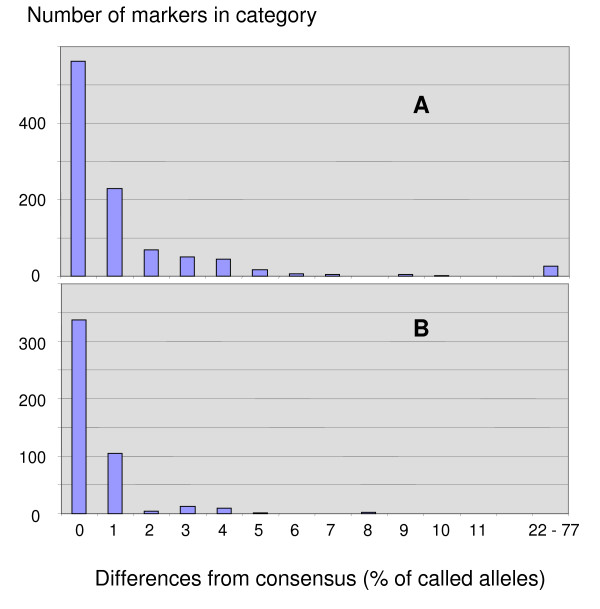
**Markers with potential scoring errors**. Frequency distribution of percent agreement between called alleles and consensus calls for DArT markers with clones belonging to multi-sequence contigs. For contigs with two members, this is the percent agreement with the other clone in the contig. Part (A) is based on diversity scores for 1015 clones belonging to 431 contigs, and part (B) is based on mapping scores for 485 clones belonging to 194 contigs.

As expected, some DArT markers with unique DNA sequences gave similar or identical scores in the mapping data, the diversity data, or both. These probably represent markers at separate loci that are closely linked and/or in linkage disequilibrium. Conversely, some markers having highly similar DNA sequences failed to provide identical marker scores. It was shown in wheat [26] that occasional scoring errors occur most often for low-grade markers, and that most of these can be filtered out by adjusting quality parameters or call rate. To address this issue in more detail, we generated and inspected a set of consensus marker scores among members of each contig. Figure 3 shows the frequency of markers for which there are varying numbers of discrepancies between called alleles and a consensus sequence, or with another marker from the same contig. Based on Figure 3A, we assumed that markers in the diversity data that disagreed up to 5% with the consensus calls were providing identical information with occasional miss-scores. For these markers, we used the consensus calls in all subsequent analyses to improve accuracy and reduce redundancy. For markers that disagreed at levels higher than this, we used both markers (in a pair) or the marker and the consensus calls (for those in sets of greater than two). There were 26 markers with diversity scores that disagreed at a level greater than 10%, and 42 that disagreed more than 5%. These markers can be identified by the 'DDif' value within Additional file 2. It is possible that this has resulted from tracking errors; thus, the sequences of these markers should be interpreted with caution, or the clones subjected to re-sequencing if they are used in further research.

For the KxO marker scores (Figure 3B), the maximum error level was also near 5%, but the level of error was rarely greater than 1%. No markers differed from the consensus calls by more than 8%; however, this was true only after correcting the scores for several markers that were apparently scored out of phase with other members of a contig (see paragraph below). Because the consensus marker scores often provided more complete data than scores from individual markers, and because they did not contain ambiguous scores, they were used preferentially in the construction of the framework map (described later).

#### Scoring precision of DArT markers

The number of apparent mis-scored markers (depicted in Figure 3) may give a misleading perception of the scoring precision of these DArT markers. Based on the cumulative frequencies presented, we can estimate the overall frequency of scoring errors. For the diversity set, there are 785 individual cases where an allele call disagrees with the consensus or with other markers from the same contig. This applies to 1015 markers, for which there are 158789 non-missing scores. For the mapping set, there are 208 cases where an allele call disagrees as above, which applies to 470 markers with 373,261 non-missing scores. In both cases, the derived error rate is very close to 0.5%.

This estimate of error rate was generated from discovery-stage experiments that were conducted over an extended time period using three different arrays. Thus, some scoring errors may have arisen because of the heterogeneity of conditions encountered when using more than one array, and because the discovery arrays are not subject to the same level of rigorous quality control as the routine genotyping arrays. The DArT arrays that will be used in future experiments will contain a composite collection of polymorphic clones on a single array, such that hybridizations are done only once. Furthermore, the experimental material will generally be more homogeneous than the material used in this study. In particular, the DNA samples used in the diversity panel of this study were prepared at eight different laboratories, so differences in DNA quality may also have contributed to the lower reliability of the scores. Furthermore, the allele calling parameters are often set at higher stringencies in experiments that follow the discovery stage. The allele-calling procedures and parameters typically used at DArT P/L [27] have been designed and tested to give an average error rate of 0.3%, and this rate has been stable across genotyping arrays for a variety of crop species over several years [25,26]. Thus, we can expect that future work with oat DArT markers will have a similar level of quality, with an error rate lower than determined in this study.

The above estimates of error rate apply to the precision of calling a genotype that is either 'allele present' or 'allele absent'. This does not take into account different types of error which could arise from the presence of homoeologous duplicated loci. The latter issue is discussed more thoroughly under the later section "Presence and effects of homoeologous loci".

#### DArT marker binning and the selection of markers for diversity analysis

To facilitate the identification of similar markers, and to compare this with information from sequence analysis, we constructed groups of markers (bins) based on marker scores from both the diversity and the mapping data, including the contig-based consensus markers (shown in Additional file 2). In general, bins based on a 1% similarity of scores showed the best agreement with sequence assemblies and map positions, and we chose this level of binning for subsequent analyses. In some cases, markers in the same bin contained clones belonging to different sequence assemblies. This was likely due to the presence of tightly-linked markers that are revealed by distinct clones. For this reason, it will be useful to inspect these groups any time there is a need to find additional tightly-linked but distinct markers for fine-mapping purposes.

To refine this binning, and to create a non-redundant set of scores for genetic diversity analysis, we also binned the markers based only on diversity scores, and selected the single marker with the most complete data, or a consensus marker when present, to represent each bin (also shown in Additional file 2). We further inspected each bin, and increased the representation under the following conditions: (1) if markers within a bin represented more than one map position, and if a marker had at least one different score from other representative members of the bin, (2) if markers were not mapped, but the bin contained members from multiple contigs, and there were at least two score differences from other representative members of the bin.

#### Identification of marker phase in segregating progenies

For most markers, the parental phase in the Kanota and Ogle mapping parents was known, and +/- marker scores were converted to the scoring convention used by various software packages. However, the parental phase for 98 markers with segregating polymorphisms was unknown. This was either because marker alleles were not called in either parent (27 markers), or because both parents showed the same genotype (71 markers). When sequences of the corresponding clones belonged to contigs that contained markers with known phase, the phase was inferred from those markers. However, when the phase was completely unknown, two separate copies of the marker were scored in opposite phase and appended with the suffix "_rp" or "_rp2". One copy was then identified as being in the correct phase based on map placement, but a few markers with these extensions remain in the additional files when the correct phase could not be inferred with certainty. Most of the markers with identical scores in Kanota and Ogle had the 'plus' allele in both parents. It was later noted that the 'Ogle_1040' entry (a reselection of Ogle, typed in the diversity analysis but not in the marker analysis) had scores that agreed better with the segregation pattern in the KxO progenies, confirming that this entry is more closely related to the original Ogle parent used in the KxO cross. Furthermore, a large proportion of the markers for which Ogle and Ogle_1040 had different scores were later mapped to a region on KxO linkage group 4_12_13 between cM position 139 and 159. Thus, the two Ogle sister lines could be useful in future studies to elucidate effects of QTL within this region.

### Molecular mapping

**Figure 4:**
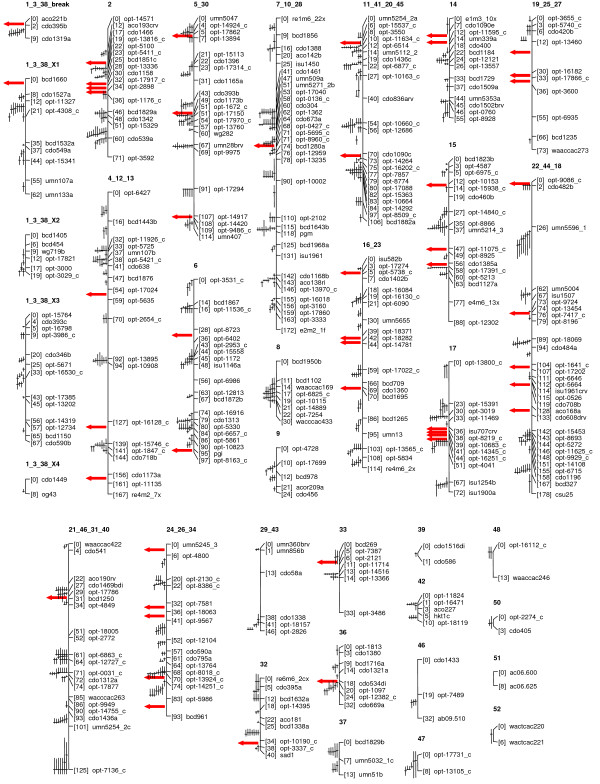
**KxO Linkage map**. A framework version of the new 'Kanota' × 'Ogle' linkage map showing placement of additional markers (cross-hairs). Vertical bars on cross-hairs indicate the tendency of a placed marker to stretch the interval. The bars are 1/4 of the length that the interval would be if the marker were placed at this position. Where the number of crosshairs exceeded ten they were replaced with a red arrow. A high-resolution multi-page version of this figure is presented in Additional file 7. Additional details and a complete listing of placed marker names are found in Additional file 8.

Molecular mapping was conducted using a combined data set that included 1010 DArT markers and 287 markers that constituted the framework of the previous map [9] (see data set in Additional file 5). The preliminary data set that was used contained additional DArT markers scored in opposite phase (see above), but these have been reconciled where possible. Although the first *de novo *map produced using EasyMap (not shown) agreed well with the previous map [9], there were sufficient differences to warrant further exploration. This was done using JoinMap [34], as discussed in the methods section, to arrive at a consensus that was compatible with most available data and mapping strategies. The previous map was constructed using two different software packages (Mapmaker [35] and Gmendel [36]); thus, the new map benefits from construction using four different software packages. A detailed version of the resulting map showing correspondence to the previous map is shown in Additional file 6. A framework version of the new map was produced to streamline further analysis and comparative mapping. The framework version, showing major features and representative markers, is presented in Figure 4 and Additional file 7. An HTML-formatted version of this framework map is also provided in Additional file 8. This version contains marker placements for all markers previously published by Wight *et al*. [9], as well as for 119 markers that have been published more recently [10-16],. It is our intention to integrate this map into the international database 'GrainGenes' [37], and to assist GrainGenes staff by providing ongoing updates to the information presented in Additional file 8. We chose to continue a concatenated numbering convention for linkage groups in the KxO map because future mapping efforts may consolidate further groups, and we do not wish to confuse the community with an additional interim naming convention.

Although the major structure of the new map agrees substantially with that of previous maps (Additional file 6), the current map provides new evidence for joining previous linkage group fragments, as well as for revising the order of some linkage groups (Table 2). The most substantial difference in the newly-presented map is that the linkage group previously published as "KO_3+38" has been deliberately broken into several sections to discover linkage group fragments that may belong to translocated chromosome arms. Chromosomal interchanges among hexaploid oat genotypes are well known [38], and it has been confirmed that most spring oat genotypes (including Ogle) contain a reciprocal intergenomic translocation involving chromosomes 7C and 17, whereas non-translocated versions have been found in many North American red-oat types (including Kanota) [39]. The postulated effect of this translocation on the KxO map is to suppress recombination near the interchange breakpoint due to the formation of a quadrivalent meiotic structure. Non-lethal meiotic interchanges on the arms of this quadrivalent structure can produce recombination events along the four separate linkage group arms, resulting in four linear series of recombination events, all with statistical linkage to a single recombination-suppressed breakpoint. This would properly be recorded as an 'X'-shaped linkage map; however, software written to test this [40] has not produced conclusive results in KxO. Because of the strong likelihood that more than two linkage groups are associated with this breakpoint, we deemed it useful to deliberately isolate markers in the breakpoint region as a separate linkage group to allow generation of multiple additional groups. This strategy appears to have had the intended effect, because there are now five separate linkage groups (including the breakpoint region) formed from markers previously assigned to linkage group 3+38, as well as the markers previously found in group KO_1 (considered to be a homoeologous group by Wight *et al*. [9]). Although this is a potentially useful dissection of meiotic linkage groups, it should not be considered as being conclusive regarding the physical arrangement of markers on the translocated arms. Further mapping of these markers in populations lacking the translocation difference should be conducted to resolve this issue.

**Table 2:** List of linkage groups in 'Kanota' × 'Ogle' (KxO) oat framework map

**ID**	**LG Name**	**cM**	**Notes and comments**
1	1_3_38_break	9	Breakpoint region of group 3+38, includes markers from group 1
1.1	1_3_38_X1	62	Section of group 3+38, includes markers from group 1
1.2	1_3_38_X2	19	Section of group 3+38, includes markers from group 1
1.3	1_3_38_X3	67	Section of group 3+38, includes markers from group 1
1.4	1_3_38_X4	8	= group 38 portion of group 3+38
2	2	71	No new joins
4	4_12_13	167	4_12 joined previously via aneuploid evidence, 12_13 join together via DArT markers
5	5_30	115	Some previous evidence for this, but treated as homoeologues by Wight et al. (2003). Join together via DArT markers
6	6	97	No new joins
7	7_10_28	172	No new joins
8	8	32	No new joins
9	9	24	No new joins
11	11_41_20_45	106	11_41+20 joined previously via aneuploid and mapping evidence, 20_45 join together via DArT markers
14	14	55	No new joins
15	15	88	No new joins
16	16_23	114	No new joins
17	17	72	No new joins
19	19_25_27	107	19+27 joined previously via mapping evidence, 27_25 join together via DArT markers
21	21_46_31_40	125	No new joins
22	22_44_18	182	No new joins, but order of 44 and 18 reversed
24	24_26_34	92	No new joins
29	29_43	45	No new joins
32	32	40	No new joins
33	33	33	No new joins
36	36	32	No new joins
37	37	13	No new joins
39	39	1	No new joins
42	42	10	No new joins
46	46	32	New group
47	47	8	New group
48	48	13	New group
50	50	3	New group
51	51	8	New group
52	52	6	New group

**Figure 5:**
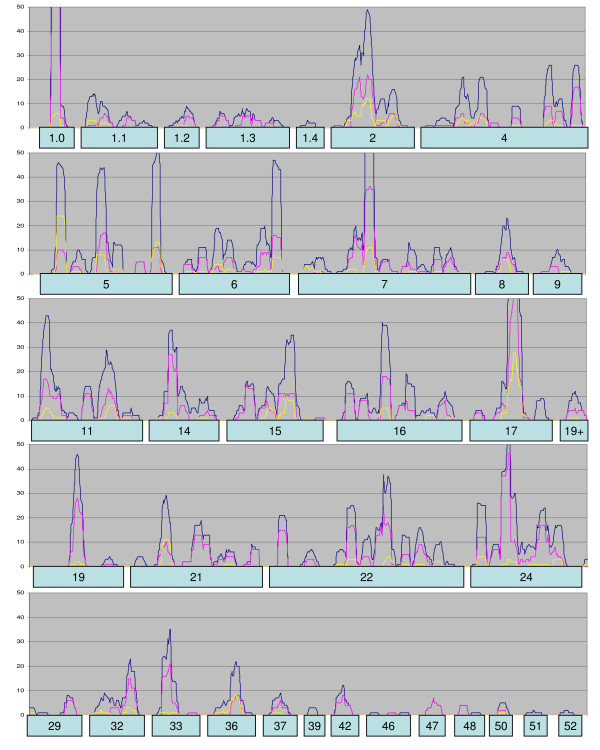
**Marker clustering in linkage map**. Smoothed density of markers within a 10 cM moving window on the 'Kanota' × 'Ogle' framework linkage map. The dark blue line shows the overall density of markers including DArT markers and those mapped prior to 2003. Magenta shows the distribution of DArT markers alone. The yellow line shows the density of AFLP markers mapped prior to 2003.

Past studies of DArT mapping in other species, including the development of a high-density DArT-based consensus map in barley [41], have indicated that DArT markers have a reasonably uniform distribution within plant genomes. To address whether this is also true in oat, we produced a diagram showing the density of DArT markers in each region of the current oat linkage map (Figure 5). Although DArT markers are highly clustered, the locations of these clusters correspond almost exactly to the locations of other clustered markers. It is proposed that many of these clusters represent regions where large physical distance corresponds to small genetic distance, such as in centromeric regions. However, it is likely that some clusters also represent additional regions where chromosomal rearrangements have occurred [9] and, furthermore, that some regions contain other characteristics (such as high gene density) that cause a higher frequency of marker discovery. Because the mapped DArT markers show approximately 50% redundancy (see earlier sections), the distribution of unique loci is considerably more uniform. Only a few regions of the map did not contain any DArT markers. These include short sections of linkage groups 1.1, 1.4, 7, 9, 17, 21, and 29. Although these may represent regions that cannot be addressed genetically using DArT markers, they may also represent regions where other marker types are disproportionately frequent. In contrast, the distribution of approximately 300 previously mapped AFLP markers was highly irregular (Figure 5).

The current KxO oat linkage map should be considered as a map in transition. Although current efforts with DArT markers have improved the density of coverage and the accuracy with which linkage groups are represented, there remains considerable work to be done by the oat community to bring this effort toward producing a stable consensus map of hexaploid oat and assigning linkage groups to physical chromosomes unambiguously. Because the new DArT marker set can be scored quickly and efficiently in new populations, we expect that this will accelerate these efforts. For example, several of the authors are already engaged in refining existing maps and in generating new maps using DArT markers, and the intention is to collaborate in the development of a consensus map based on these efforts. In the interim, the availability of DArT markers and their related sequences will enhance the ability of the oat community to identify and compare locations of QTL and other genetic loci in oat.

### Diversity and pedigree analysis

**Figure 6:**
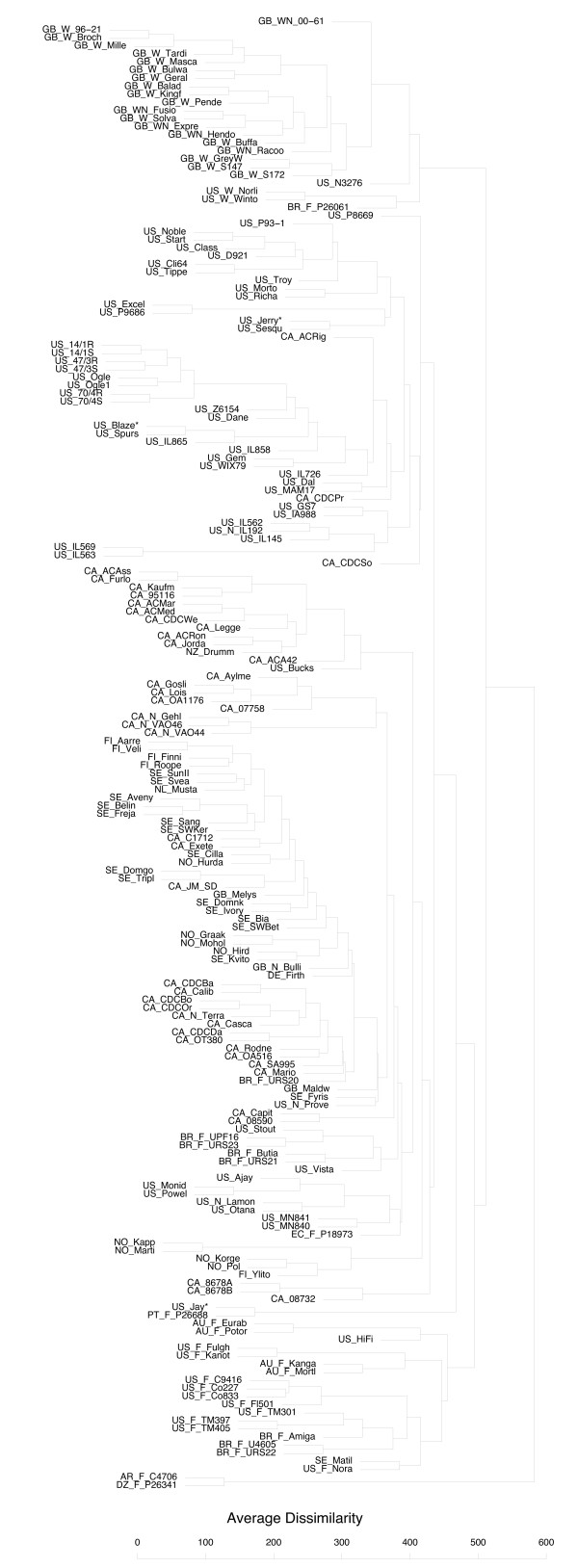
**Cluster analysis of varieties**. Agglomerative clustering using the un-weighted pair group method with averages (UPGMA) for 134 oat varieties using 1295 non-redundant DArT marker loci. Oat varieties are identified by truncated entry names, which are preceded by two-letter codes indicating country of origin, and by additional codes if they are winter-types (W), fall-sown but winter grown (F), or naked (N). See Additional file 1 for full entry names and country of origin codes. Clustering was implemented using the Agglomerative Nesting (AGNES) function in the R statistical environment [52]. The distance metric used was the Manhattan distance, which represents (in this case) the number of loci (out of 1295, adjusted for missing scores) for which two lines differed in their marker score. A high-resolution version of this figure is presented in Additional file 10.

**Figure 7:**
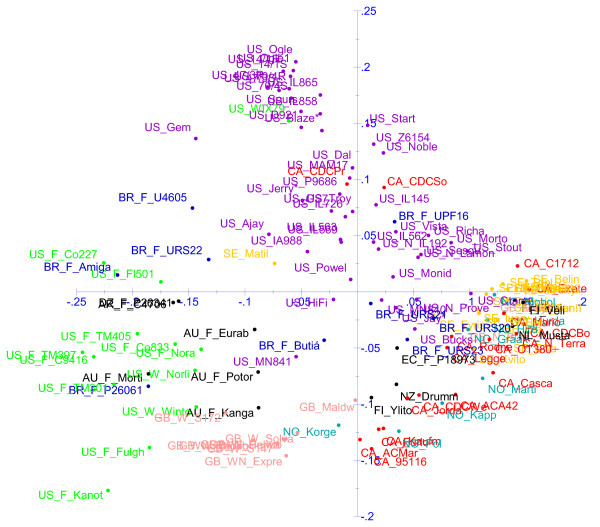
**Principle coordinate analysis (PCA)**. Plot of PCA axes 1 and 2 based on Manhattan distance calculations for 134 oat varieties using 1295 non-redundant DArT marker loci. Oat varieties are identified by truncated entry names, which are preceded by two-letter codes indicating country of origin, and by additional codes if they are winter-types (W), fall-sown but winter grown (F), or naked (N). See Additional file 1 for full entry names and country of origin codes. Selected counties of origin are coloured blue (Brazil), red (Canada), pink (Great Britain), light-blue (Norway), yellow (Sweden), purple (USA), or green (USA-Fall/Winter).

Diversity analysis was conducted using a merged data set of 1295 non-redundant markers from three discovery arrays, as discussed earlier (Additional file 9). An UPGMA cluster analysis based on Manhattan genetic distances among 134 varieties with orthogonal data is shown in Figure 6 (Additional file 10), and a principle coordinate analysis (PCA) based on the same data is shown in Figure 7. An extended version of the cluster analysis that includes additional varieties with non-orthogonal data is shown in Additional file 11. We selected these procedures because they are simple and well-known methods that reveal basic population structure, and we have not attempted to construct confidence estimates because it is not our intention to draw definitive conclusions about specific relationships. Alternate distance metrics and clustering methods were also tested, and these tests can be replicated using data from Additional file 9. Our tests with other methods did not reveal major differences, except that Ward's method of clustering produced very homogeneously-sized groups, as it is known to do [42]. This did not seem representative of the irregular-sized clusters that we believed were present in this study, and which were revealed clearly by the UPGMA method.

The known pedigrees of varieties in this experiment were also researched, and these have been recorded in the online oat pedigree database 'POOL' [43,44] with direct links provided from Additional file 1. In many cases, these pedigrees have been traced back more than 10 generations. An UPGMA dendrogram based on empirical computations of genealogical distance is shown in Additional file 12. The distances in this analysis were computed by assuming complete inbreeding of all nodes appearing in a pedigree and absence of selection. Although these assumptions were necessary for the computation of K, they have likely biased the resulting estimates in unknown ways. The analysis in Additional file 12 also shows that 34 of the 182 varieties have no known genealogical similarity with other varieties in this study. In several cases, this is because the pedigrees of proprietary varieties are not available, as they are considered to be intellectual property that cannot be published. In many crop species, this would be the rule rather than the exception. In contrast, the high level of pedigree data in oat results from the fact that many oat varieties have been developed through publicly-funded efforts; thus, pedigree data are shared openly among members of the oat community. Furthermore, many efforts have been made to record and disseminate pedigree data in oat, and these efforts have contributed to the data that are contained in POOL [43,44].

The primary purpose of the diversity and pedigree analysis was to observe the general structure of genetic relationships in oat, and to examine the resolving power of the current DArT marker set. The general structure of the marker-based cluster analysis agrees with many features of the known pedigree matrix. Because marker-based distances are widely acknowledged to be superior to pedigree estimates, we base most overall conclusions on the structure that is revealed by markers. Figure 6 shows clearly that DArT-based clustering has delineated major groups of varieties based on countries of origin and growth habit, while simultaneously separating even the most closely-related varieties. The tendency to cluster based on common origin is most clearly shown for varieties originating from specific breeding programs; for example, the TAMO (Texas A&M) varieties, those from the Coker program (Co), and the Swedish varieties, many coming from the Svalöv Weibull program. Varieties from Norway (NO) were expected to cluster together based on common pedigree (Additional file 12), but there was some divergence among this group, particularly with the variety 'Hurdal', which did not cluster with its parent 'Martin'. Divergences such as this may occur because of differences in selective breeding, but other major divergences from expectations can occur because of seed tracking errors or incorrect pedigree data. Three examples of suspected seed tracking errors ('Jay', 'Jerry', and 'Blaze') have been highlighted in Figure 6 using an asterisk.

The set of winter varieties originating from the UK (GB) are also clearly delineated, and form a separate sub-cluster – both in the pedigree analysis and in the marker analysis. Many of the GB winter varieties can be traced back to an old landrace called 'Grey Winter', which also appears in this sub-cluster. Of interest is that other varieties that are sometimes considered as 'winter' oats bear no relationship to the UK winter varieties. Many of these other varieties are not 'conventional' winter oats, as they are planted in the fall and grown in the winter season in a subtropical climate, in contrast with those that over-winter in a dormant state in a temperate climate. Varieties from the Southern US, Australia, and many parts of South America are winter-grown, and have been coded with the prefix 'F' in Figure 6 to distinguish them from conventional winter types (coded as 'W'). Many of the 'F' types derive some winter hardiness and facultative vernalization requirement from the old landrace 'Red Rustproof' or one of its derivatives, such as 'Fulghum' or Kanota. Of interest is that 'Norline' and 'Wintok' (two of the most winter-hardy varieties from North America, and considered as conventional winter types) also contain Kanota and Fulghum in their pedigrees; yet, based on the marker analysis, they cluster more closely with the UK winter types. This could indicate some selected convergence for winter habit, and also that there is considerable heterogeneity in the landraces from which these varieties are derived.

The clusters shown in Figure 6 reveal many additional relationships which can be pursued in future research. For example, Ogle_1040 and a set of experimental varieties ('US_14/1R', 'US_14/1S', 'US_47/3R', 'US_47/3S', 'US_70/4R', and 'US_70/4S') derived from backcross introgression of crown rust resistance into Ogle are shown to be highly inter-related, but there are still 10 to 100 markers that are polymorphic among these pairs. These markers will be used to study the regions and map locations of introgressed fragments (to be reported elsewhere).

Most of the major country-based groupings and many of the variety-specific anomalies are confirmed by the PCA analysis presented in Figure 7. Although the PCA analysis does not capture all variance, and PCA-based diagrams tend to obscure the labels of adjacent varieties, the colour coding of major countries of origin in Figure 7 provides a good visual representation of how this relates to varietal proximity. This analysis also suggests that spring oat germplasm from the USA is highly diverse and representative of most other types of spring oat germplasm, with the exception of some Canadian and Norwegian germplasm located in the lower right of Figure 7.

### Presence and effects of homoeologous loci

An examination of allele frequency across the raw data sets revealed that the 'plus' allele is present at a frequency of 60% in the diversity data (Additional file 9) and at 54% in the original calls of the KxO data. These figures suggest that there is some systematic bias towards scores that are 'plus' (allele-present) *vs*. those that are 'minus' (allele absent). This bias seems to contradict the idea that mis-amplification would favour erroneous minus-allele scores. However, as discussed previously, the estimated error rate in assigning allele scores is only 0.5%, which would have a minimal effect on the global allele frequency. There are three remaining factors that may have influenced this global allele frequency: (1) a bias against selecting clones with a low frequency of plus-alleles, (2) the presence of heterogeneity or heterozygosity within varieties or progenies, and (3) the possibility of homoeologous or duplicated markers that give indistinguishable plus-alleles.

The first two factors would have a relatively benign influence on the results of this study, but may have contributed much of the bias toward plus-alleles. Since clones were isolated randomly from a pooled population of amplified fragments, it is likely that there has been some systematic bias against clones with a low frequency of plus-alleles; however we are not able to quantify the magnitude of this effect. The presence of heterogeneity and/or heterozygosity may also account for much of this bias. It is already known that the KxO mapping population contains a relatively high frequency of heterozygotes for some markers [9]. Based on 492 codominant RFLP and protein loci that are part of the current mapping set, we have estimated the overall frequency of heterozygotes at 2.6%. We do not know the average level of heterozygosity or heterogeneity in the diversity panel, but since most seed sources were bulked from commercial varieties, and since these were often selected from a generation such as the F_5_, heterozygosity may be as high as 6% for a segregating locus. If we assume that approximately 33% of the tested loci were segregating a given cross, one could expect about 2% of the loci to be heterozygous (or heterogeneous within varieties) in the diversity panel.

Even after accounting for the above factors, we must examine the possibility and the possible effects of independently segregating loci with indistinguishable plus alleles. Such loci are a possibility in any marker technology when applied to a polyploid species such as oat, and they are almost certainly present to some degree in this study. However, there are characteristics of the DArT technology that significantly reduce the number of duplicated loci when applied to a polyploid. A detailed discussion of this is provided in a recent report in wheat [26]. The two most important factors are the genomic complexity reduction, which provides the opportunity for identifying single locus-specific markers, and the intense selection of loci that behave as single locus markers. Before a marker is accepted into a data set, the distribution of standardized intensities for each clone is examined across the complete panel of genotypes using K-means clustering. Distributions of intensity for a single-locus marker are expected to fall into two clusters, with some tolerance for a minor third cluster due to heterozygotes. Loci that deviate from the above model (*i.e. *they show a continuous distribution, a multimodal distribution, or a trimodal distribution with a major intermediate class) are discarded from further analysis because they may represent multiple loci. The K-means classifications are then used to assign marker scores. Although heterozygous genotypes can sometimes be called by this method, we did not attempt to do so in this study, and minor heterozygous clusters were assigned to the plus-allele genotype.

The above routine is highly selective for single-locus markers, and this is validated in the KxO data set, where very few of the loci segregated at frequencies that would support a 3:1 (or higher) segregation ratio. Of the 1010 markers that were originally scored in KxO, 93 loci had allele frequencies greater than 65%, and these are summarized in Additional file 13 for possible further analysis. However, we cannot conclude that these loci are duplicated. Even though a 65% frequency is rejected from a chi-square test of equal frequency at P < 1%, we still expect ten markers to fall into this range by chance. Furthermore, some distorted segregation is due to the presence of heterozygotes, and there are cytogenetic abnormalities segregating in KxO that can also account for distorted segregation [8,9]. We also examined segregating markers for which there were plus-allele scores in both Kanota and Ogle. In principle, these could represent markers that are duplicated. There were 32 markers that met this criterion and these are also indicated in Additional file 13. Interestingly, there was no strong relationship between these loci and the frequency of dominant scores: the average frequency of plus-alleles among these 32 loci was 60%. Thus, it is probable that there are other reasons why these markers segregate in the progenies but not in the parents. One reason is that the parents may differ slightly from the original genotype used in population development. A second reason may be that a marker represents two loci that are tightly linked such that they do not segregate independently.

Even though the behaviour of many markers has been well-characterized in KxO, the evaluation of DArT markers will be an ongoing process as data are gathered for new mapping populations. Importantly, it is possible for a marker to segregate as a single locus in one population, but as a different locus in another population. This would not be detected in the 'first' population such as KxO. In wheat, a hexaploid species for which DArT marker analysis is quite advanced, there has been an ongoing development of consensus maps, and an ongoing assessment of the locations where DArT markers map. This work has spanned approximately 200 different experiments to date. Based on detailed analysis of 2431 DArT markers in 20 of these experiments, it has been estimated that 2282 of these markers map to a single chromosome, 144 (5.9%) map to two chromosomes, and only five (0.2%) map to more than two chromosomes (Triticarte Pty Ltd [45], unpublished). It is premature to make similar estimates in oat, but preliminary data from three additional oat populations have been analysed as described in Additional file 13 to identify 51 oat DArT markers that potentially segregate at multiple locations. Being approximately 5% of the loci that have been mapped, this number shows encouraging similarity to the frequency of multi-locus DArT markers reported in wheat.

Finally, we must address the potential errors that may have resulted from the inclusion of duplicated loci in the analyses reported in this study. As explained above, Additional file 13 contains a list of markers for which there is at least one reason to suspect the possibility of duplicate loci. This list contains 156 markers in total, though many of these are probably 'falsely accused'. To investigate the effect of removing all of these markers from the diversity analysis, we performed a type of analysis that would provide 40 discrete clusters of varieties (see Additional file 14). This was done first using the complete orthogonal data set, then using the same data set after removal of all the markers contained in Additional file 13. The resulting sets of 40 clusters are nearly identical, and each provides further support for the hierarchical clusters presented in Figure 6.

To address potential effects of duplicated loci on mapping, we have highlighted all of the markers from Additional file 13 as character-flags in the HTML-based map shown in Additional file 8. Only 20 of the flagged markers coincided with framework loci, thus the remaining markers did not contribute to the framework of the map and may have been eliminated by mapping algorithms during map construction. Of those that contributed to the framework, many showed segregation distortion, but they mapped with clusters of other loci that also showed segregation distortion. The most pronounced example of this is a region of distorted markers at the 'top' end of linkage group 11_41_20_45, also identified in previous work [9]. It is unlikely that each marker in a cluster would represent an independently duplicated locus, so these clusters more likely represent regions of segregation distortion that occur for other reasons. Several other flagged loci represented cases where both parents had a plus allele, or where the locus may map differently in another population. Since these markers did not show segregation distortion in the KxO population, they are not likely to have been the cause of any problems in map construction. Although it appears quite possible that a few of the flagged framework markers have stretched or extended the previous map, and that these may indeed represent cases where multiple loci have segregated, the general structure of the KxO map has changed little from previous maps (see Additional file 6), so it can be concluded that duplicated DArT loci have had a minimal effect on the current mapping effort.

## Conclusion

The new DArT markers reported in this study have been carefully documented and tested in a variety of ways, and are presented to the international research community along with a complete set of sequence data and supporting files. These markers have been shown to be useful in expanding and refining an existing molecular map in oat, and will provide a solid basis for any new mapping effort. Furthermore, they have proved to be useful in examining the structure of genetic relationships within a diverse set of oat germplasm, and will provide a strong foundation for any type of genetic analysis that requires reasonably-dense whole-genome coverage of genetic polymorphisms. In the future, DArT markers will provide new opportunities for directed breeding of superior oat cultivars, and guidance in the maintenance of genetic diversity. Due to technical considerations, DArT markers will be most useful when they are assayed in parallel across all available loci, and across a moderately large sample of germplasm. Thus, their use will encourage full genome analysis and the publication of complete data sets. As a result, further information will not only be readily available, but will be compatible for use in comparative genomic analysis. Finally, the available sequence data for these markers will contribute to a growing framework of genetic information that is relevant across related grass species and beyond.

## Methods

### Genetic populations and DNA preparation

A panel of 182 accessions of cultivated oat with global representation was assembled through consultation among authors and colleagues (see Additional file 1). Panel entries originated from the countries shown in Table 1. Seed and/or isolated DNA was contributed by authors at most of the organizations participating in this study. A set of 80 lines from the KxO mapping population was also selected for this work, including most of the 71 lines from the original mapping population [8] and a small set of lines from an extended set [9]. The 80 selected lines (see Additional file 5) provided the most complete set of existing marker scores, and had the fewest heterozygous regions. DNA from leaf tissue was purified and prepared by a variety of methods specific to collaborating laboratories. In most cases, DNA was prepared from plants grown from a random bulked sample of seed. All methods yielded DNA samples of quality similar to that obtained using the methods recommended by DArT P/L [27].

### Preparation of DArT arrays

Preliminary tests of various methods for complexity reduction and library representations were performed, and the *Pst*I/*Taq*I method was chosen for the development of DArT clone libraries for oat. Details of this methodology are described elsewhere [23,25] and briefly below. A subset of 60 cultivated lines from the diversity panel was selected for clone development (identified in Additional file 1). For some libraries used to prepare the first array, an additional 14 lines from non-cultivated species of *Avena *were used. These accessions were intended to increase the potential coverage of a portion of the DArT markers for cross-species comparisons (to be reported elsewhere). DNA samples from the above lines were pooled as indicated, with approximately equal concentrations from each entry. Separate libraries of clones were developed for each of three discovery arrays. The first array was developed from a composite of three libraries made from three different pools: the first containing 60 cultivated varieties (4224 clones), the second containing 14 non-cultivated accessions (1536 clones), and the third containing 12 non-cultivated accessions plus 10 cultivated varieties (384 clones). The next two arrays (Array II and Array III), each comprising 6,144 clones, were developed using the same set of 60 cultivated varieties as for the main library used in Array I. The library construction procedure began with the digestion of 20–100 ng of a mixture of DNA samples with two units of *Pst*I and two units of *Taq*I (NEB, Beverly, MA, USA). A *Pst*I adapter (5'-CAC GAT GGA TCC AGT GCA-3' annealed with 5'-CTG GAT CCA TCG TGC A-3') was simultaneously ligated to the digested DNA with T4 DNA ligase (NEB). For Arrays II and III, 2 units of *Ssp*I enzyme were added to the digestion/ligation mix to eliminate the clones containing tandemly repeated sequences, as such clones had affected the performance of Array I. Aliquots of 1 μl of the ligation products were PCR-amplified in 50 μl reactions using the DArT-*Pst*I primer (5'-GAT GGA TCC AGT GCA G-3') under the cycling conditions described by Wenzl *et al*. [25].

A library was prepared from the amplification products as described by Jaccoud *et al*. [23] with modifications [25]. Inserts were amplified from individual clones as in previous work [23]. The amplification reactions were dried at 37°C, washed with 70% ethanol, and dissolved in a new spotting buffer developed specifically for Erie Scientific poly-L-lysine microarray slides (Wenzl *et al*. in preparation). The amplification products were printed onto poly-L-lysine-coated slides (Erie Scientific, Portsmouth, NH, USA) using a MicroGridII arrayer (Biorobotics, Cambridge, UK). After printing, the material on the slides was denatured by incubation in hot water (95°C) for 2 min, then dried by centrifugation.

### Genotyping of individual DNA samples

The genomic representations of single oat accessions were generated with the same complexity reduction method used to prepare the library spotted on the array. The representations were ten-fold concentrated by precipitation with one volume of isopropanol, then denatured at 95°C for 2 min. The samples were labelled using 0.1 μl of cy3- or cy5-labelled dUTP, random decamers (Amersham Biosciences, Castle Hill, NSW, Australia), and the exo^-^Klenow fragment of *Escherichia coli *DNA polymerase I (NEB). Labelled representations (called targets) were added to 50 μl of a 50:5:1 mixture of ExpressHyb buffer (Clontech, Mountain View, CA, USA), 10 g/l herring sperm DNA (Promega, Annandale, NSW, Australia), and a 6-FAM-labelled polylinker fragment of the plasmid that was used for library preparation. The polylinker fragment was used as a reference to determine, for each clone, the amount of DNA spotted on the array [23]. The hybridisation mixtures were denatured, hybridised to microarrays overnight at 65°C, then the slides washed according to [23].

### Image analysis and polymorphism scoring

Slides were scanned using a Tecan LS300 (Grödig, Salzburg, Austria) confocal laser scanner. The TIF images derived from the slide scanning were analysed using DArTsoft version 7.3 (Cayla *et al*. in preparation), a dedicated software package developed at DArT P/L which is available to DArT network members [27]. DArTsoft was used to automatically analyse batches of up to 96 slides to identify and score polymorphic markers. Briefly, the relative hybridisation intensity of each clone on each slide was determined by dividing the hybridisation signal in the target channel (genomic representation) by the hybridisation signal in the reference channel (polylinker). Clones with variable relative hybridisation intensity across slides were subjected to fuzzy k-means clustering to convert relative hybridisation intensities into binary scores (presence *vs*. absence). Clones that did not fit an expected bimodal (two-cluster) distribution were discarded from further analysis. Entries from the diversity panel and from the KxO mapping population were screened separately on the three discovery arrays. Because not all of the diversity entries were available at each stage of screening, and because technical difficulties resulted in some lines being omitted, the actual composition of entries screened on each array was slightly different (see Additional file 2). Standard methods of marker discovery were deployed using a combination of parameters automatically extracted from the array data using the DArTsoft program: (1) marker quality (Q), which measures between-cluster variance as a percentage of total variance in fluorescent signal distribution among tested samples, (2) marker call rate (percentage of effective scores), and (3) Polymorphism Information Content (PIC). The markers reported in this paper were selected with Q >73, call rate >80%, and PIC >0.1.

### DArT clone sequence analysis

The *E. coli *clones containing the polymorphic markers identified using the three discovery arrays were re-arrayed into seven 384-deep-well microtiter plates and grown at 30°C for 22 hrs in Terrific broth. Plasmid DNA, isolated using the Eppendorf PerfectPrep Plasmid 384 procedure, was sequenced in both directions using the M13R (5'-GGAAACAGCTATGACCATG-3') and T7-ZL (5'-TAATACGACTCACTATAGGG-3') primers with the Applied Biosystems Big Dye Cycle sequencing chemistry at the genomics facility at Purdue University [46]. Following an ethanol precipitation cleanup step, the reactions were run on an Applied Biosystems 3730xl capillary electrophoresis instrument. All sequence reads were assembled and merged to provide one high-quality read per clone where possible. Vector sequences and *Pst*I sites were trimmed so as to not introduce biased similarity among DArT clones in current or future analyses.

### Sequence assembly

The merged sequences were assembled using the SeqMan Pro assembly module of Lasergene 7.2.1 (DNASTAR, Inc., Madison, WI). The SeqMan Pro assembler checks sequence similarity in a rolling window of 50 bases to ensure that the similarity requirement is met in all windows. Three different sets of assembly parameters were tested. Parameters for the most stringent assembly (designated A1) included a 25 base match requirement for entry into the assembly, a 95% similarity requirement, a 50 base spacing for matching *mers *(tags used to accelerate assembly), a minimum of 50 bases per sequence, a gap penalty of 0.25, a gap length penalty of 0.5, and a maximum of 15 mismatched end bases. Parameters for a relaxed assembly (A2) were similar to those of assembly A1 except for a minimum 15-base match requirement, a 75% similarity requirement, a gap penalty of 0, and a gap length penalty of 0.25. Parameters for an intermediate assembly (A3) were similar to those of assembly A1 except for an 80% similarity requirement and a maximum of 10 mismatched end bases.

### BLAST searching

All sequences were searched locally using downloaded NCBI databases and a local version of the BLAST similarity program BLAST 2.2.18 (Mar-02-2008) [47]. Translated sequences (six reading frames) were searched using BLASTX against the non-redundant NCBI protein database (nr) as well as the Swiss-Prot [48] division of the same database. Sequences were also searched using BLASTN against the non-redundant nucleotide database (nt) and the complete EST database. All databases were downloaded on May 6^th^, 2008.

### Consensus scores for identical clones

The presence of duplicated markers, confirmed by sequence analysis, provided an opportunity to cross-validate marker scores, estimate the frequency of scoring errors, and combine data from duplicated markers into a more complete data set. When data from two or more duplicated markers were combined, we refer to this as a 'consensus marker', and to the resulting scores as 'consensus scores'. Consensus scores were generated by identifying the most common score (allele type) for each oat variety or progeny across all members of the same contig. A missing value was assigned when non-identical scores were present at equal frequencies. Consensus markers were named by appending the suffix "_c" to the name of the marker that had the most complete data. The percent agreement among markers belonging to common contigs was also determined. This was computed as the frequency of differences in marker scores between two members of the same contig, or as the frequency of differences between a member and the consensus scores for those contigs with greater than two members.

### Marker clusters based on scores

DArT markers were also clustered into bins based on marker scores alone. This was done to compare these clusters with those based on sequence, and also to provide a further method to remove redundant markers for framework mapping and diversity analysis. Clustering was performed using the KxO mapping data, the germplasm diversity data, and a combined data set using a Pascal program written expressly for this purpose (available on request). Diversity data were scored as 1, 0, or missing, while mapping data were scored as missing (0) or 1, 3, 4, or 5 to indicate phase (based on a numerical variation of the Mapmaker [35] convention, where '-' = 0, 'A' = 1, 'H' = 2, 'B' = 3, 'C' = 4, and 'D' = 5). Thus, markers that were in opposite phase in the mapping data were not considered identical. For the combined clustering, the merged data set contained 2199 markers (rows) and 278 genotypes (columns). Rows (markers) in this data matrix were clustered based on a simple algorithm that joined all pairs of markers that differed at fewer than a preset threshold number of informative scores, as long as the pair shared at least 50 informative data points. Groups were formed with this threshold set at 1%, 3%, or 5%. These methods prevented the joining of pairs that did not share any data (i.e., where one was scored only on KxO and the other was scored only on the diversity panel), but allowed the joint consideration of both types of data and the commutative joining of groups.

### Molecular mapping

Due to the large number of markers available for mapping, the relatively small size of the KxO population, the large size of the oat genome, and some cytogenetic abnormalities in the KxO cross [9], it was not feasible to construct a map using a single mapping procedure. To achieve a robust result, two different mapping programs were used: EasyMap (Wenzl, P., unpublished) and JoinMap V.3 [34]. Results from these programs were compared to previous maps generated using an additional two programs (GMendel [36] and Mapmaker [35]). Short descriptions of the algorithms employed by these programs are provided in the next paragraph. The data set used in this work is shown in Additional file 5. The first step involved *de novo *map construction using EasyMap. This step was performed by authors who were not previously familiar with oat linkage maps; thus, it provided a good validation of previous work. The second step involved matching the new map with previous versions constructed using GMendel [36] and Mapmaker [35] based on positions and groupings of the common framework markers. Where one or the other map suggested a merging of linkage groups, or if groupings conflicted, the markers in question were re-tested with JoinMap v.3 [34] using a small data set that included only those markers. If a single group could be formed at LOD 5, or if a group could be formed at a lower LOD that was compatible with aneuploid assignment [49], then the JoinMap version was accepted. All groups were further tested using JoinMap to re-estimate marker order within the group, and the three different map versions were compared using the software C2Maps (an enhancement of M5 [50], available from the corresponding author). Either the JoinMap or the EasyMap version of the ordering was accepted, depending on which was closest to the previously published order of framework markers within a given linkage group.

A brief description of algorithms employed by the four programs is as follows. Mapmaker [35] compares marker orders by maximum likelihood. It performs exhaustive comparisons to build the most probably framework for a subset of markers, then adds and ripples new markers to the framework. Generally, orders selected in Mapmaker had an LOD score greater than 2. The GMendel [36] and JoinMap [34] programs perform mapping by simulated annealing. This method estimates the shortest linear map by simulating different gene orders for groups of loci in a progressive manner and saving only the shortest orders. These programs do not provide a significance test for marker order, but they exclude markers that contribute to unstable marker orders. Prior to selecting orders in the above programs, linkage groups were established at LOD 7, or relaxed to LOD 5 as explained above. The EasyMap program (Wenzl, P., unpublished) provided highly automated procedures for recursively examining and improving a map. An initial order of all makers was established using the RECORD algorithm [51] as if there were only a single linkage group. Next, this linkage group was split into multiple groups at points where the recombination frequency was above a threshold of 30%. This threshold was computed based on a simulation of the assumed genome size and number of available markers. Then, the marker order within each linkage group was recursively optimized while removing markers with potential genotyping errors (LOD>4) based on posterior probabilities. EasyMap was developed to automate procedures that were used previously in the development of a high-density consensus map in barley [41].

### Diversity analysis

Agglomerative clustering using the un-weighted pair group method with averages (UPGMA) was performed using the Agglomerative Nesting (AGNES) function in the R statistical environment [52]. The distance metric used was the Manhattan distance, which represents (in this case) the number of loci for which two lines differed in their marker score. Principle coordinate analysis (PCA) was conducted using DARwin Version 5.0.156 [53].

### Pedigree analysis

Where possible, pedigrees of varieties in the germplasm panel were obtained from the literature or through correspondence with colleagues. The resulting information was incorporated into the online relational database called 'Pedigrees of Oat Lines (POOL)' [43,44]. This database allows querying of extended pedigrees when varieties share common intermediate parents, and provides a convenient keyword-search for names and synonyms of varieties. Once the pedigrees were incorporated into POOL, a complete matrix of co-ancestry coefficients (*K*) among varieties was computed using an updated version of the software package KIN [54]. All varieties, landraces, and intermediate breeding lines in the pedigrees were assumed to be 100% homozygous and homogeneous for the purpose of these computations. Values of *D *were used to construct an UPGMA-based dendrogram using the same methods described for the marker-based diversity analysis (above).

## Abbreviations

AFLP: amplified fragment length polymorphism; Contig: contiguous assembly of matching DNA sequences; DArT: diversity array technology; Groat: an oat kernel without hulls; QTL: quantitative trait locus/loci; RFLP: restriction fragment length polymorphism; RIL: recombinant inbred line(s); SD: standard deviation; SSR: simple sequence repeat; SCAR: sequence characterized amplified region; PIC: polymorphism information content.

## Authors' contributions

NAT led the planning of this study, coordinated a consortium of members that funded and executed the study, conducted all data analyses except where indicated, led manuscript design and preparation, prepared most tables and figures, and wrote first drafts of most sections of the manuscript. AK played a major role in planning this study, supervised all strategic and molecular components of DArT marker development, led all primary stages of data analyses (marker scoring and quality assessment), contributed to interpretation of data analysis, and wrote sections of the manuscript related to DArT development methods and general results of marker analysis. CPW contributed many aspects of data management and analysis, especially the development and interpretation of the new DArT map and the researching of oat pedigrees. KUH developed DArT libraries and performed significant parts of DArT data production. PW performed the initial linkage mapping using EasyMap. HWR and AB contributed to early discussions related to planning this work. AB, JLJ, and CJH contributed in-depth discussion and data analysis related to marker-based clustering. JMA, AK, GS, and NAT coordinated sequencing of the DArT clones. Most of the above, and all additional authors, contributed to the financial support of this work, contributed to the selection and preparation of germplasm, contributed to the discussion of germplasm diversity, and contributed sections of the manuscript related to their areas of specialty. All authors edited and approved the final version of the manuscript.

## Supplementary Material

Additional File 1 **List of varieties.** Inventory of 182 oat varieties and accessions used in DArT marker development and diversity analysis. Click here for file

Additional File 2 **List of markers.** Complete listing of DArT markers and associated clones showing marker clusters (bins) based on scores, map positions, and clone similarity based on sequence assembly. Click here for file

Additional File 3 **DNA Sequences.** FASTA formatted DNA sequence data containing 2670 vector-trimmed sequences corresponding to 2573 unique DArT clones, and 490 consensus sequences from an assembly of the above. Click here for file

Additional File 4 **BLAST identity.** Potential clone identity based on BLAST for non-redundant set of 1774 DArT clone sequences (consensus and singletons). Click here for file

Additional File 5 **Marker mapping data. **Molecular marker data set (in Mapmaker [35] format) including all DArT scores as well as framework marker scores (from [9]) for a set of 80 RIL progenies from the 'Kanota' × 'Ogle' mapping population. Click here for file

Additional File 6 **Map comparison.** Expanded version of 'Kanota' × 'Ogle' 2008 DArT map showing increased density of DArT markers with annotated comparisons to the previous map [9]. Click here for file

Additional File 7 **Framework Molecular Map of KxO.** Framework version of a molecular marker map in Kanota × Ogle with integrated DArT markers. This is a high-resolution version of the cartoon map presented in Figure 4. Click here for file

Additional File 8 **Detailed map placements.** HTML version of the new 'Kanota' × 'Ogle' DArT framework map, listing approximate placement for additional markers, including more recently published markers. Click here for file

Additional File 9 **Marker diversity data.** Non-redundant DArT marker data set for the germplasm diversity study, containing a full set of data from 1295 non-redundant markers and 182 oat varieties, and a nearly orthogonal set with 1295 markers and 134 varieties. Click here for file

Additional File 10 **Cluster analysis of orthogonal varieties.** This is a high-resolution multi-page version of Figure 6. Click here for file

Additional File 11 **Cluster analysis of all varieties.** UPGMA cluster analysis of germplasm diversity based on 182 oat varieties, including those that were not orthogonal across all three discovery arrays. Click here for file

Additional File 12 **Pedigree clusters.** UPGMA cluster analysis of pedigree distances (D) among 182 oat varieties not orthogonal across all three discovery arrays. Click here for file

Additional File 13 **Potentially duplicated markers.** List of 156 DArT markers for which there is some evidence that the marker may map to more than one locus. Three types of evidence are explained in the table legend. Click here for file

Additional File 14 **Clusters with and without potentially duplicated markers.** Analysis of orthogonal diversity data by "Fanny" routine in statistical package 'R'. Fanny finds fuzzy clusters at a given cluster number, 'K'. These analyses were performed with K = 40 on the complete data set (134 × 1295), and, for comparison, on the same data set with potentially duplicated markers removed (see Additional file 13). Click here for file
