# Supplementary material for: New DArT markers for oat provide enhanced map coverage and global germplasm characterization
Source: BMC Genomics. 2009 Jan 21;10:39. doi: 10.1186/1471-2164-10-39 (PMC2661094; doi:10.1186/1471-2164-10-39)
Supplement: Additional File 6 — Map comparison. Expanded version of 'Kanota' × 'Ogle' 2008 DArT map showing increased density of DArT markers with annotated comparisons to the previous map [9]. [file 1471-2164-10-39-S6.pdf]

Supplementary Figure. Kanota x Ogle (KO) 2008 DArT map with annotated comparisons to previous maps. Linkage groups from the new DArT map are shown on the left with interim group names prefixed by 'KOD'. This map was produced by an iterative consensus-building process using methods described in the text. It incorporates framework markers from the Wight et al. (2003) map as well as most new DArT markers that were polymorphic in KO. Fine ordering of DArT markers should be considered arbitrary, and markers at identical positions (rounded to the nearest cM unit) are shown as un-named crosshairs. Maps on the right, with groups prefixed by 'KOW' are from Wight et. Al (2003) unless indicated otherwise (AH = Atlantica x Hirtula). The length of the placement crosshairs on the KOW groups indicates the tendency of a placed marker to stretch the map. No DArT markers are placed on the KOW map. Red lines indicate homology between mapped markers, and blue lines indicate inferred homoeology based on cross-hybridizing probes. Lines are dashed if they connect to a marker shown as a placement cross-hair. Map scales vary, but are consistent within connected comparisons. Annotations are given in blocks of text where appropriate.

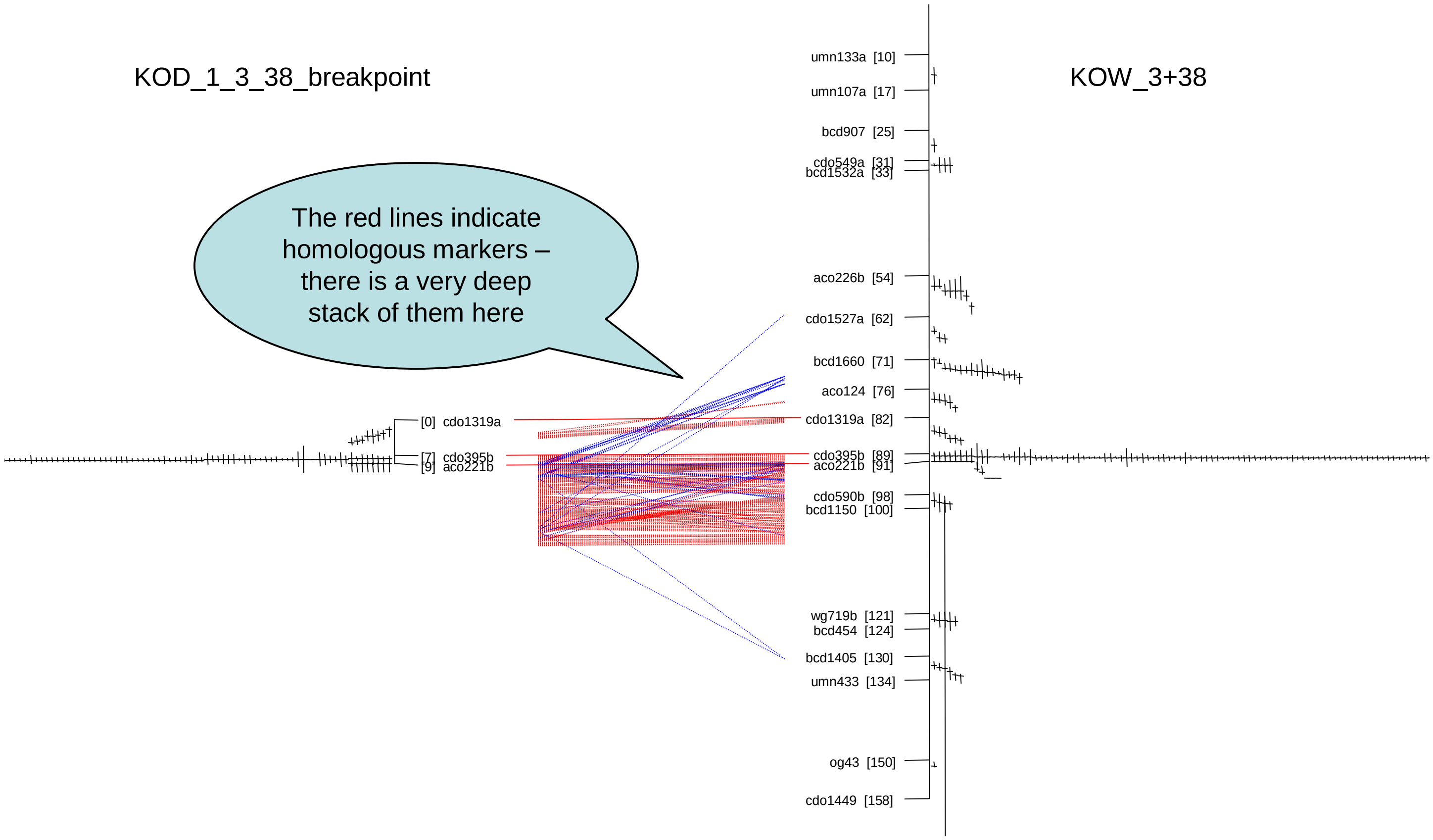

17-7-C Breakpoint: In the new map, we have segregated a dense cluster of markers that we assume are associated with the chromosome 17-7C translocation breakpoint. Placement bars and homology lines are shown for all markers (this fragment only) to indicate that this dense cluster is confined to a short region. Markers not within this cluster are now mapped separately (see KOD…X1-X4) in an attempt to discover whether four chromosome arms associated with a meiotic quadrivalent can be resolved separately. Note that markers within this cluster can be resolved to linear regions of other maps (see discussion).

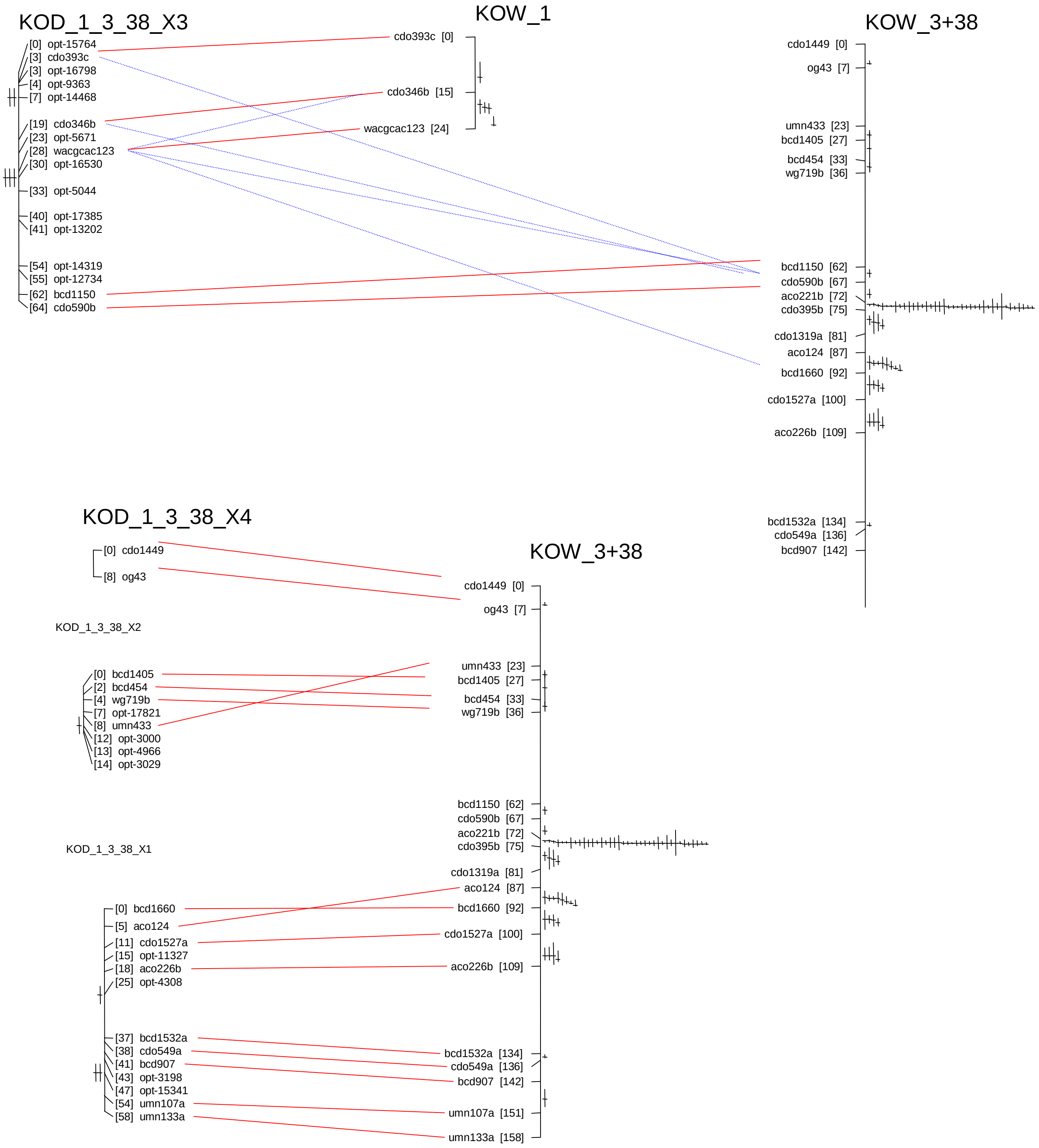

Breaking up the 1_3_38 super group: Grouping in the DArT map brought together KOW group 1 with KOW_3+38, but disrupted the previous order. Speculating that the ordering of these groups is affected by pseudo-linkage of more than two chromosome arms to the translocation breakpoint, we have removed the breakpoint cluster and attempted to order smaller groups. Most of these correspond to the previous KO_3+38 group, allowing KOW_1 to fit with a fragment from near the breakpoint cluster. In the new map, it appears as though fragments X1, X2, X3, and X4 may correspond to 3 or four arms of the quadrivalent. This will need to be confirmed by comparison to new DArT maps.

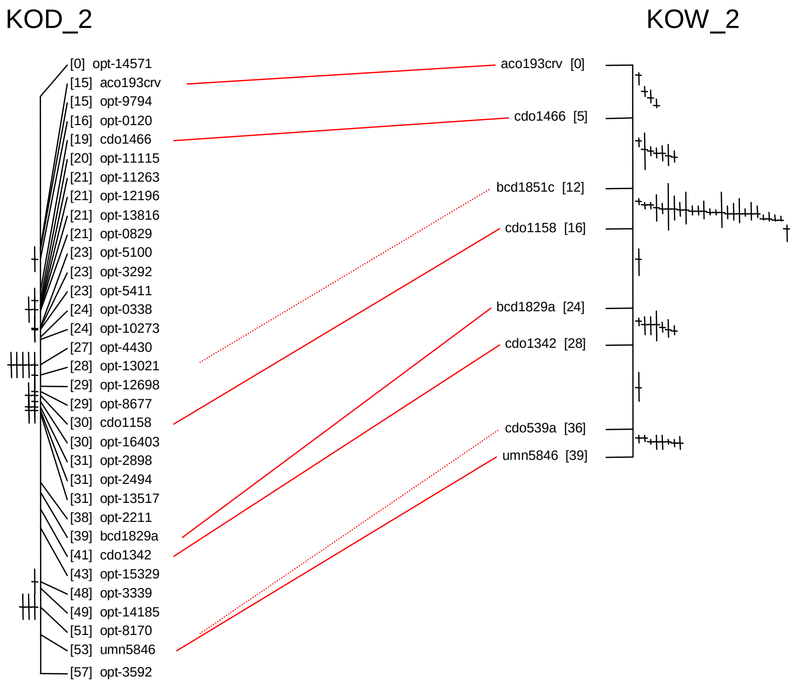

Some stretching of the DArT map occurs relative to the KOW framework map occurs in some places. This is partly due to the attempt to incorporate a greater number of markers. Markers that stretched the KOW framework were systematically removed.

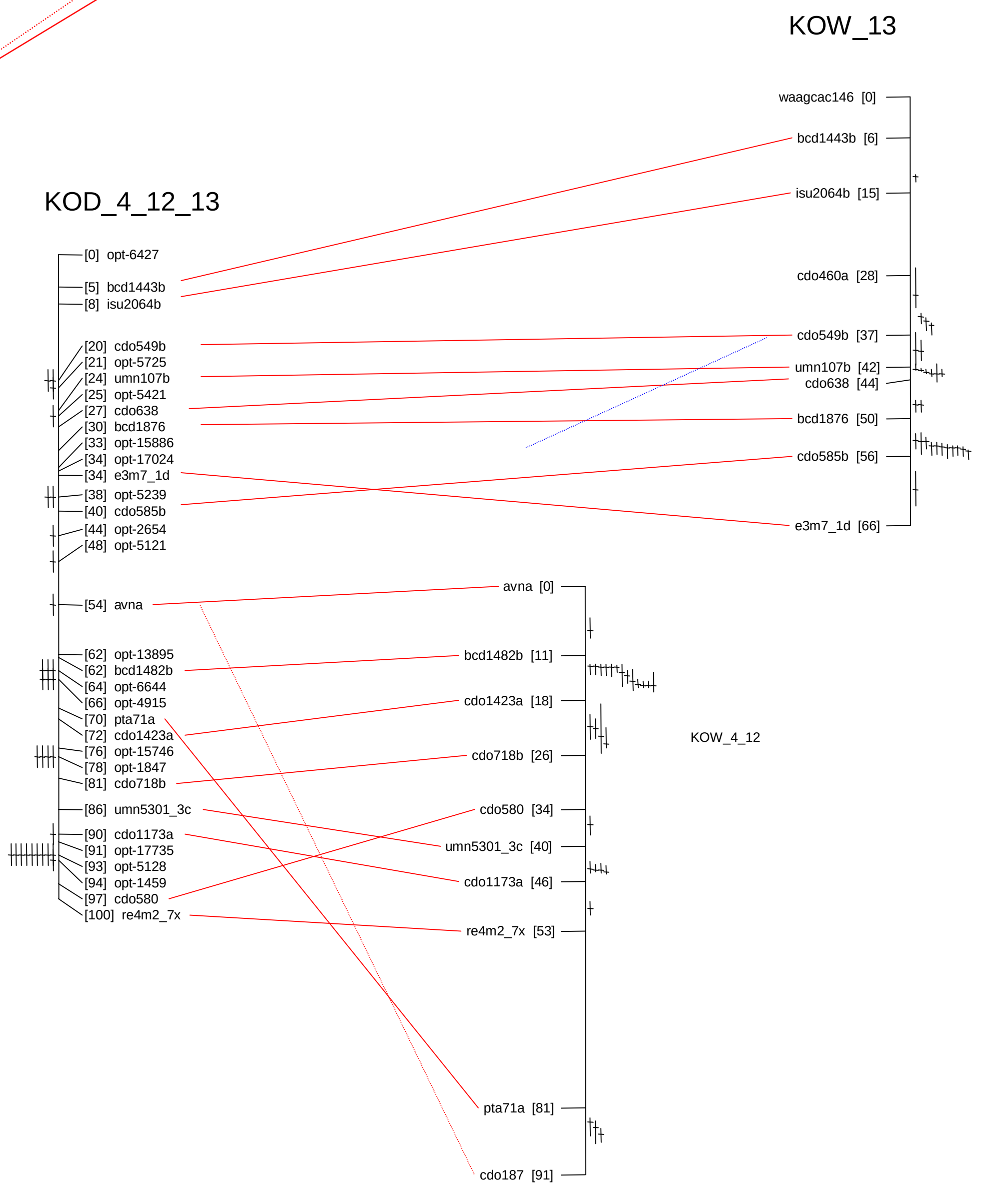

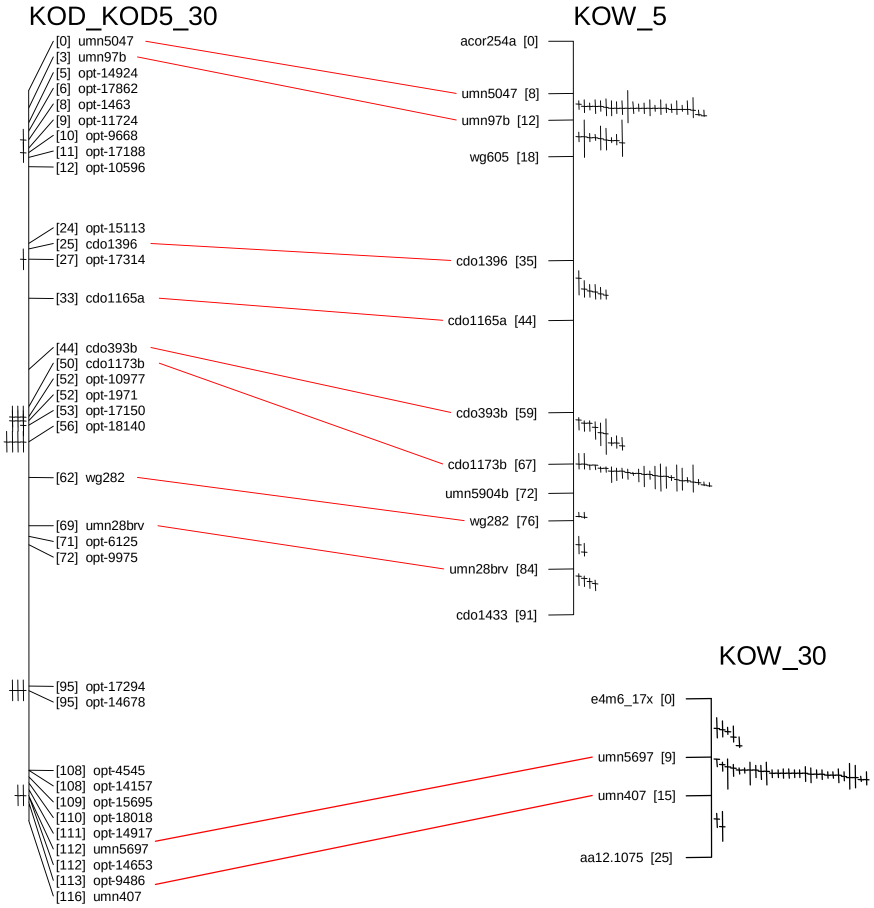
KOD_KOD5_30
[0] umn5047
[3] umn97b
[5] opt-14924
[6] opt-17862
[8] opt-1463
[9] opt-11724
[10] opt-9668
[11] opt-17188
[12] opt-10596
[24] opt-15113
[25] cdo1396
[27] opt-17314
[33] cdo1165a
[44] cdo393b
[50] cdo1173b
[52] opt-10977
[52] opt-1971
[53] opt-17150
[56] opt-18140
[62] wg282
[69] umn28brv
[71] opt-6125
[72] opt-9975
[95] opt-17294
[95] opt-14678
[108] opt-4545
[108] opt-14157
[109] opt-15695
[110] opt-18018
[111] opt-14917
[112] umn5697
[112] opt-14653
[113] opt-9486
[116] umn407
KOW_5
acor254a [0]
umn5047 [8]
umn97b [12]
wg605 [18]
cdo1396 [35]
cdo1165a [44]
cdo393b [59]
cdo1173b [67]
umn5904b [72]
wg282 [76]
umn28brv [84]
cdo1433 [91]
KOW_30
e4m6_17x [0]
umn5697 [9]
umn407 [15]
aa12.1075 [25]

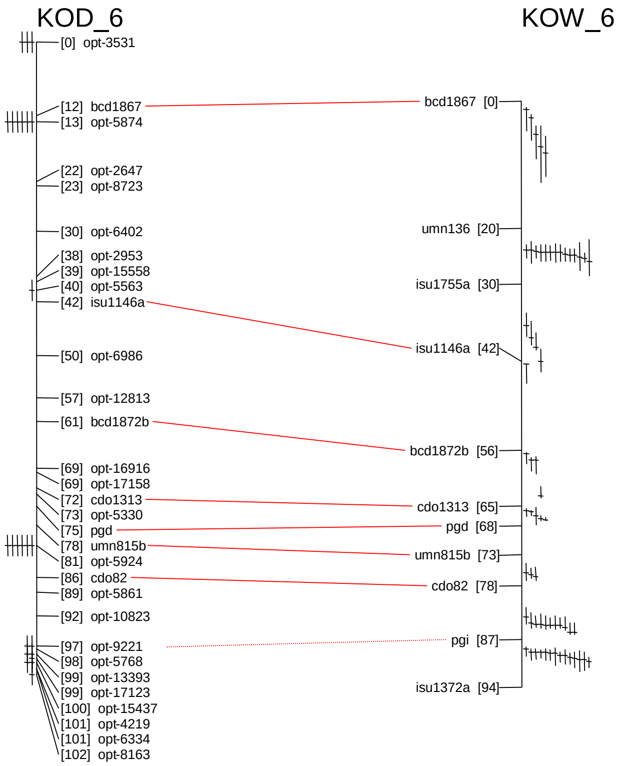
KOD_6
[0] opt-3531
[12] bcd1867
[13] opt-5874
[22] opt-2647
[23] opt-8723
[30] opt-6402
[38] opt-2953
[39] opt-15558
[40] opt-5563
[42] isu1146a
[50] opt-6986
[57] opt-12813
[61] bcd1872b
[69] opt-16916
[69] opt-17158
[72] cdo1313
[73] opt-5330
[75] pgd
[78] umn815b
[81] opt-5924
[86] cdo82
[89] opt-5861
[92] opt-10823
[97] opt-9221
[98] opt-5768
[99] opt-13393
[99] opt-17123
[100] opt-15437
[101] opt-4219
[101] opt-6334
[102] opt-8163
KOW_6
bcd1867 [0]
umn136 [20]
isu1755a [30]
isu1146a [42]
bcd1872b [56]
cdo1313 [65]
pgd [68]
umn815b [73]
cdo82 [78]
pgi [87]
isu1372a [94]

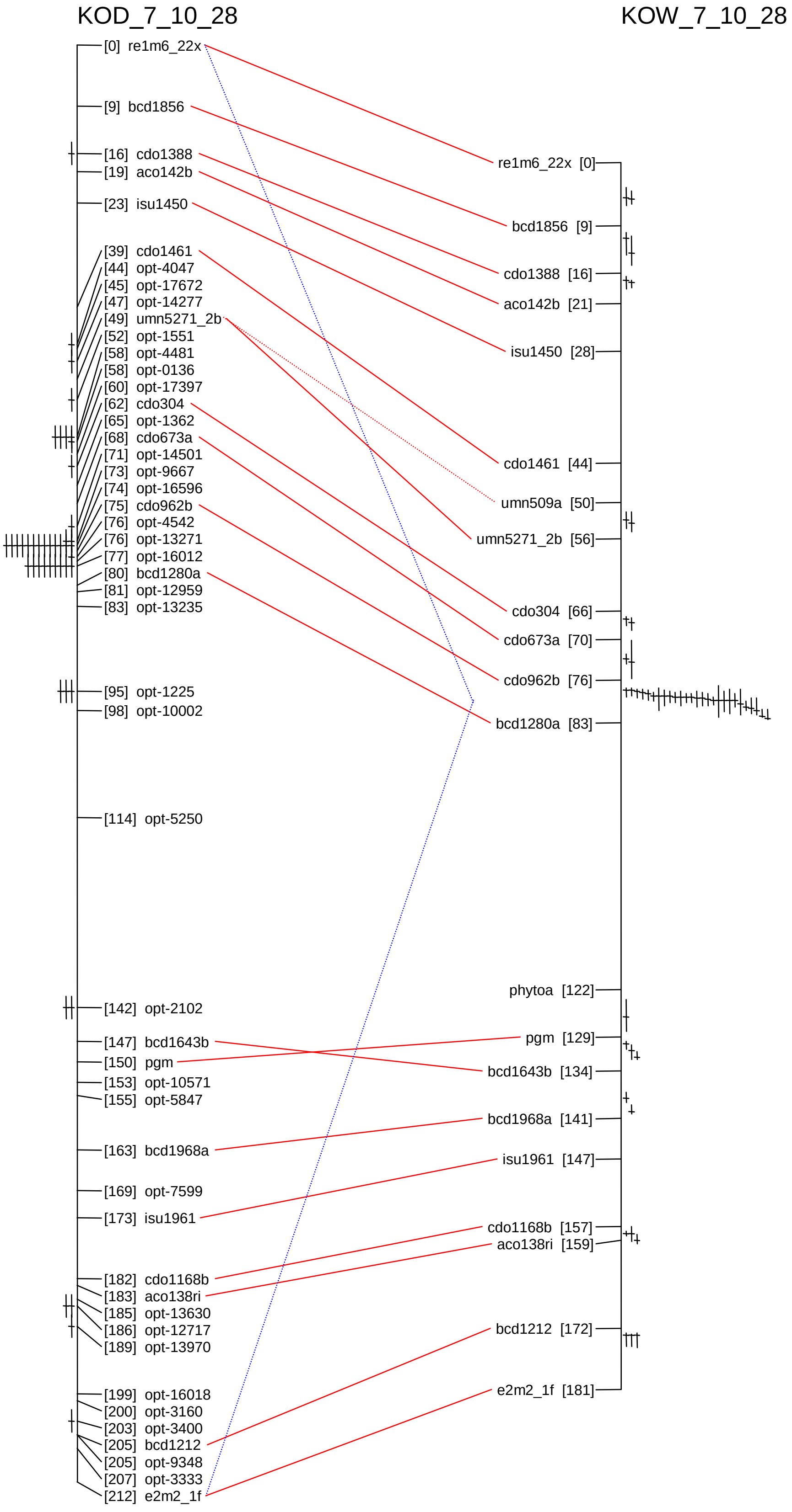

KOD_7_10_28
KOW_7_10_28
[0] re1m6_22x
[9] bcd1856
[16] cdo1388
[19] aco142b
[23] isu1450
[39] cdo1461
[44] opt-4047
[45] opt-17672
[47] opt-14277
[49] umn5271_2b
[52] opt-1551
[58] opt-4481
[58] opt-0136
[60] opt-17397
[62] cdo304
[65] opt-1362
[68] cdo673a
[71] opt-14501
[73] opt-9667
[74] opt-16596
[75] cdo962b
[76] opt-4542
[76] opt-13271
[77] opt-16012
[80] bcd1280a
[81] opt-12959
[83] opt-13235
[95] opt-1225
[98] opt-10002
[114] opt-5250
[142] opt-2102
[147] bcd1643b
[150] pgm
[153] opt-10571
[155] opt-5847
[163] bcd1968a
[169] opt-7599
[173] isu1961
[182] cdo1168b
[183] aco138ri
[185] opt-13630
[186] opt-12717
[189] opt-13970
[199] opt-16018
[200] opt-3160
[203] opt-3400
[205] bcd1212
[205] opt-9348
[207] opt-3333
[212] e2m2_1f
re1m6_22x [0]
bcd1856 [9]
cdo1388 [16]
aco142b [21]
isu1450 [28]
cdo1461 [44]
umn509a [50]
umn5271_2b [56]
cdo304 [66]
cdo673a [70]
cdo962b [76]
bcd1280a [83]
phytoa [122]
pgm [129]
bcd1643b [134]
bcd1968a [141]
isu1961 [147]
cdo1168b [157]
aco138ri [159]
bcd1212 [172]
e2m2_1f [181]

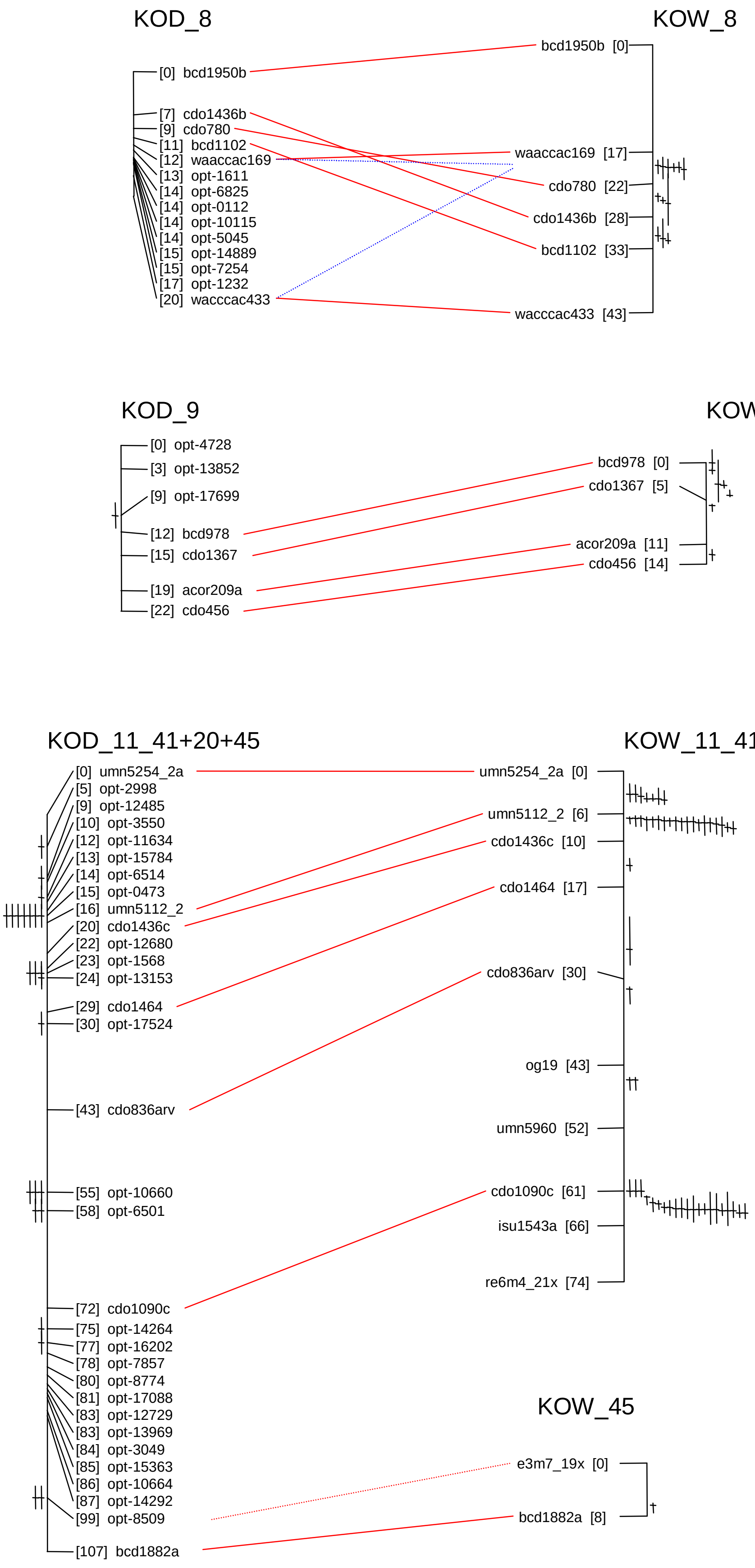
KOD_8
KOW_8
[0] bcd1950b
[7] cdo1436b
[9] cdo780
[11] bcd1102
[12] waaccac169
[13] opt-1611
[14] opt-6825
[14] opt-0112
[14] opt-10115
[14] opt-5045
[15] opt-14889
[15] opt-7254
[17] opt-1232
[20] wacccac433
bcd1950b [0]
waaccac169 [17]
cdo780 [22]
cdo1436b [28]
bcd1102 [33]
wacccac433 [43]
KOD_9
KOW_9
[0] opt-4728
[3] opt-13852
[9] opt-17699
[12] bcd978
[15] cdo1367
[19] acor209a
[22] cdo456
bcd978 [0]
cdo1367 [5]
acor209a [11]
cdo456 [14]
KOD_11_41+20+45
KOW_11_41+20
[0] umn5254_2a
[5] opt-2998
[9] opt-12485
[10] opt-3550
[12] opt-11634
[13] opt-15784
[14] opt-6514
[15] opt-0473
[16] umn5112_2
[20] cdo1436c
[22] opt-12680
[23] opt-1568
[24] opt-13153
[29] cdo1464
[30] opt-17524
[43] cdo836arv
[55] opt-10660
[58] opt-6501
[72] cdo1090c
[75] opt-14264
[77] opt-16202
[78] opt-7857
[80] opt-8774
[81] opt-17088
[83] opt-12729
[83] opt-13969
[84] opt-3049
[85] opt-15363
[86] opt-10664
[87] opt-14292
[99] opt-8509
[107] bcd1882a
umn5254_2a [0]
umn5112_2 [6]
cdo1436c [10]
cdo1464 [17]
cdo836arv [30]
og19 [43]
umn5960 [52]
cdo1090c [61]
isu1543a [66]
re6m4_21x [74]
KOW_45
e3m7_19x [0]
bcd1882a [8]

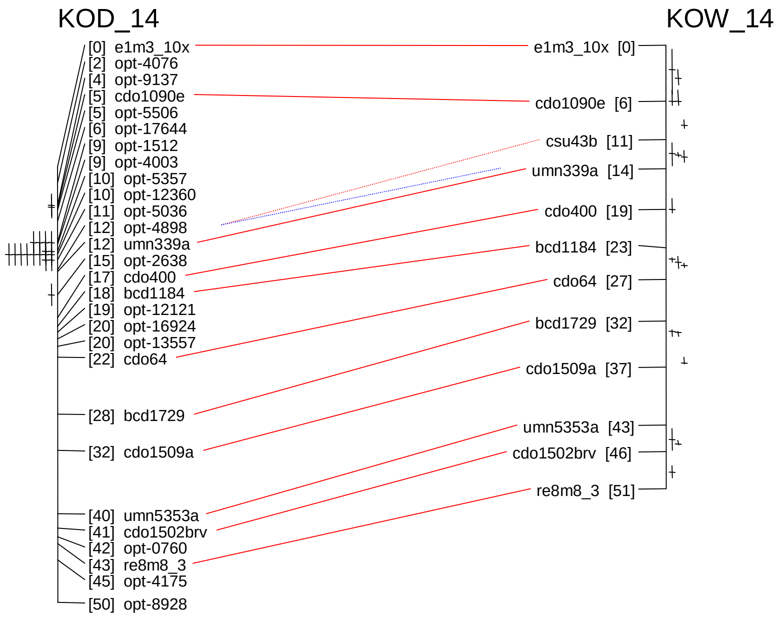
KOD_14
KOW_14
[0] e1m3_10x
[2] opt-4076
[4] opt-9137
[5] cdo1090e
[5] opt-5506
[6] opt-17644
[9] opt-1512
[9] opt-4003
[10] opt-5357
[10] opt-12360
[11] opt-5036
[12] opt-4898
[12] umn339a
[15] opt-2638
[17] cdo400
[18] bcd1184
[19] opt-12121
[20] opt-16924
[20] opt-13557
[22] cdo64
[28] bcd1729
[32] cdo1509a
[40] umn5353a
[41] cdo1502brv
[42] opt-0760
[43] re8m8_3
[45] opt-4175
[50] opt-8928
e1m3_10x [0]
cdo1090e [6]
csu43b [11]
umn339a [14]
cdo400 [19]
bcd1184 [23]
cdo64 [27]
bcd1729 [32]
cdo1509a [37]
umn5353a [43]
cdo1502brv [46]
re8m8_3 [51]

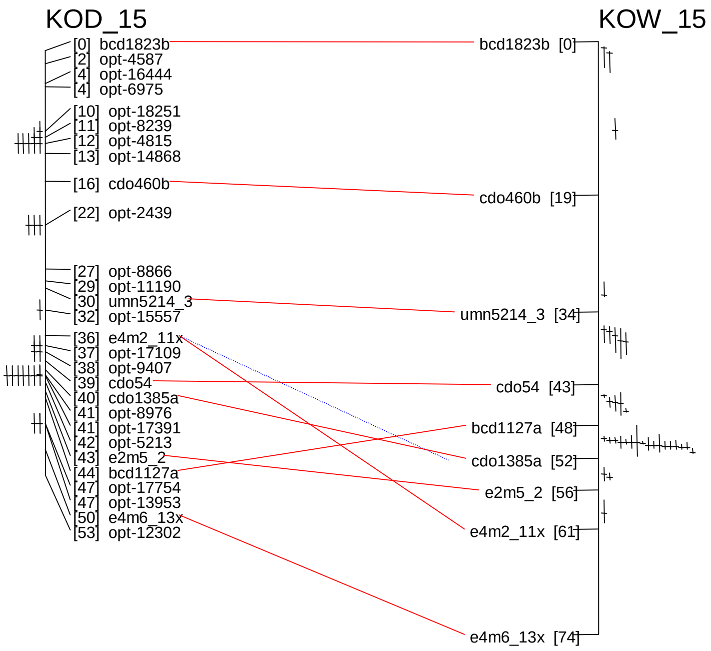
KOD_15
KOW_15
[0] bcd1823b
[2] opt-4587
[4] opt-16444
[4] opt-6975
[10] opt-18251
[11] opt-8239
[12] opt-4815
[13] opt-14868
[16] cdo460b
[22] opt-2439
[27] opt-8866
[29] opt-11190
[30] umn5214_3
[32] opt-15557
[36] e4m2_11x
[37] opt-17109
[38] opt-9407
[39] cdo54
[40] cdo1385a
[41] opt-8976
[41] opt-17391
[42] opt-5213
[43] e2m5_2
[44] bcd1127a
[47] opt-17754
[47] opt-13953
[50] e4m6_13x
[53] opt-12302
bcd1823b [0]
cdo460b [19]
umn5214_3 [34]
cdo54 [43]
bcd1127a [48]
cdo1385a [52]
e2m5_2 [56]
e4m2_11x [61]
e4m6_13x [74]

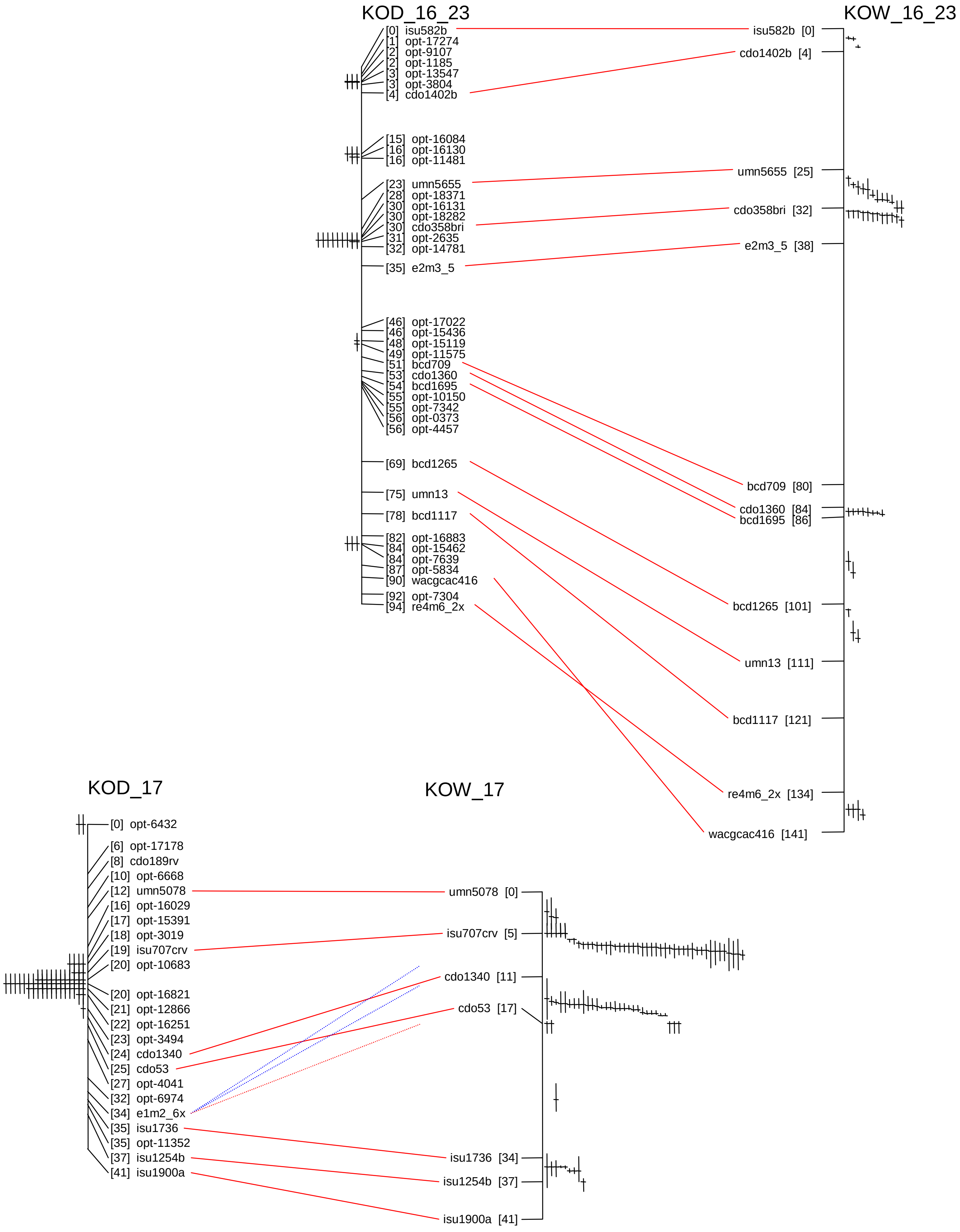
KOD_16_23
KOW_16_23
[0] isu582b
[1] opt-17274
[2] opt-9107
[2] opt-1185
[3] opt-13547
[3] opt-3804
[4] cdo1402b
[15] opt-16084
[16] opt-16130
[16] opt-11481
[23] umn5655
[28] opt-18371
[30] opt-16131
[30] opt-18282
[30] cdo358bri
[31] opt-2635
[32] opt-14781
[35] e2m3_5
[46] opt-17022
[46] opt-15436
[48] opt-15119
[49] opt-11575
[51] bcd709
[53] cdo1360
[54] bcd1695
[55] opt-10150
[55] opt-7342
[56] opt-0373
[56] opt-4457
[69] bcd1265
[75] umn13
[78] bcd1117
[82] opt-16883
[84] opt-15462
[84] opt-7639
[87] opt-5834
[90] wacgcac416
[92] opt-7304
[94] re4m6_2x
isu582b [0]
cdo1402b [4]
umn5655 [25]
cdo358bri [32]
e2m3_5 [38]
bcd709 [80]
cdo1360 [84]
bcd1695 [86]
bcd1265 [101]
umn13 [111]
bcd1117 [121]
re4m6_2x [134]
wacgcac416 [141]
KOD_17
KOW_17
[0] opt-6432
[6] opt-17178
[8] cdo189rv
[10] opt-6668
[12] umn5078
[16] opt-16029
[17] opt-15391
[18] opt-3019
[19] isu707crv
[20] opt-10683
[20] opt-16821
[21] opt-12866
[22] opt-16251
[23] opt-3494
[24] cdo1340
[25] cdo53
[27] opt-4041
[32] opt-6974
[34] e1m2_6x
[35] isu1736
[35] opt-11352
[37] isu1254b
[41] isu1900a
umn5078 [0]
isu707crv [5]
cdo1340 [11]
cdo53 [17]
isu1736 [34]
isu1254b [37]
isu1900a [41]

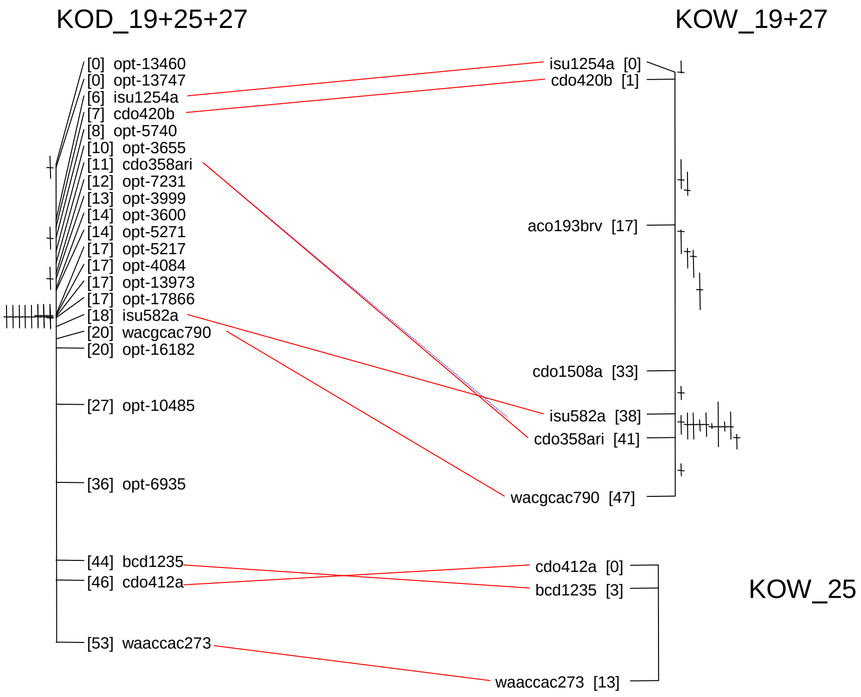
KOD_19+25+27
KOW_19+27
[0] opt-13460
[0] opt-13747
[6] isu1254a
[7] cdo420b
[8] opt-5740
[10] opt-3655
[11] cdo358ari
[12] opt-7231
[13] opt-3999
[14] opt-3600
[14] opt-5271
[17] opt-5217
[17] opt-4084
[17] opt-13973
[17] opt-17866
[18] isu582a
[20] wacgcac790
[20] opt-16182
[27] opt-10485
[36] opt-6935
[44] bcd1235
[46] cdo412a
[53] waaccac273
isu1254a [0]
cdo420b [1]
aco193brv [17]
cdo1508a [33]
isu582a [38]
cdo358ari [41]
wacgcac790 [47]
cdo412a [0]
bcd1235 [3]
waaccac273 [13]
KOW_25

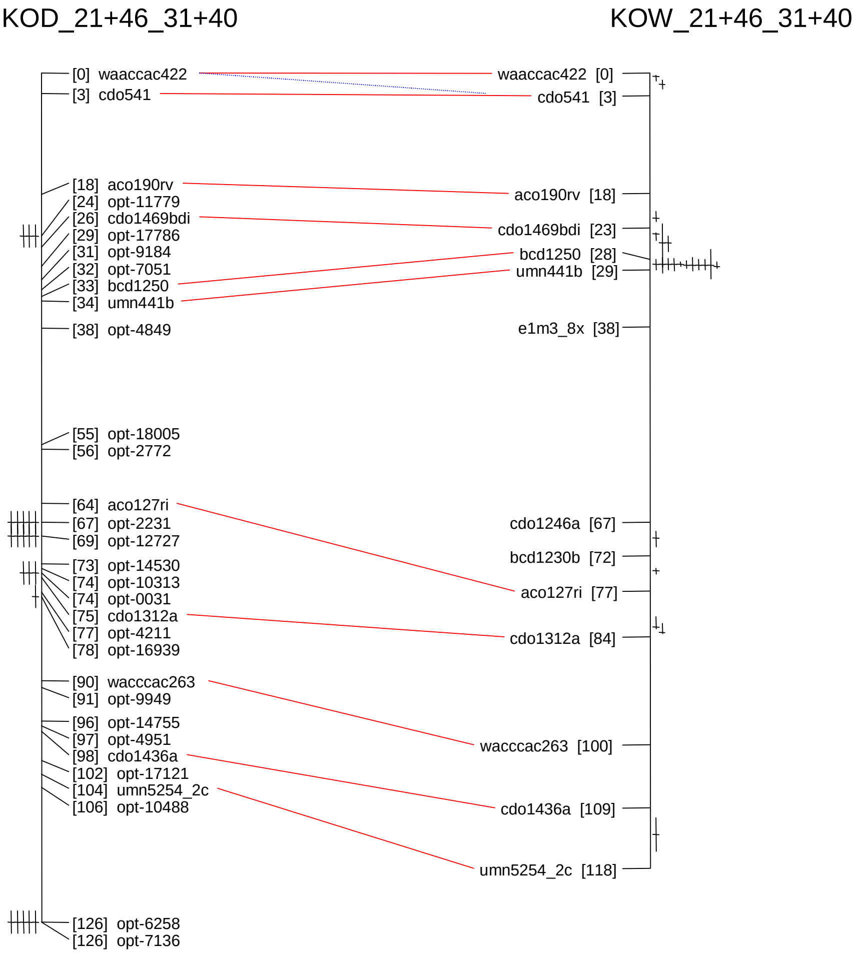
KOD_21+46_31+40
KOW_21+46_31+40
[0] waaccac422
[3] cdo541
[18] aco190rv
[24] opt-11779
[26] cdo1469bdi
[29] opt-17786
[31] opt-9184
[32] opt-7051
[33] bcd1250
[34] umn441b
[38] opt-4849
[55] opt-18005
[56] opt-2772
[64] aco127ri
[67] opt-2231
[69] opt-12727
[73] opt-14530
[74] opt-10313
[74] opt-0031
[75] cdo1312a
[77] opt-4211
[78] opt-16939
[90] wacccac263
[91] opt-9949
[96] opt-14755
[97] opt-4951
[98] cdo1436a
[102] opt-17121
[104] umn5254_2c
[106] opt-10488
[126] opt-6258
[126] opt-7136
waaccac422 [0]
cdo541 [3]
aco190rv [18]
cdo1469bdi [23]
bcd1250 [28]
umn441b [29]
e1m3_8x [38]
cdo1246a [67]
bcd1230b [72]
aco127ri [77]
cdo1312a [84]
wacccac263 [100]
cdo1436a [109]
umn5254_2c [118]

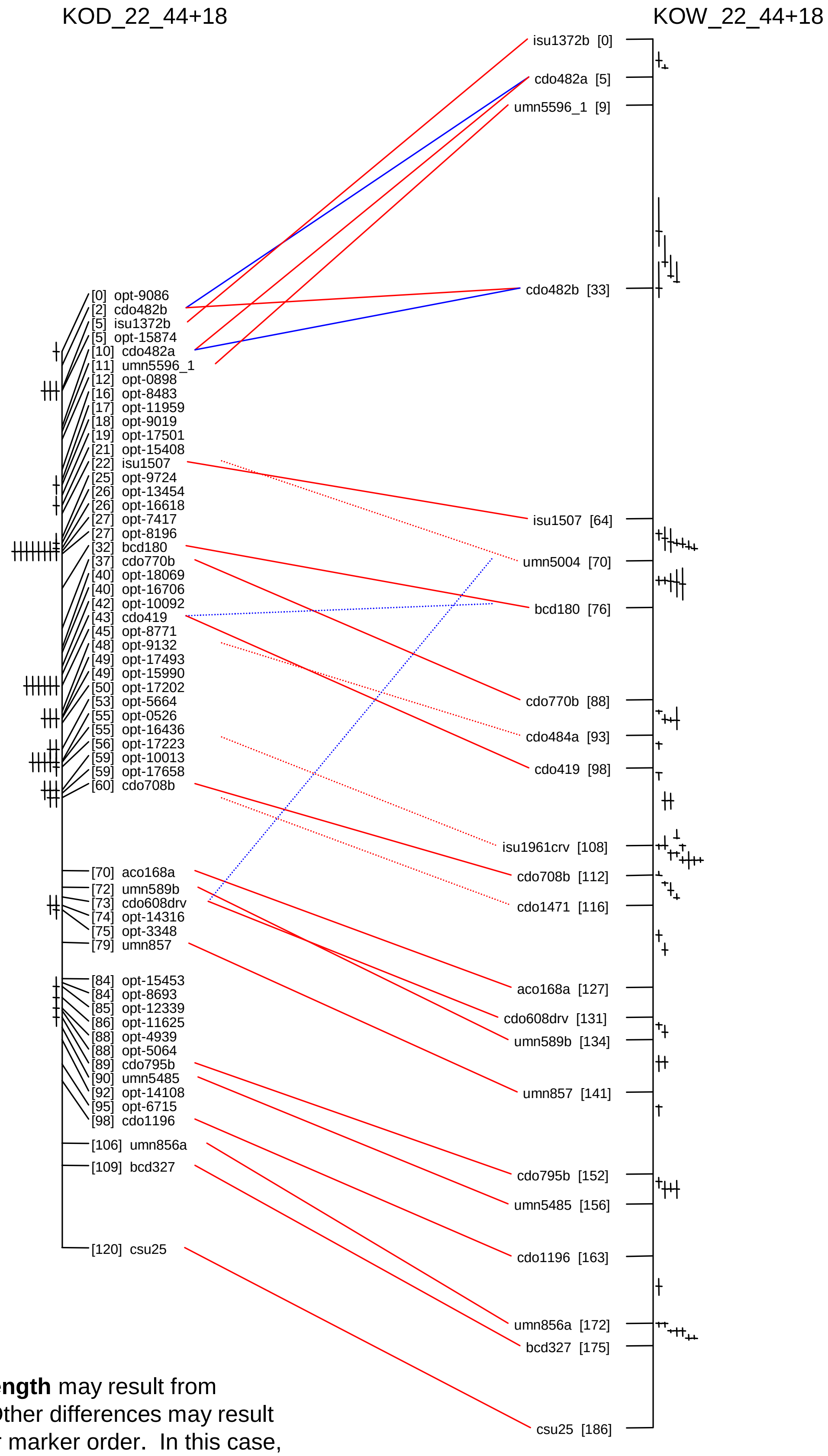

Some differences in map length may result from excluding certain markers. Other differences may result from the discovery of a better marker order. In this case, the reduced length of linkage group 22_44+18 has probably resulted from a combination of these factors.

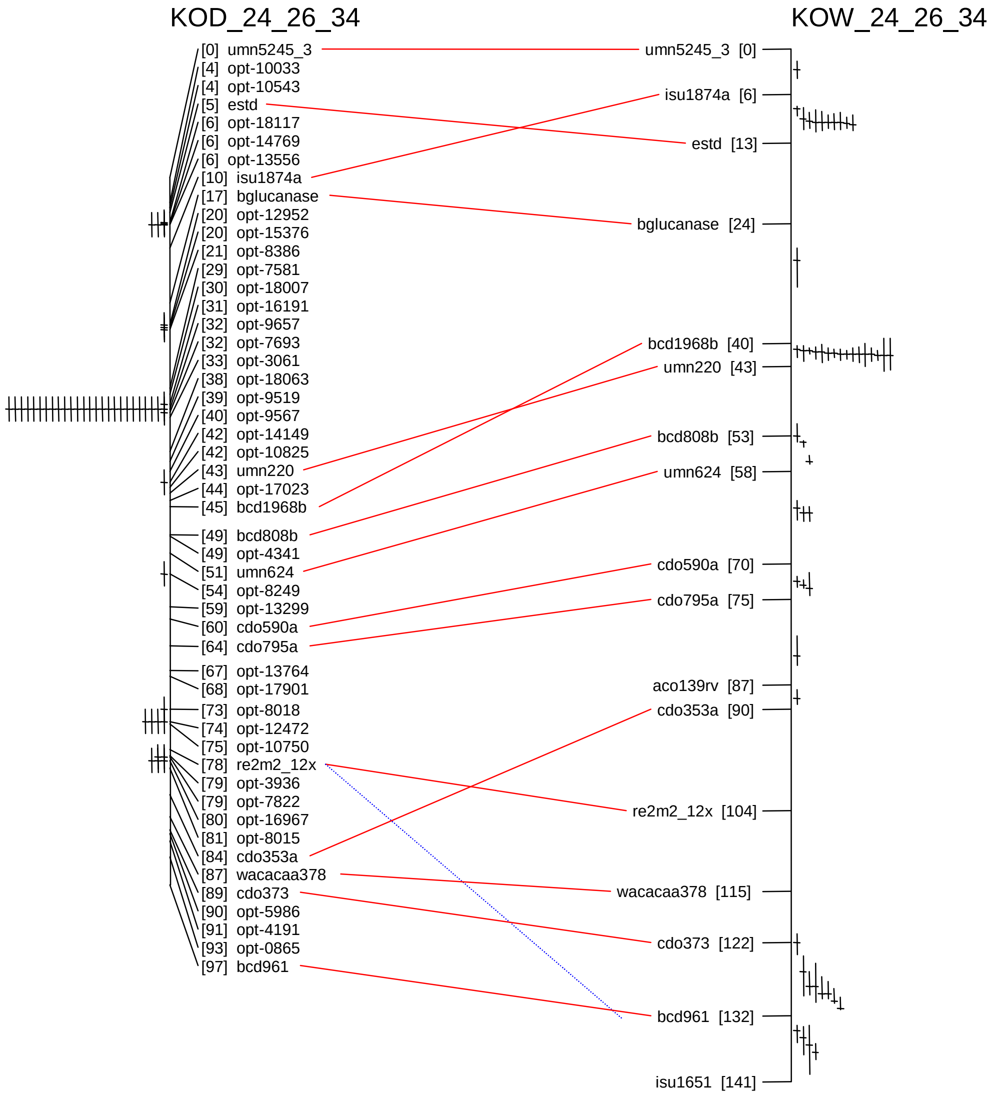
KOD_24_26_34
KOW_24_26_34
[0] umn5245_3
[4] opt-10033
[4] opt-10543
[5] estd
[6] opt-18117
[6] opt-14769
[6] opt-13556
[10] isu1874a
[17] bglucanase
[20] opt-12952
[20] opt-15376
[21] opt-8386
[29] opt-7581
[30] opt-18007
[31] opt-16191
[32] opt-9657
[32] opt-7693
[33] opt-3061
[38] opt-18063
[39] opt-9519
[40] opt-9567
[42] opt-14149
[42] opt-10825
[43] umn220
[44] opt-17023
[45] bcd1968b
[49] bcd808b
[49] opt-4341
[51] umn624
[54] opt-8249
[59] opt-13299
[60] cdo590a
[64] cdo795a
[67] opt-13764
[68] opt-17901
[73] opt-8018
[74] opt-12472
[75] opt-10750
[78] re2m2_12x
[79] opt-3936
[79] opt-7822
[80] opt-16967
[81] opt-8015
[84] cdo353a
[87] wacacaa378
[89] cdo373
[90] opt-5986
[91] opt-4191
[93] opt-0865
[97] bcd961
umn5245_3 [0]
isu1874a [6]
estd [13]
bglucanase [24]
bcd1968b [40]
umn220 [43]
bcd808b [53]
umn624 [58]
cdo590a [70]
cdo795a [75]
aco139rv [87]
cdo353a [90]
re2m2_12x [104]
wacacaa378 [115]
cdo373 [122]
bcd961 [132]
isu1651 [141]

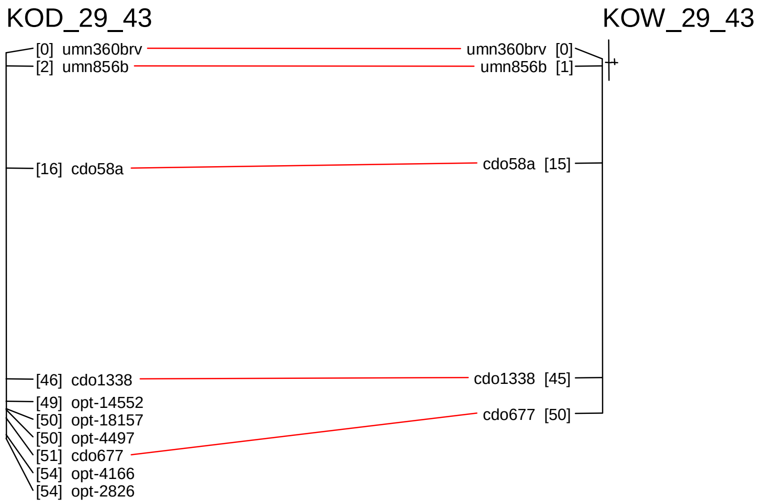
KOD_29_43
KOW_29_43
[0] umn360brv
[2] umn856b
[16] cdo58a
[46] cdo1338
[49] opt-14552
[50] opt-18157
[50] opt-4497
[51] cdo677
[54] opt-4166
[54] opt-2826
umn360brv [0]
umn856b [1]
cdo58a [15]
cdo1338 [45]
cdo677 [50]

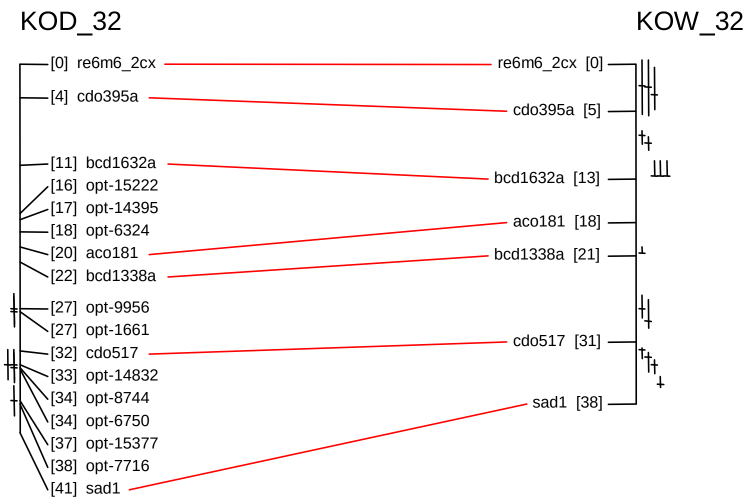
KOD_32
KOW_32
[0] re6m6_2cx
[4] cdo395a
[11] bcd1632a
[16] opt-15222
[17] opt-14395
[18] opt-6324
[20] aco181
[22] bcd1338a
[27] opt-9956
[27] opt-1661
[32] cdo517
[33] opt-14832
[34] opt-8744
[34] opt-6750
[37] opt-15377
[38] opt-7716
[41] sad1
re6m6_2cx [0]
cdo395a [5]
bcd1632a [13]
aco181 [18]
bcd1338a [21]
cdo517 [31]
sad1 [38]

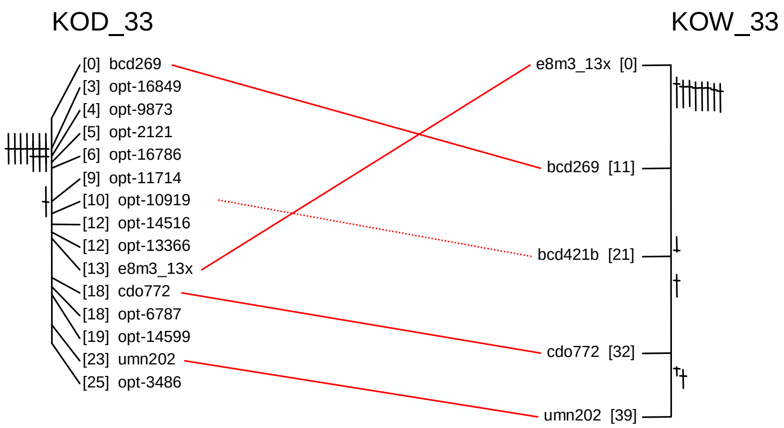
KOD_33
KOW_33
[0] bcd269
[3] opt-16849
[4] opt-9873
[5] opt-2121
[6] opt-16786
[9] opt-11714
[10] opt-10919
[12] opt-14516
[12] opt-13366
[13] e8m3_13x
[18] cdo772
[18] opt-6787
[19] opt-14599
[23] umn202
[25] opt-3486
e8m3_13x [0]
bcd269 [11]
bcd421b [21]
cdo772 [32]
umn202 [39]

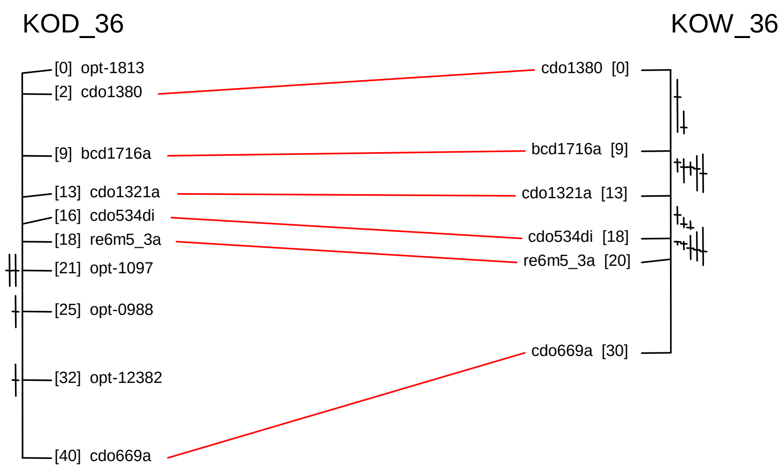
KOD_36
KOW_36
[0] opt-1813
[2] cdo1380
[9] bcd1716a
[13] cdo1321a
[16] cdo534di
[18] re6m5_3a
[21] opt-1097
[25] opt-0988
[32] opt-12382
[40] cdo669a
cdo1380 [0]
bcd1716a [9]
cdo1321a [13]
cdo534di [18]
re6m5_3a [20]
cdo669a [30]

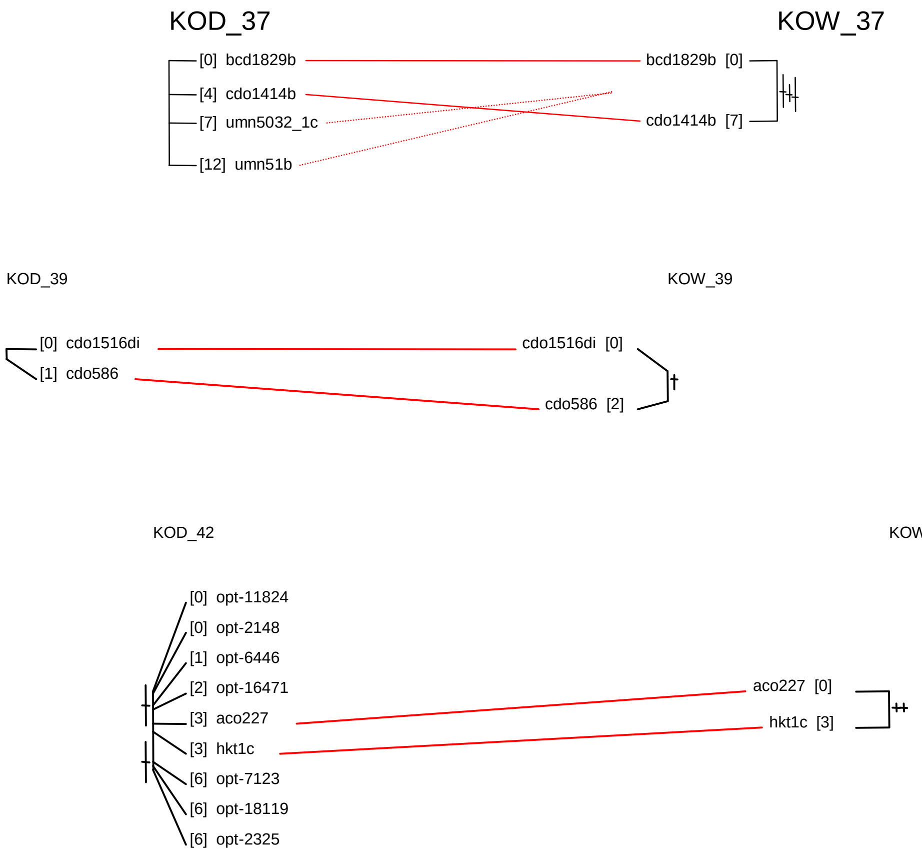

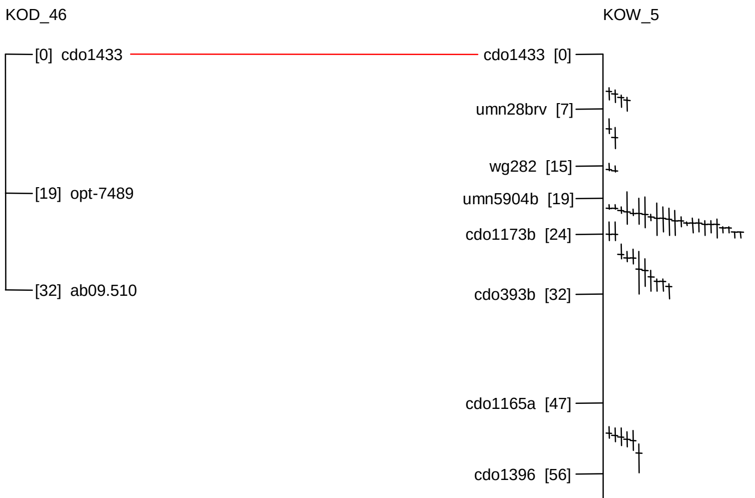

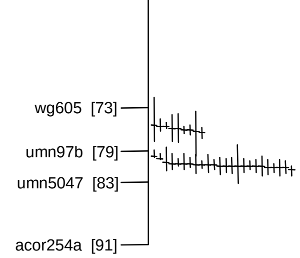

Marker CDO1433 linked to two new DArT markers, but this linkage made it impossible to order these markers with the remainder of linkage group 5 when group 30 was joined in the new map (see figure for KOD_5_30).

KOD_47

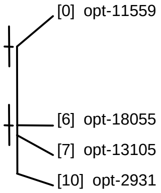

KOD_48

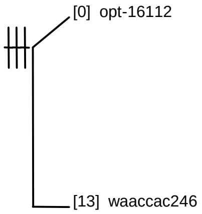

KOD_49

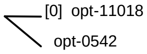

KOD_50

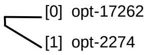

KOD_51

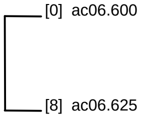

KOD_52

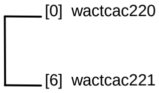

The new orphans: these groups are formed from markers that were not previously joined in the old map, or new DArT markers that do not link to pre-existing groups. They might represent satellite groups from locations near telomeres, where recombination is frequent.
