# Supplementary figures and images for: New DArT markers for oat provide enhanced map coverage and global germplasm characterization
Source: BMC Genomics. 2009 Jan 21;10:39. doi: 10.1186/1471-2164-10-39 (PMC2661094; doi:10.1186/1471-2164-10-39)

# 1\_3\_38\_break

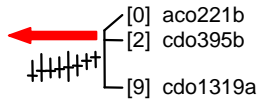

# 1\_3\_38\_X1

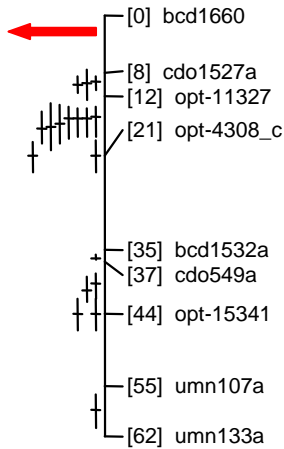

# 1\_3\_38\_X2

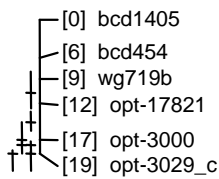

# 1\_3\_38\_X3

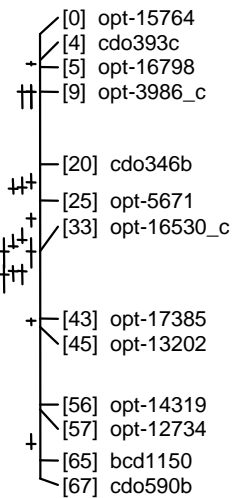

# 1\_3\_38\_X4

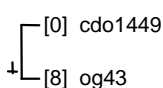

# 2

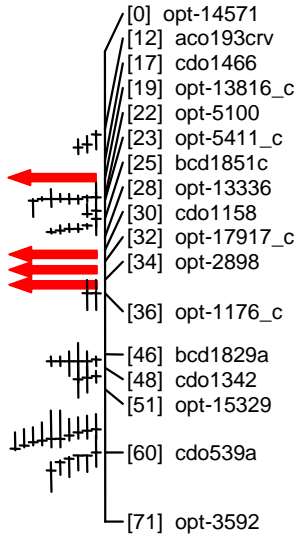

# 4\_12\_13

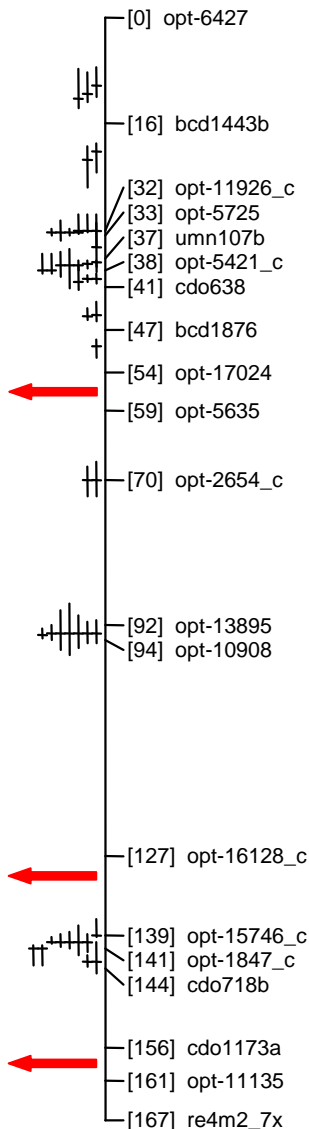

# 5\_30

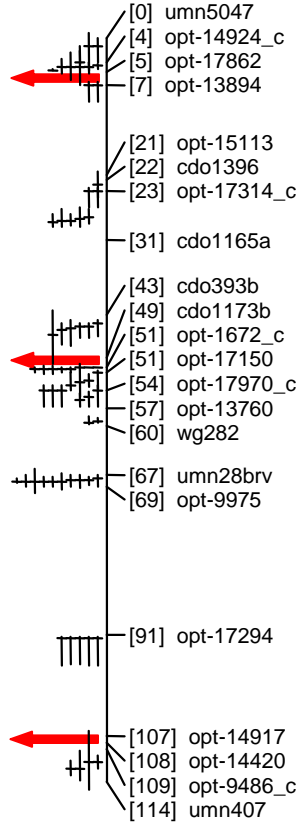

# 6

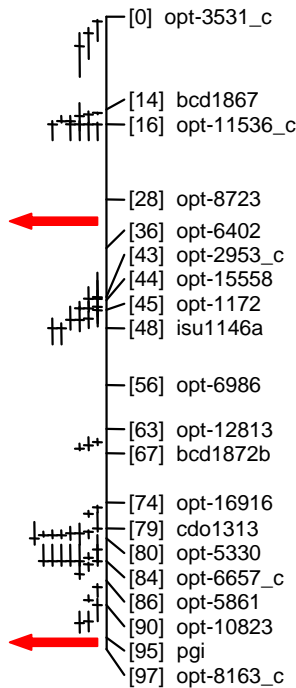

# 7\_10\_28

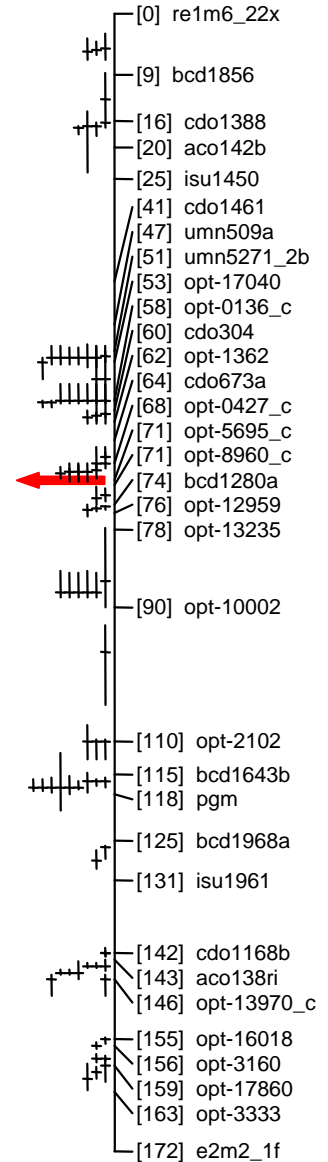

# 8

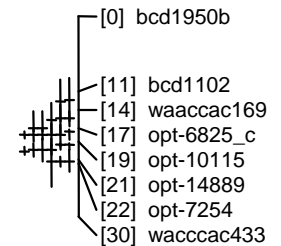

# 9

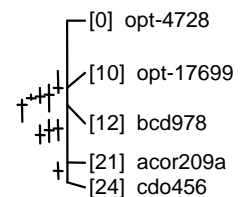

11\_41\_20\_45

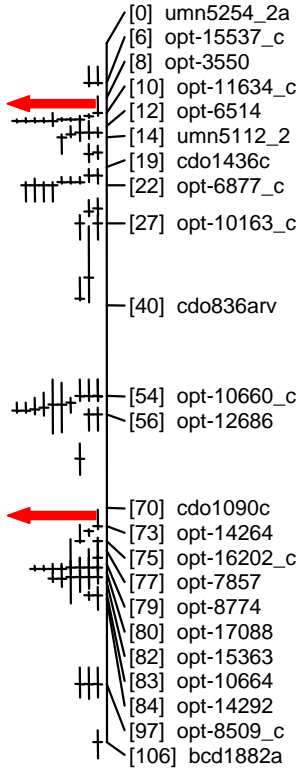

14

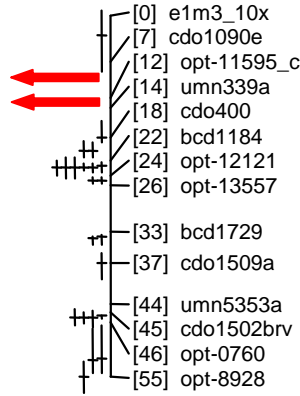

19\_25\_27

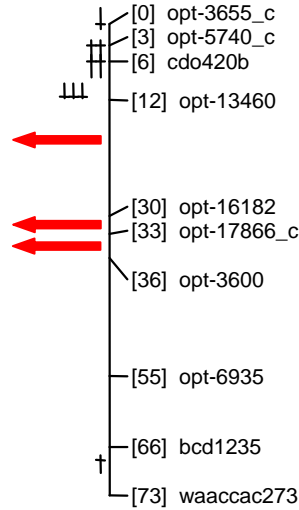

21\_46\_31\_40

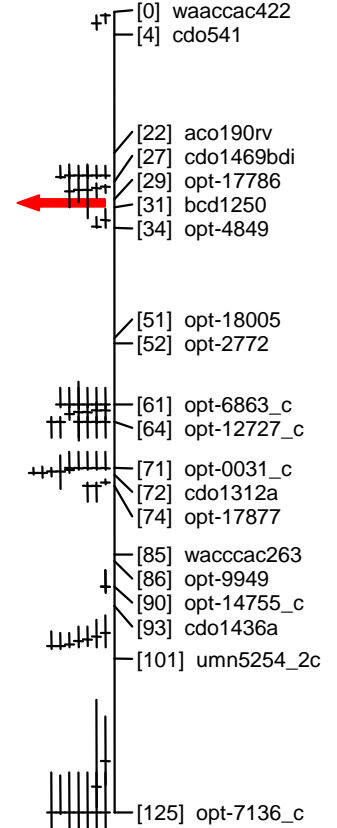

15

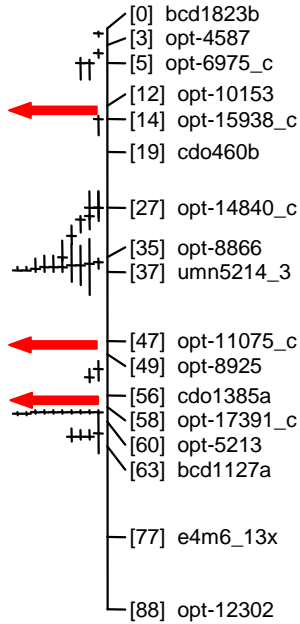

22\_44\_18

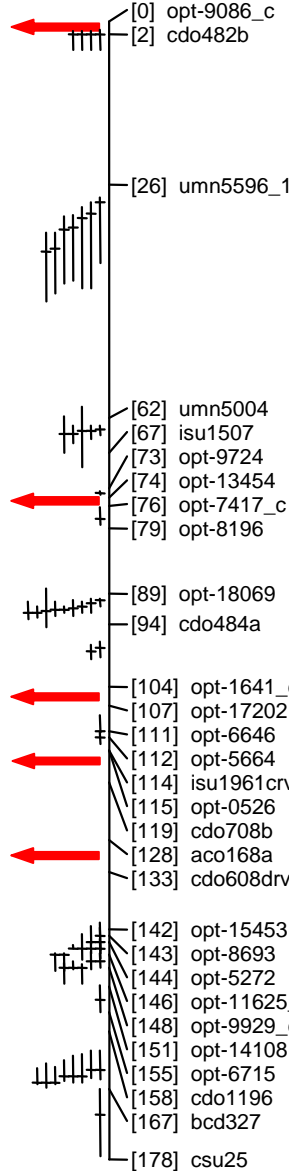

16\_23

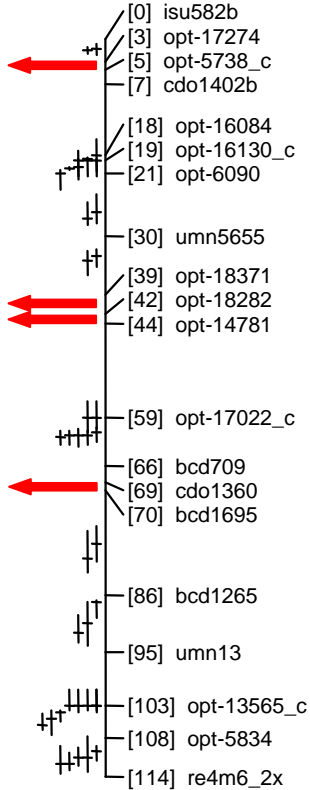

17

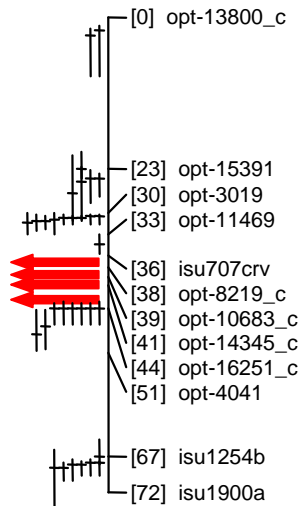

24\_26\_34

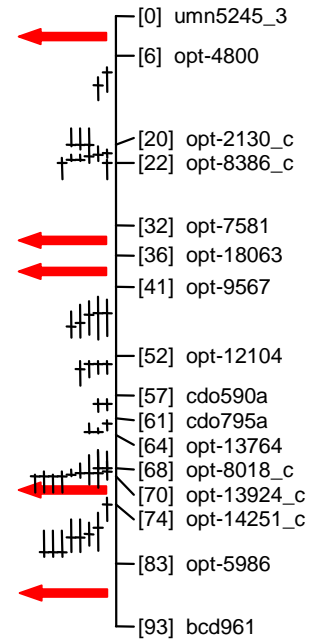

**29\_43**

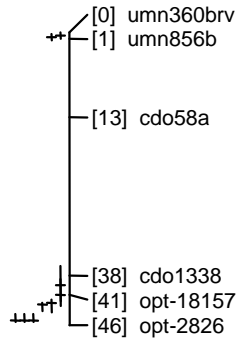

**32**

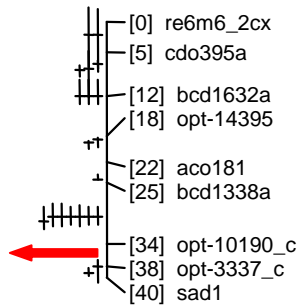

**33**

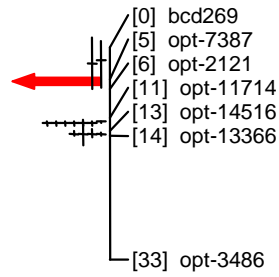

**36**

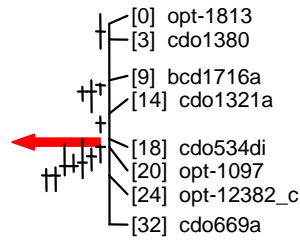

**37**

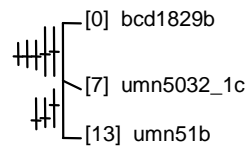

**39**

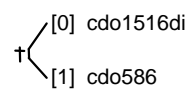

**42**

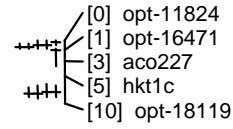

**46**

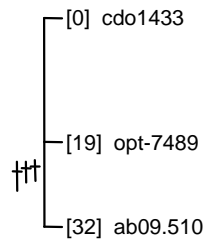

**47**

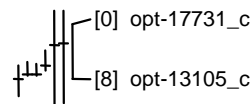

**48**

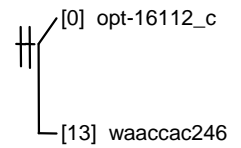

**50**

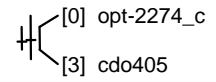

**51**

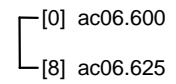

**52**

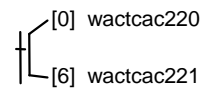

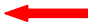 = cluster of  
>10 markers

Supplement: Additional File 7 — Framework Molecular Map of KxO. Framework version of a molecular marker map in Kanota × Ogle with integrated DArT markers. This is a high-resolution version of the cartoon map presented in Figure 4. [file 1471-2164-10-39-S7.pdf]

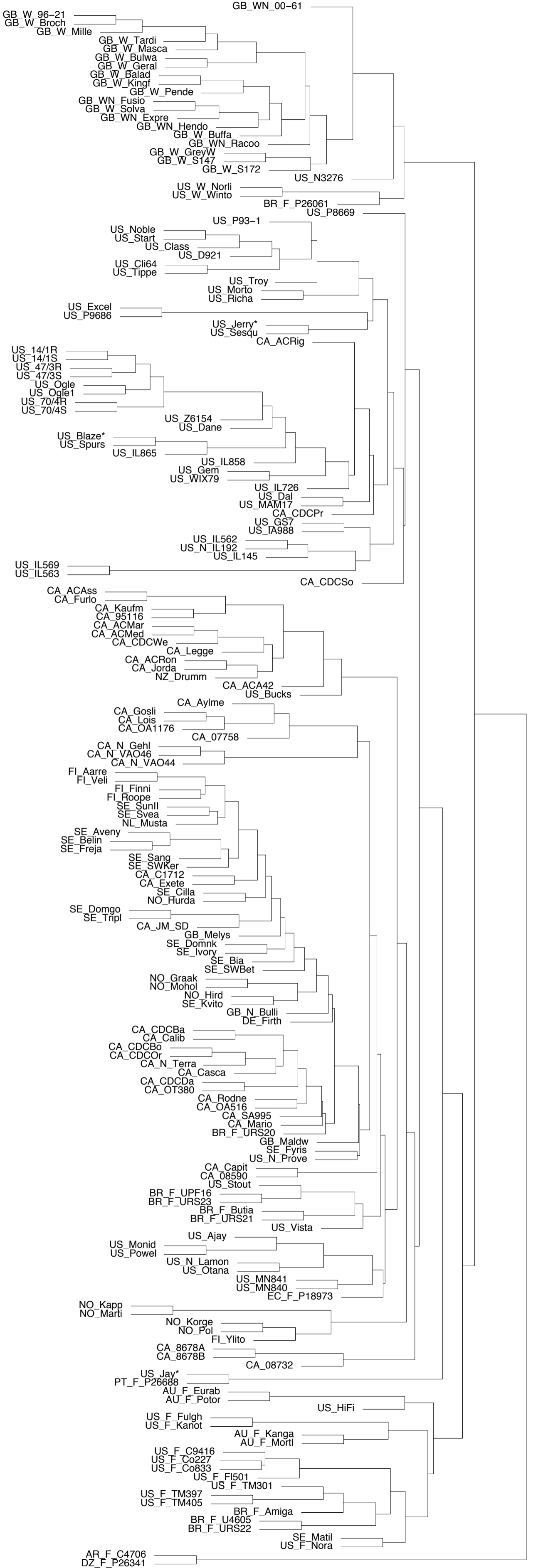

Average Dissimilarity

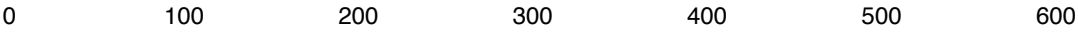

Supplement: Additional File 10 — Cluster analysis of orthogonal varieties. This is a high-resolution multi-page version of Figure 6. [file 1471-2164-10-39-S10.pdf]

Average Dissimilarity

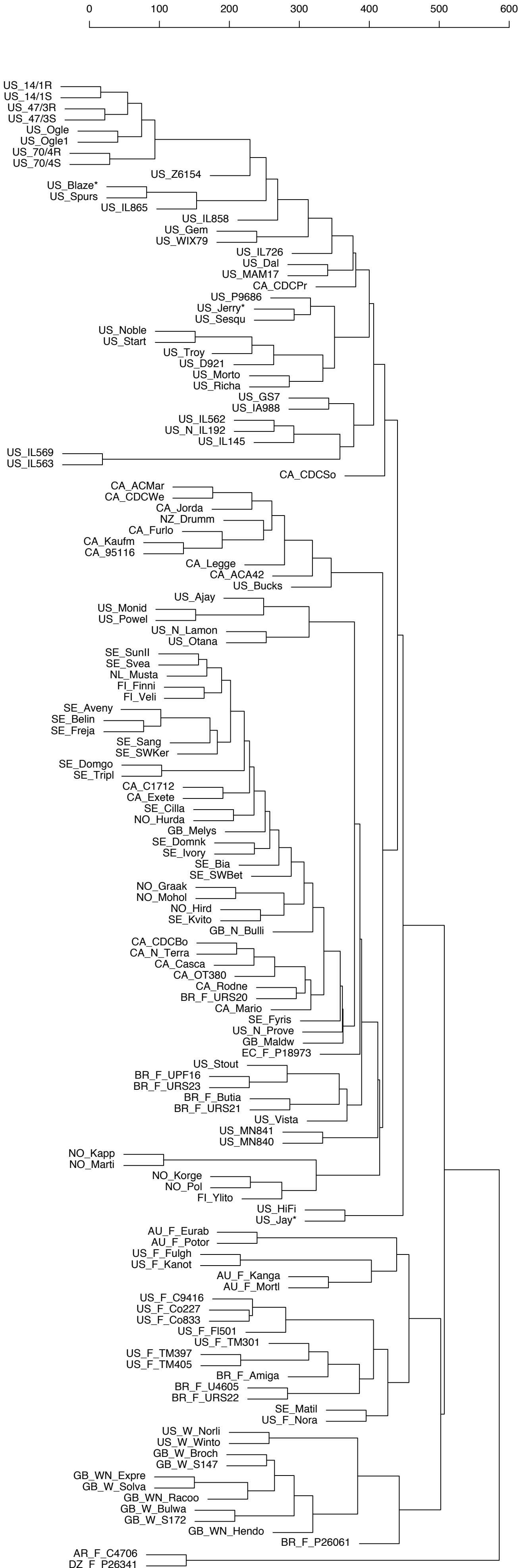

Supplement: Additional File 11 — Cluster analysis of all varieties. UPGMA cluster analysis of germplasm diversity based on 182 oat varieties, including those that were not orthogonal across all three discovery arrays. [file 1471-2164-10-39-S11.pdf]

Average Dissimilarity

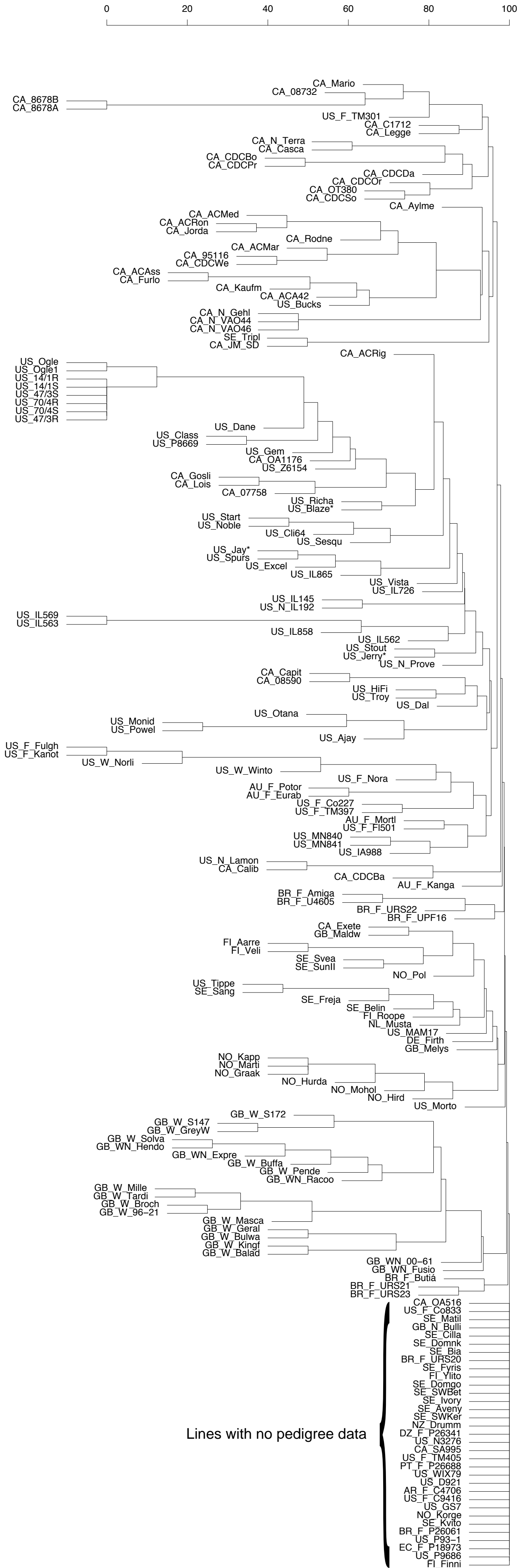

Supplement: Additional File 12 — Pedigree clusters. UPGMA cluster analysis of pedigree distances (D) among 182 oat varieties not orthogonal across all three discovery arrays. [file 1471-2164-10-39-S12.pdf]
