# Supplementary material for: New DArT markers for oat provide enhanced map coverage and global germplasm characterization
Source: BMC Genomics. 2009 Jan 21;10:39. doi: 10.1186/1471-2164-10-39 (PMC2661094; doi:10.1186/1471-2164-10-39)
Supplement: Additional File 8 — Detailed map placements. HTML version of the new 'Kanota' × 'Ogle' DArT framework map, listing approximate placement for additional markers, including more recently published markers. [file 1471-2164-10-39-S8.pdf]

Additional File #8

Kanota x Ogle DArT Framework map showing placements for previously published markers.

Tinker et al., BMC Genomics 2009.

2491 markers in raw data,
373 markers in framework
Total framework map distance: 1989 cM (excluding unlinked markers)
Placement criteria: Minimum Chi Sq. = 7.0
Generated December 9, 2008.

How to read this map:

- Framework cM distances are in leftmost column.
- Framework marker names are in second column.
- Markers placed in the interval following a framework marker are shown to the right
- Dashes symbolize the number of informative recombinations.
- e.g. this indicates the informative recombination fraction and cM distance between framework markers:
- (2/67) {--} = 2 cM
- This indicates the informative recombination fraction between a placed marker (X) and adjacent framework markers:
- (2/56){--X--}(2/53)
- Use "Ctrl-F" to search for a specific marker
- or click to visit a specific linkage group: [1_3_38_break] [1_3_38_X1] [1_3_38_X2] [1_3_38_X3] [1_3_38_X4] [2] [4_12_13] [5_30] [6] [7_10_28] [8] [9] [11_41_20_45] [14] [15] [16_23] [17] [19_25_27] [21_46_31_40] [22_44_18] [24_26_34] [29_43] [32] [33] [36] [37] [39] [42] [46] [47] [48]
- Flag-notes (these refer to special characters in parenthases beside markers)
    1. (**Dup?**) = Markers flagged as potentially duplicated (see below, as explained in Additional File 11).
    2. (**>65%**) = Markers with frequency of + allele greater than 65%.
    3. (**K+0+**) = Markers for which both parents had + alleles.
    4. (**ML?**) = Markers showing preliminary evidence of mapping to multiple locations among other populations.
    5. (**Seg**) = Other Markers (non DArT) with distorted segregation (<35% or >65%)

Linkage Group: 1_3_38_break

Total cM: 9

Framework Map		Placed markers (informative recombination fraction is shown in brackets)
**cM**	**Marker**	
		(2/67) {--} = 2 cM<br>(3/63){---X---}(4/60) **BCD1261B**<br>(2/56){--X--}(2/53) **BCD1514**<br>(4/74){---X----}(5/70) **BCD1562**<br>(4/62){---X--}(2/58) **BCD115A**<br>(5/51){-----X------}(6/50) **CDO216**<br>(2/75){--X--}(3/71) **CDO270B**<br>(1/72){-X--}(3/69) **CDO346A**<br>(1/56){-X-}(1/53) **BCD1851D**<br>(1/59){-X--}(2/56) **BCD1889**<br>(2/59){--X--}(2/56) **BCD342B**<br>(5/61){----X------}(6/55) **CDO109**<br>(1/61){X--}(2/57) **CDO1165B**<br>(1/59){-X--}(2/56) **CDO1314**<br>(1/59){-X--}(2/56) **CDO1365**<br>(2/59){--X--}(2/56) **CDO1385D**<br>(1/54){X-}(2/52) **CDO1420A**<br>(13/55){----------------X----------------}(12/52) **AC03.300**<br>(1/59){-X--}(2/54) **ACO136BRI**<br>(1/59){-X-}(2/54) **ACO145RV**<br>(3/59){---X---}(4/56) **BCD1095B**<br>(2/54){--X--}(2/51) **CDO353B**<br>(1/74){-X--}(3/70) **CDO393A**<br>(6/58){------X----}(4/55) **e2m2_1d**<br>(8/56){--------X--------}(8/53) **e3m7_23x**<br>(0/56){X-}(2/53) **ISU1367**<br>(3/56){---X----}(4/53) **ISU2096**<br>(9/51){-----------X-----------}(8/47) **ISU2182B**<br>(5/59){-----X-----}(5/54) **MOG12BDI**<br>(3/48){---X------}(4/45) **re1m2_3**<br>(6/58){------X------}(6/55) **re6m4_9x**<br>(3/57){---X----}(4/55) **re6m6_9x**<br>(5/47){------X-------}(6/44) **(Seg)UAZ145**<br>(1/53){-X--}(2/50) **UAZ243**

0	**ACO221B**	(4/57){----X------}(6/55) **UMN5528B**<br>(2/47){--X----}(4/44) **UMN5538A**<br>(9/58){---------X-------}(7/55) **UMN5606**<br>(1/57){-X--}(2/55) **UMN5765_2A**<br>(5/56){-----X----}(4/52) **Waaccac150**<br>(3/56){---X---}(3/53) **Waaccac91**<br>(7/48){---------X---------}(7/47) **(Seg)Wacacac632**<br>(6/71){-----X---}(4/66) **WG110B**<br>(3/71){--X---}(4/67) **opt-0350**<br>(2/72){-X--}(3/68) **opt-10202**<br>(2/75){-X--}(3/71) **opt-10481**<br>(3/74){--X---}(4/70) **opt-11446**<br>(3/74){--X---}(4/70) **opt-11446_c**<br>(4/73){---X---}(4/70) **opt-11697**<br>(4/72){---X----}(5/68) **opt-12195**<br>(4/71){---X----}(5/67) **opt-12985**<br>(7/72){-----X------}(7/68) **opt-13130**<br>(3/74){--X---}(4/70) **opt-13864**<br>(2/69){-X--}(3/66) **opt-14091**<br>(4/71){---X---}(4/67) **opt-14381**<br>(3/74){--X---}(4/70) **opt-14665**<br>(3/75){--X---}(4/70) **(Dup?)(ML?)opt-1474**<br>(9/74){-------X-----}(6/70) **opt-14768**<br>(0/65){X--}(2/61) **opt-14922**<br>(2/72){-X--}(3/68) **opt-15208**<br>(6/72){-----X---}(4/68) **opt-15309**<br>(2/74){-X--}(3/70) **opt-15382**<br>(3/74){--X---}(4/70) **opt-15382_c**<br>(2/74){-X--}(3/70) **opt-15597**<br>(4/73){---X----}(5/69) **opt-15736**<br>(2/72){-X--}(3/68) **opt-16230**<br>(2/73){-X--}(2/69) **opt-16552**<br>(4/69){---X----}(4/65) **opt-16558**<br>(3/70){--X---}(4/69) **opt-17050**<br>(2/69){-X---}(4/67) **opt-17083**<br>(2/72){-X--}(2/68) **opt-17611**<br>(2/73){-X--}(3/69) **opt-1803**<br>(2/71){-X--}(3/67) **opt-1817**<br>(2/74){-X--}(3/70) **opt-1817_c**<br>(5/72){----X-----}(6/68) **opt-18233**<br>(5/75){----X-----}(6/71) **opt-18233_c**<br>(3/73){--X---}(4/69) **opt-1881**<br>(4/75){---X----}(5/71) **opt-1881_c**	

		(3/73){--X---}(4/69) **opt-2168**<br>(2/74){-X--}(3/70) **opt-3109**<br>(2/73){-X--}(3/69) **opt-3109_c**<br>(2/71){-X--}(3/67) **opt-3325**<br>(3/74){--X---}(4/70) **opt-3722**<br>(3/71){--X---}(4/67) **(Dup?)(ML?)opt-4159**<br>(8/73){------X-----}(6/70) **opt-4235**<br>(9/75){-------X-----}(6/71) **opt-4235_c**<br>(2/72){-X--}(3/68) **(Dup?)(K+0+)opt-4623**<br>(3/71){--X---}(4/67) **opt-4826**<br>(3/72){--X---}(4/68) **opt-5188**<br>(3/72){--X---}(4/69) **opt-5343**<br>(2/74){-X--}(3/70) **opt-6632**<br>(3/74){--X--}(3/70) **opt-6749**<br>(3/75){--X---}(4/71) **opt-6943**<br>(2/72){-X--}(3/68) **opt-7698**<br>(2/69){-X--}(3/65) **(Dup?)(K+0+)opt-8319**<br>(3/73){--X---}(4/69) **opt-8760**<br>(2/73){-X--}(3/69) **opt-8794**<br>(2/73){-X--}(3/69) **opt-8794_c**<br>(2/74){-X--}(3/70) **(Dup?)(ML?)opt-8810**<br>(3/74){--X---}(4/70) **opt-8892**<br>(3/75){--X---}(4/71) **opt-8924**<br>(2/73){-X--}(3/69) **opt-9234**<br>(1/51){-X---}(3/48) **ubc254s**<br>(10/67){---------X---------}(10/64) **AME018**<br>(3/68){--X--}(2/65) **AME106**
2	**CDO395B**	(8/69) {-------} = 7 cM<br>(10/70){---------X---}(4/75) **CDO1510**<br>(10/57){----------X------}(6/64) **ACO124**<br>(4/56){---X------}(6/62) **re3m7_11x**<br>(8/69){-------X-----}(6/75) **opt-13178**<br>(8/70){------X-----}(8/76) **opt-14477**<br>(4/68){----X-----}(6/74) **opt-17335**<br>(10/65){---------X--------}(10/72) **opt-8250**<br>(8/66){-------X----}(6/70) **11AB4_3RI**
9	**CDO1319A**	

Go to top of this map

Linkage Group: 1_3_38_X1

Total cM: 62

Framework Map		Placed markers (informative recombination fraction is shown in brackets)
**cM**	**Marker**	
0	**BCD1660**	(8/56) {--------} = 8 cM<br>(1/60){-X--------}(8/59) **CDO278**<br>(0/58){X-------}(7/56) **BCD1735**<br>(19/73){------------------X--------}(8/58) **BCD1823A**<br>(8/76){-----X-----}(6/57) **CDO1403A**<br>(2/58){--X-------}(7/57) **ACOR372**<br>(5/52){-----X-----------------}(13/53) **e8m8_8x**<br>(7/58){-------X------------}(11/57) **re1m3_19x**<br>(4/57){----X-------}(7/56) **re1m6_5x**<br>(3/56){---X-----------}(10/55) **UMN5560C**<br>(6/55){------X------------}(10/54) **UMN5895A**<br>(10/73){--------X------------}(11/59) **opt-2065**<br>(9/58){---------X---------------------}(16/57) **ubc372s**<br>(5/50){------X--------}(7/50) **U8PM20**<br>(15/70){--------------X-----------------}(13/53) **cnl81**
8	**CDO1527A**	(4/58) {----} = 4 cM<br>(3/59){---X-----}(6/61) **BCD1443A**<br>(8/58){--------X----------}(10/60) **ISU2064A**<br>(5/56){-----X-----}(5/58) **Wacgcac667**<br>(6/51){-------X-----}(6/67) **AME107**
12	**opt-11327**	(11/76) {---------} = 9 cM<br>(12/72){----------X---------}(11/73) **ACO226B**<br>(10/57){-----------X------------}(11/57) **e5m7_3a**<br>(13/59){----------------X--------------}(13/60) **(Seg)re6m6_7x**<br>(8/70){------X-----------}(13/72) **opt-12915**<br>(8/70){------X-----------}(13/72) **opt-12915_c**<br>(9/75){-------X-----------}(14/76) **opt-17240**<br>(8/71){------X------------}(14/72) **opt-3300**<br>(10/75){--------X}(0/77) **opt-4308**<br>(11/76){---------X}(0/78) **opt-9213**
21	**opt-4308_c**	(12/56) {--------------} = 14 cM
35	**BCD1532A**	(2/55) {--} = 2 cM<br>(2/57){--X-}(2/69) **CDO1435A**

37	**CDO549A**	(10/71) {--------} = 8 cM<br>(6/60){------X---------}(9/60) **BCD907**<br>(10/71){---------X}(0/75) **opt-0011**<br>(10/66){--------X-------}(9/72) **opt-3198**<br>(10/71){---------X}(0/76) **opt-8263**
44	**opt-15341**	(10/55) {-----------} = 11 cM
55	**UMN107A**	(7/54) {--------} = 8 cM<br>(8/55){---------X-----------}(10/58) **UMN706A**
62	**UMN133A**	

Go to top of this map

Linkage Group: 1_3_38_X2

Total cM: 19

Framework Map		Placed markers (informative recombination fraction is shown in brackets)
**cM**	**Marker**	
0	**BCD1405**	(6/55) {------} = 6 cM
6	**BCD454**	(4/62) {---} = 3 cM
9	**WG719B**	(5/77) {---} = 3 cM<br>(12/61){------------X---------}(9/58) **re6m6_14x**
12	**opt-17821**	(7/75) {-----} = 5 cM<br>(6/58){------X-----}(6/59) **UMN433**
17	**opt-3000**	(3/77) {--} = 2 cM<br>(2/47){--X-----}(4/48) **(Seg)ISU1966**<br>(3/58){---X-----}(5/59) **Wacgcac680**<br>(0/78){X--}(3/77) **opt-2648**<br>(3/74){--X}(0/75) **opt-3029**<br>(1/73){-X}(0/75) **opt-4966**<br>(0/51){X---}(3/52) **U8PM32**<br>(5/71){----X-------}(9/72) **AME006**
19	**opt-3029_c**	

Go to top of this map

Linkage Group: 1_3_38_X3

Total cM: 67

Framework Map		Placed markers (informative recombination fraction is shown in brackets)
**cM**	**Marker**	
0	**opt-15764**	(5/69) {----} = 4 cM
4	**CDO393C**	(2/68) {-} = 1 cM<br>(2/71){-X-}(2/73) **opt-9363**
5	**opt-16798**	(5/72) {----} = 4 cM<br>(5/73){----X}(0/76) **opt-14468**<br>(5/72){----X}(0/75) **opt-3986**<br>(6/73){----X}(0/77) **opt-4883**
9	**opt-3986_c**	(13/73) {-----------} = 11 cM
20	**CDO346B**	(7/71) {-----} = 5 cM<br>(6/62){------X----}(4/61) **BCD1261A**<br>(8/68){------X---}(4/67) **ACO221A**<br>(5/59){-----X----}(5/62) **Mdhs**
25	**opt-5671**	(10/76) {--------} = 8 cM<br>(3/57){---X-----}(5/58) **Wacgcac89**<br>(9/73){-------X--}(3/74) **opt-0521**<br>(10/76){--------X}(0/80) **opt-16530**<br>(9/73){-------X-}(1/77) **opt-16737**<br>(10/75){--------X}(0/79) **opt-17280**
33	**opt-16530_c**	(13/77) {----------} = 10 cM<br>(3/58){---X------}(6/55) **Waagcac166**<br>(4/60){----X--------}(8/57) **Wacgcac123**<br>(8/77){------X-----------}(13/74) **opt-5044**
43	**opt-17385**	(2/75) {-} = 1 cM<br>(1/75){-X---}(4/75) **opt-18011**
45	**opt-13202**	(14/75) {------------} = 12 cM
56	**opt-14319**	(1/73) {-} = 1 cM
57	**opt-12734**	(10/71) {--------} = 8 cM<br>(8/74){------X---}(4/73) **UMN149**
65	**BCD1150**	(4/70) {---} = 3 cM
67	**CDO590B**	

Go to top of this map

Linkage Group: 1_3_38_X4

Total cM: 8

Framework Map		Placed markers (informative recombination fraction is shown in brackets)
**cM**	**Marker**	
0	**CDO1449**	(8/61) {--------} = 8 cM<br>(7/74){-----X-}(1/60) **OG41**
8	**OG43**	

Go to top of this map

Linkage Group: 2

Total cM: 71

Framework Map		Placed markers (informative recombination fraction is shown in brackets)
**cM**	**Marker**	
0	**opt-14571**	(14/75) {------------} = 12 cM<br>(2/78){--X---------}(12/75) **AM112**
12	**ACO193CRV**	(5/57) {-----} = 5 cM<br>(4/47){-----X-----}(4/47) **re2m5_12x**<br>(2/52){--X---------}(8/52) **re5m5_11**<br>(5/55){-----X----}(4/55) **re5m5_16x**
17	**CDO1466**	(3/59) {---} = 3 cM
19	**opt-13816_c**	(4/77) {---} = 3 cM<br>(2/60){--X--}(2/60) **BCD149**<br>(1/62){X}(1/62) **BCD342A**<br>(2/61){-X-}(2/60) **ACO134ARI**<br>(3/61){---X---}(3/61) **e6m4_3**<br>(10/60){----------X---------}(9/60) **re1m3_13**<br>(9/51){-----------X----------}(8/50) **re8m3_10**<br>(2/59){--X--}(2/59) **UMN5765_2B**<br>(1/75){-X----}(5/74) **opt-0500**<br>(1/77){-X----}(5/76) **opt-0500_c**<br>(0/68){X--}(2/67) **(Dup?)(K+0+)opt-0829**<br>(1/75){-X----}(5/74) **opt-11115**<br>(1/75){-X----}(5/74) **opt-11263**

		(0/75){X---}(4/74) **opt-12196**<br>(0/78){X---}(4/77) **opt-13816**<br>(0/76){X---}(4/75) **opt-13983**<br>(0/76){X---}(4/75) **opt-13983_c**<br>(0/77){X--}(3/77) **opt-6056**
22	**opt-5100**	(1/78) {-} = 1 cM<br>(3/62){---X--}(2/63) **BCD1095A**<br>(6/58){------X-----}(5/58) **re6m5_1a**<br>(3/77){--X---}(5/79) **opt-0338**<br>(2/76){-X---}(4/78) **opt-10273**<br>(4/77){---X---}(5/79) **opt-2767**<br>(0/72){X}(0/73) **opt-3292**<br>(1/73){-X-}(1/73) **opt-4384**<br>(1/75){-X}(0/77) **opt-5411**<br>(0/77){X-}(1/77) **opt-8324**
23	**opt-5411_c**	(3/61) {---} = 3 cM<br>(10/57){----------X----------}(9/55) **re8m3_7**<br>(6/60){------X--}(2/58) **UMN5528A**<br>(4/77){---X}(0/59) **opt-9892**
25	**BCD1851C**	(3/54) {---} = 3 cM<br>(3/59){---X--}(2/53) **ACO136CRI**<br>(1/59){-X--}(2/66) **opt-12746**<br>(3/60){---X--}(3/68) **opt-13021**<br>(3/60){---X--}(3/68) **opt-13021_c**<br>(2/60){--X-}(1/69) **opt-4430**
28	**opt-13336**	(2/55) {--} = 2 cM<br>(1/60){-X---}(3/51) **AME192**
30	**CDO1158**	(2/62) {--} = 2 cM<br>(1/55){-X--}(2/56) **BCD1819B**<br>(6/61){-----X-------}(8/62) **aa12.550**<br>(1/61){-X-}(1/62) **e1m6_5n**<br>(2/59){--X}(0/60) **e4m2_1fn**<br>(10/53){-----------X--------------}(12/54) **e8m3_13**<br>(4/52){----X----}(4/53) **e8m3_9x**<br>(14/55){-----------------X---------------}(13/56) **e8m8_2n**<br>(11/60){----------X--------------}(14/61) **re1m3_13a**<br>(6/61){-----X-----}(6/62) **re3m7_3n**<br>(6/45){--------X----------}(8/46) **re8m1_9x**<br>(1/58){-X---}(3/59) **Wacccac121**<br>(1/57){-X-}(1/58) **Wacgcac900**

32	**opt-17917_c**	(4/77) {---} = 3 cM<br>(4/79){---X----}(5/76) **CDO270A**<br>(1/67){-X-}(1/65) **BCD1882C**<br>(4/61){----X--}(2/59) **ACO136ARI**<br>(5/55){-----X----}(4/54) **e6m6_20n**<br>(4/62){----X---}(4/59) **ISU2182A**<br>(3/46){---X--}(2/45) **re8m1_9n**<br>(4/60){----X--}(2/58) **UMN341DDI**<br>(4/61){----X-}(2/59) **UMN388**<br>(5/60){-----X--}(2/58) **Wacacac390**<br>(11/77){--------X-------------}(15/74) **(Dup?)(>65%)(K+0+)(ML?)opt-0120**<br>(3/79){--X-}(1/77) **opt-11923**<br>(11/77){--------X-------------}(15/74) **(Dup?)(>65%)(K+0+)(ML?)opt-12215**<br>(0/79){X---}(4/77) **(Dup?)(ML?)opt-17014**<br>(0/80){X---}(4/77) **opt-17917**<br>(3/76){--X----}(5/73) **opt-2644**<br>(2/72){-X}(0/70) **opt-2841**<br>(2/77){-X--}(3/74) **opt-8677**<br>(13/79){----------X---------------}(17/76) **(Dup?)(>65%)(K+0+)opt-9794**<br>(4/62){---X---}(3/60) **ubc263s**<br>(11/60){-----------X--------}(8/58) **ubc360s**<br>(5/53){-----X-----}(5/51) **U8PM33**
34	**opt-2898**	(3/77) {--} = 2 cM<br>(7/50){--------X-------}(6/52) **e2m3_4ax**<br>(6/60){------X----}(5/62) **re6m4_20x**<br>(3/77){--X}(0/80) **opt-11020**<br>(1/74){-X}(0/77) **opt-1176**<br>(2/68){--X----}(5/71) **opt-12698**<br>(3/76){--X-}(1/79) **opt-13517**<br>(3/66){--X-----}(6/68) **opt-15709**<br>(2/76){-X}(0/79) **opt-15755**<br>(2/73){-X---}(4/76) **opt-16403**<br>(2/73){-X---}(4/76) **opt-16403_c**<br>(3/77){--X}(0/80) **opt-2494**<br>(8/68){-------X-------}(9/71) **(Dup?)(>65%)opt-8354_c_rp**<br>(8/68){-------X-------}(9/71) **(Dup?)(>65%)opt-8354_rp**<br>(2/56){--X----}(4/58) **ubc179Os**
36	**opt-1176_c**	(12/80) {---------} = 9 cM

46	**BCD1829A**	(3/78) {--} = 2 cM<br>(2/59){--X----}(4/59) **BCD1552**<br>(6/58){------X---------}(8/58) **CSU40b**<br>(3/56){---X---}(3/56) **e8m8_7x**<br>(8/47){-----------X-------------}(10/47) **re4m8_14x**<br>(6/57){------X---------}(8/57) **re6m5_4x**<br>(4/71){---X---}(4/69) **opt-2211**
48	**CDO1342**	(4/76) {---} = 3 cM<br>(8/60){-------X---------}(9/59) **CDO1454**<br>(4/63){---X----}(5/62) **UMN411**<br>(11/60){-----------X-----------}(10/59) **UMN509B**
51	**(Dup?)(ML?)opt-15329**	(8/54) {---------} = 9 cM<br>(8/50){----------X-}(1/45) **e1m2_8x**<br>(15/61){-----------------X-----}(4/53) **re4m6_1bx**<br>(15/61){-----------------X-----}(4/53) **re4m6_1x**<br>(9/57){---------X--}(2/51) **UMN5271_2A**<br>(10/58){-----------X---}(2/52) **UMN5846**<br>(12/70){----------X-}(1/49) **opt-12290**<br>(13/77){----------X---}(4/54) **opt-14185**<br>(12/76){---------X------}(6/55) **opt-2760**<br>(12/76){---------X-----}(4/54) **opt-2760_c**<br>(11/74){---------X----}(4/51) **opt-3339**
60	**CDO539A**	(10/55) {-----------} = 11 cM<br>(2/54){-X-----------}(11/62) **BCD1779**<br>(4/50){-----X-------------}(12/58) **e5m5_3x**<br>(0/45){X---------}(8/52) **re6m5_12x**<br>(2/52){-X--------}(8/60) **Wacacaa165**<br>(1/54){X----------}(13/78) **opt-17084**<br>(1/53){X---------}(12/77) **opt-8170**<br>(10/54){-----------X----------------------}(18/62) **II2.3b**
71	**(Dup?)(>65%)(ML?)opt-3592**	

Go to top of this map

Linkage Group: 4_12_13

Total cM: 167

Framework Map		Placed markers (informative recombination fraction is shown in brackets)
**cM**	**Marker**	

0	**opt-6427**	(14/59) {----------------} = 16 cM<br>(14/72){-------------X-----}(6/60) **ACO226C**<br>(14/54){-------------------X------}(6/58) **(Seg)Waagcac146**<br>(13/71){-----------X-------}(7/60) **opt-0913**
16	**(Seg)BCD1443B**	(15/63) {----------------} = 16 cM<br>(8/56){--------X-----------------}(14/56) **(Seg)ISU2064B**<br>(5/59){-----X-------------}(15/74) **opt-6451**<br>(13/62){--------------X----------------------}(18/62) **(Seg)L8M3.8**
32	**opt-11926_c**	(1/74) {-} = 1 cM<br>(2/74){--X--}(3/72) **CDO1435B**<br>(9/75){-------X-----}(7/72) **CDO460A**<br>(1/76){-X-}(2/72) **CDO549B**<br>(2/61){--X--}(2/58) **e1m6_9x**<br>(0/77){X-}(2/74) **opt-11926**<br>(0/71){X}(0/70) **opt-7088**<br>(0/76){X-}(1/72) **opt-8259**
33	**opt-5725**	(4/58) {----} = 4 cM<br>(12/53){---------------X---------------}(12/54) **re5m7_12ax**
37	**UMN107B**	(2/61) {--} = 2 cM<br>(3/61){---X---}(4/63) **BCD876**<br>(2/50){--X---}(3/51) **re8m1_2x**<br>(7/50){--------X-------}(6/51) **(Seg)re8m1_4x**<br>(1/61){-X---}(3/61) **UMN706C**<br>(10/59){----------X--------------}(13/61) **ubc189s**
38	**opt-5421_c**	(4/78) {--} = 2 cM<br>(10/61){----------X-----}(6/60) **BCD1532B**<br>(4/76){--X--}(3/74) **CDO1327**<br>(4/79){---X---}(4/78) **UMN133B**<br>(0/79){X--}(2/77) **opt-0148**<br>(0/80){X--}(4/78) **opt-5421**<br>(7/68){------X-----}(6/66) **AME066**
41	**CDO638**	(6/57) {------} = 6 cM<br>(8/55){--------X----}(4/53) **re5m5_17x**<br>(6/68){-----X--}(2/52) **ALrk1A1_E**
47	**BCD1876**	(6/52) {-------} = 7 cM<br>(1/20){---X}(0/33) **CDO187A**<br>(4/53){----X-------}(6/51) **e5m5_18x**

54	**opt-17024**	(7/68) {------} = 6 cM<br>(6/42){--------X---}(3/47) **BCD1495**<br>(8/54){--------X--}(2/58) **BCD1154**<br>(10/66){--------X------}(8/73) **CDO1174B**<br>(6/54){-------X---}(4/58) **ACO238RI**<br>(6/53){------X---}(3/57) **BCD1127B**<br>(5/56){-----X----}(4/60) **CDO585B**<br>(8/47){----------X-------}(6/49) **e3m7_1d**<br>(7/45){---------X--------}(7/50) **e5m3_5ax**<br>(8/67){-------X----}(6/74) **OG49**<br>(3/54){---X-------}(7/57) **re2m2_7x**<br>(9/49){-----------X-----------}(9/52) **re3m7_1dx**<br>(6/55){------X---}(3/59) **UMN5517**<br>(1/51){-X----}(4/54) **Wacacaa540**<br>(4/54){----X-}(1/59) **Wacgcaa138**<br>(9/68){--------X-}(2/74) **opt-12259**<br>(1/65){-X-------}(8/69) **opt-15886**<br>(4/61){----X--}(2/67) **opt-4177**<br>(7/67){------X}(0/73) **opt-5239**<br>(8/67){-------X-}(1/73) **opt-5239_c**
59	**opt-5635**	(13/76) {----------} = 10 cM<br>(13/72){-----------X}(0/75) **(Dup?)(>65%)opt-13205**<br>(10/72){--------X}(0/75) **(Dup?)(>65%)opt-2654**
70	**(Dup?)(>65%)opt-2654_c**	(22/75) {----------------------} = 22 cM
92	**opt-13895**	(3/73) {--} = 2 cM<br>(18/73){------------------X-------------}(14/73) **CDO187**<br>(10/73){--------X------}(8/72) **CDO1174A**<br>(11/50){--------------X----------}(8/50) **CDO480**<br>(6/50){-------X------}(5/50) **e2m5_1a**<br>(13/74){-----------X---------}(11/73) **(Seg)PTA71A**<br>(5/73){----X--}(3/72) **opt-0157**<br>(7/72){-----X----}(5/72) **opt-6644**<br>(8/69){-------X-----}(6/68) **(Seg)AME051**
94	**opt-10908**	(26/71) {---------------------------------} = 33 cM

127	**opt-16128_c**	(14/75) {------------} = 12 cM<br>(5/71){----X----------------}(17/73) **BCD1482B**<br>(4/74){--X-------------------}(20/77) **CDO1473B**<br>(4/74){--X-------------------}(20/77) **CDO1385F**<br>(12/59){-------------X------------------------------}(21/60) **avnA**<br>(4/49){----X-------------}(10/48) **e8m1_6**<br>(2/74){-X----------------}(18/77) **POP6ADIRI**<br>(11/55){-------------X--------------}(12/56) **re5m7_14x**<br>(7/61){-------X----------------------}(18/62) **re6m4_3a**<br>(6/57){------X------------}(11/58) **UMN101**<br>(10/56){------------X--------------}(12/57) **UMN194A**<br>(2/74){-X----------------}(18/77) **UMN214A**<br>(5/55){-----X--------------}(12/56) **UMN282**<br>(9/59){---------X----------------}(14/60) **UMN30**<br>(6/71){----X---------}(12/74) **UMN341A**<br>(4/72){---X------------------}(20/75) **UMN853**<br>(6/57){------X------------}(11/58) **UMN869**<br>(4/72){---X-------------}(15/75) **opt-14336**<br>(2/73){-X--------------}(16/75) **opt-15025**<br>(0/76){X------------}(14/75) **opt-16128**<br>(3/72){--X-----------}(13/74) **opt-16923**<br>(2/72){-X------------}(14/73) **opt-1755**<br>(4/69){---X-----------}(13/72) **opt-18225**<br>(1/74){-X-------------}(16/77) **opt-4915**<br>(2/75){-X--------------}(17/78) **opt-4915_c**<br>(2/73){-X------------}(14/75) **opt-6235**<br>(2/75){-X------------}(15/78) **opt-6798**<br>(4/72){---X------------}(14/74) **ALrk1A1_C**<br>(2/39){--X--------------}(8/39) **LR10_1A**<br>(3/47){---X------------}(9/47) **U8PM25**
139	**opt-15746_c**	(3/79) {--} = 2 cM<br>(6/61){-----X------}(6/62) **CDO309A**<br>(10/62){----------X--------}(9/63) **re3m7_1c**<br>(3/75){--X}(0/76) **opt-13043**<br>(8/72){------X----}(5/73) **opt-13158_rp**<br>(0/78){X--}(3/79) **opt-15746**<br>(3/75){--X}(0/76) **opt-1847**<br>(7/79){-----X---}(4/80) **opt-2612**<br>(4/77){---X-}(1/78) **opt-8515**<br>(0/70){X-}(2/71) **opt-9361**

141	**opt-1847_c**	(4/61) {----} = 4 cM<br>(12/63){------------X-------}(7/61) **CDO1423A**<br>(4/61){----X---}(3/59) **Wacgcaa243**
144	**CDO718B**	(11/59) {------------} = 12 cM
156	**CDO1173A**	(6/72) {-----} = 5 cM<br>(2/59){--X----}(4/56) **CDO1519BDI**<br>(8/76){-----X----}(6/73) **CDO220**<br>(6/57){-----X-------}(6/54) **CDO580**<br>(3/60){---X-------}(7/58) **CDO618A**<br>(5/48){------X------}(5/46) **e5m5_26x**<br>(5/74){----X-------}(9/71) **UMN295B**<br>(10/75){-------X----}(5/72) **UMN333**<br>(6/54){------X------------}(10/52) **UMN5301_3C**<br>(7/76){-----X-}(1/74) **opt-10116**<br>(5/75){----X---}(4/72) **opt-11841**<br>(5/73){----X--}(3/71) **opt-12342**<br>(4/72){---X-}(2/70) **(Dup?)(K+0+)opt-1284**<br>(5/76){----X---}(4/74) **(Dup?)(K+0+)opt-1284_c**<br>(3/73){--X-----}(7/71) **opt-13290**<br>(6/75){----X----}(5/73) **opt-1459**<br>(5/74){----X---}(4/72) **opt-16584**<br>(8/67){-------X-}(1/66) **(Dup?)(K+0+)opt-16702_rp**<br>(2/77){-X----}(6/73) **opt-17735**<br>(5/74){----X---}(4/72) **opt-18327**<br>(4/74){---X-}(2/72) **opt-3648**<br>(4/76){---X--}(3/73) **opt-5128**<br>(5/77){---X---}(4/74) **opt-5128_c**<br>(4/73){---X---}(4/69) **opt-5230_rp**<br>(5/74){----X---}(4/72) **opt-6016**<br>(5/69){----X-----}(6/67) **(Dup?)(K+0+)opt-8212**<br>(5/73){----X---}(4/71) **opt-9941**<br>(9/69){--------X--}(3/66) **AME177**
161	**opt-11135**	(6/53) {------} = 6 cM
167	**re4m2_7x**	

Go to top of this map

Linkage Group: 5_30

Total cM: 114

Framework Map		Placed markers<br>(informative recombination fraction is shown in brackets)
**cM**	**Marker**	
0	**UMN5047**	(4/56) {----} = 4 cM<br>(8/57){--------X---------------}(12/53) **ACOR254A**<br>(4/43){-----X----------}(7/42) **e2m3_12**
4	**opt-14924_c**	(2/74) {-} = 1 cM<br>(1/53){-X--}(2/59) **CDO564RI**<br>(8/55){---------X--------}(8/61) **e6m6_13x**<br>(5/55){-----X----}(5/61) **re1m3_26x**<br>(8/47){----------X-----------}(9/50) **re6m5_2**<br>(2/55){-X}(1/61) **UMN212**<br>(4/69){---X----}(5/75) **UMN97B**<br>(0/74){X-}(2/74) **opt-14924**
5	**opt-17862**	(3/74) {--} = 2 cM<br>(7/79){-----X----}(6/73) **CDO1319B**<br>(3/62){---X-}(1/58) **ACOR209B**<br>(7/62){------X----}(4/58) **ACOR242**<br>(4/75){---X---}(4/69) **CDO370**<br>(6/79){----X--}(2/73) **CDO949**<br>(4/50){----X---}(3/46) **e1m2_9**<br>(9/61){---------X-------}(7/57) **e1m3_3a**<br>(4/63){---X--}(2/59) **e1m6_6**<br>(8/60){--------X-----}(5/56) **e2m2_16x**<br>(3/60){---X-}(1/56) **e2m2_5**<br>(4/56){----X----}(4/53) **e2m5_1x**<br>(6/63){-----X----}(4/59) **e3m7_2**<br>(2/60){--X}(0/56) **e4m6_6a**<br>(9/49){-----------X-------}(6/47) **e4m8_4**<br>(4/61){----X--}(2/57) **e6m4_23x**<br>(5/62){----X---}(3/58) **e6m4_3b**<br>(14/54){------------------X----------------}(12/51) **e8m8_5b**<br>(2/55){--X}(0/51) **MDHf**<br>(7/60){-------X-----}(5/56) **re1m3_15**<br>(2/59){--X}(0/55) **re1m6_14**<br>(3/50){---X-}(1/47) **re2m2_17**<br>(2/61){--X}(0/58) **re3m7_6**<br>(2/45){--X}(0/42) **re3m7_8**<br>(4/50){----X--}(2/47) **re6m5_13x**<br>(2/45){--X-}(1/42) **re6m5_5**<br>(4/58){----X--}(2/54) **re8m3_6**<br>(6/61){------X----}(4/57) **WG605**

		(4/69){---X-}(1/64) **opt-10596**<br>(4/76){---X--}(3/70) **opt-11724**<br>(2/79){-X-}(1/73) **(Dup?)(ML?)opt-1463**<br>(9/72){-------X----}(5/66) **opt-15287**<br>(2/80){-X-}(1/74) **opt-16874**<br>(4/77){---X-}(1/71) **opt-17188**<br>(3/76){--X}(0/70) **opt-17188_c**<br>(3/73){--X}(0/67) **opt-9668**
7	**opt-13894**	(14/67) {--------------} = 14 cM
21	**opt-15113**	(1/58) {-} = 1 cM
22	**CDO1396**	(2/63) {--} = 2 cM<br>(8/61){--------X--------}(8/61) **re1m3_3x**<br>(2/62){--X}(0/77) **opt-17314**<br>(2/62){--X}(0/77) **opt-2746**
23	**opt-17314_c**	(9/68) {--------} = 8 cM<br>(5/63){----X---}(4/62) **CDO572A**<br>(6/63){-----X----}(5/62) **ISU2000**<br>(7/61){-------X-----}(6/60) **Waaccac336**<br>(7/59){-------X----}(4/58) **Waaccac365**<br>(5/59){-----X---}(3/58) **Wacgcaa226**
31	**CDO1165A**	(12/68) {-----------} = 11 cM
43	**CDO393B**	(8/76) {------} = 6 cM<br>(5/51){-----X-----------}(9/50) **(Seg)BCD1482A**<br>(6/74){----X---------}(10/72) **CDO1242**<br>(2/62){--X--------}(8/60) **CDO1420B**<br>(14/62){---------------X------------------}(16/61) **avnB**<br>(4/77){--X-------}(9/74) **POP6DI**<br>(4/77){--X-------}(9/74) **UMN214B**
49	**CDO1173B**	(2/77) {--} = 2 cM<br>(0/59){X-}(1/60) **CDO1519ADI**<br>(1/58){-X--}(2/60) **CDO1423B**<br>(0/58){X--}(2/59) **AB02.275**<br>(0/60){X--}(2/62) **CDO618B**<br>(6/52){-------X---------}(8/53) **e1m2_3b**<br>(3/58){---X---}(3/60) **e5m7_5**<br>(3/59){---X----}(4/61) **e5m7_6**<br>(0/43){X-}(1/45) **e6m5_16x**<br>(8/58){-------X--------}(8/60) **estB**<br>(2/59){--X----}(4/61) **ISU916A**<br>(3/60){---X----}(5/62) **re1m6_3n**

		(4/59){----X-----}(6/61) **re6m6_6x**<br>(2/54){--X---}(3/56) **re8m3_7x**<br>(0/61){X---}(3/63) **UMN106**<br>(2/34){--X----}(3/37) **(Seg)UMN13B**<br>(4/76){---X------}(8/79) **UMN38**<br>(1/36){-X----}(3/39) **UMN5084B**<br>(4/53){----X------}(6/55) **UMN5301_3D**<br>(1/57){-X--}(2/59) **UMN815A**<br>(5/52){-----X------}(6/54) **Wacccac541**<br>(0/59){X--}(2/61) **Wacgcaa418**<br>(6/71){----X----}(6/73) **opt-9573_rp**<br>(2/71){-X--}(3/73) **ALrk1A1_B**
51	**opt-1672_c**	(1/80) {-} = 1 cM<br>(2/74){-X--}(3/74) **opt-10977**<br>(2/74){-X--}(3/74) **opt-12269**<br>(3/79){--X---}(4/79) **opt-12269_c**<br>(0/77){X-}(1/77) **opt-1672**<br>(0/80){X-}(1/80) **opt-17840**<br>(0/80){X-}(1/80) **opt-1971**<br>(0/76){X-}(1/76) **opt-4140**<br>(3/75){--X---}(4/75) **opt-4439**<br>(2/78){-X--}(3/78) **opt-5662**<br>(1/78){-X}(0/78) **(Dup?)(ML?)opt-6005**<br>(4/68){---X----}(5/68) **AME076**
51	**opt-17150**	(4/78) {---} = 3 cM<br>(13/73){-----------X---------}(11/71) **UMN295C**<br>(10/77){--------X---}(4/75) **UMN51A**<br>(4/77){---X}(0/77) **opt-10148**<br>(4/77){---X}(0/77) **opt-10413**<br>(4/77){---X}(0/77) **opt-17970**<br>(4/78){---X}(0/78) **opt-18140**<br>(6/76){----X----}(6/74) **opt-4086**<br>(9/73){-------X----}(5/71) **AME131**
54	**opt-17970_c**	(4/72) {---} = 3 cM<br>(4/62){---X------}(6/59) **BCD1580**<br>(6/74){----X----}(5/69) **UMN5904B**
57	**opt-13760**	(3/59) {---} = 3 cM<br>(4/59){----X-}(1/63) **BCD1230A**<br>(2/59){--X-}(1/63) **BCD1897A**
60	**WG282**	(8/61) {--------} = 8 cM

67	**UMN28BRV**	(2/60) {--} = 2 cM<br>(3/55){---X-----}(6/68) **CDO20**<br>(4/60){---X---}(4/61) **CDO1081**<br>(3/60){---X----}(6/77) **CDO1312B**<br>(2/60){--X-----}(7/77) **CDO1358A**<br>(4/61){---X---}(4/62) **CDO63**<br>(2/61){--X---}(5/77) **CDO770A**<br>(7/51){--------X-------}(6/52) **e2m3_8**<br>(4/60){----X---}(3/61) **re4m6_11x**<br>(5/57){-----X-----}(7/73) **UMN442A**<br>(2/60){--X-}(2/76) **opt-6125**
69	**opt-9975**	(22/74) {----------------------} = 23 cM
91	**(Dup?)(>65%)opt-17294**	(17/72) {----------------} = 16 cM<br>(1/72){-X-----------------}(18/73) **(Dup?)(>65%)opt-12088**<br>(1/73){-X----------------}(17/73) **(Dup?)(>65%)opt-13397**<br>(1/74){-X-----------------}(19/76) **(Dup?)(>65%)opt-13397_c**<br>(1/69){-X----------------}(17/71) **(Dup?)(>65%)opt-14678**<br>(1/73){-X-----------------}(18/74) **(Dup?)(>65%)opt-9106**
107	**opt-14917**	(1/76) {-} = 1 cM<br>(1/58){X}(1/58) **BCD1130**<br>(2/61){-X-}(2/61) **CDO344**<br>(2/61){--X--}(2/61) **BCD1850**<br>(2/61){--X--}(2/61) **BCD1851A**<br>(2/59){--X--}(2/59) **CDO1335**<br>(5/61){----X----}(5/62) **aa12.1050**<br>(3/59){---X---}(3/59) **ACO128RI**<br>(3/64){--X---}(4/67) **CDO677A**<br>(8/77){------X------}(8/79) **CDO938**<br>(9/54){----------X----------}(9/54) **CSU36a**<br>(0/42){X}(0/42) **e3m7_26x**<br>(6/62){-----X----}(5/61) **e4m6_17x**<br>(1/52){-X-}(1/51) **e5m3_8x**<br>(8/52){---------X---------}(8/51) **phytoB**<br>(2/60){--X--}(2/60) **PX_5**<br>(7/61){-------X-----}(6/61) **re2m2_6x**<br>(2/59){--X--}(2/59) **re3m7_15x**<br>(2/62){--X--}(2/62) **re3m7_9x**<br>(5/53){-----X-----}(5/53) **re5m7_13bx**<br>(3/58){---X---}(3/58) **re5m7_13x**<br>(1/60){-X-}(1/60) **UMN109**<br>(2/59){--X--}(2/59) **UMN207B**

		(2/62){--X--}(2/62) **UMN487**<br>(1/54){-X-}(1/53) **UMN5109_2**<br>(4/55){----X----}(4/54) **UMN5351**<br>(1/53){-X-}(1/52) **UMN5697**<br>(2/57){--X--}(2/57) **Waagcac342**<br>(7/57){-------X-------}(7/57) **Wacacaa725**<br>(2/54){--X--}(2/54) **Wacacac452**<br>(0/60){X}(0/60) **Wacgcaa234**<br>(0/59){X}(0/59) **Wacgcac131**<br>(5/77){---X----}(6/79) **WG645**<br>(9/74){-------X-------}(9/73) **opt-11819**<br>(1/62){-X-}(1/62) **AM83**
108	**opt-14420**	(1/79) {-} = 1 cM<br>(6/53){------X------}(6/53) **e8m8_10x**<br>(11/59){------------X-----------}(11/60) **(Seg)re2m2_5a**<br>(11/54){-------------X-------------}(11/54) **(Seg)UAZ213b**<br>(1/77){-X}(0/78) **opt-14157**<br>(0/78){X-}(1/79) **opt-14653**<br>(3/74){--X-}(2/75) **opt-15695**<br>(2/76){-X-}(1/77) **opt-18018**<br>(4/75){---X--}(3/76) **opt-4545**<br>(2/78){-X-}(1/79) **opt-4545_c**<br>(1/76){-X}(0/77) **opt-9486**<br>(0/77){X-}(1/78) **opt-9495**<br>(3/68){--X---}(4/69) **(Dup?)(>65%)opt-9916**<br>(3/53){---X---}(3/54) **U71PM16**<br>(3/70){--X---}(4/71) **AME112**
109	**opt-9486_c**	(8/79) {-----} = 5 cM<br>(11/62){-----------X-------}(8/62) **aa12.1075**<br>(5/58){-----X----}(4/58) **e6m5_1x**<br>(4/55){----X----}(4/55) **re2m3_1x**<br>(16/61){------------------X-----------------}(15/61) **(Seg)re6m6_6n**
114	**UMN407**	

Go to top of this map

Linkage Group: 6

Total cM: 97

**Framework Map**	**Placed markers<br>(informative recombination fraction is shown in brackets)**

cM	Marker	
0	**opt-3531_c**	(14/62) {--------------} = 14 cM<br>(9/63){--------X-------------------}(16/62) **CDO447**<br>(4/62){---X----------------}(14/61) **e1m6_8x**<br>(0/77){X-------------}(12/60) **opt-0943**<br>(0/70){X---------------}(12/55) **opt-2401**<br>(0/78){X------------}(12/60) **opt-3531**<br>(1/77){-X------------}(12/59) **opt-5868**
14	**BCD1867**	(2/62) {--} = 2 cM<br>(1/60){X-}(2/76) **CDO1407B**<br>(2/60){--X-----}(6/61) **re1m3_17**<br>(8/60){--------X---------}(9/61) **(Seg)UMN826**<br>(2/61){--X}(0/77) **opt-11536**<br>(2/60){-X}(0/77) **opt-13262**<br>(1/56){-X}(0/73) **opt-1602**<br>(2/60){--X}(0/77) **opt-16457**<br>(4/62){---X-}(1/80) **opt-5874**<br>(2/59){--X}(0/76) **opt-9593**<br>(4/61){---X-}(1/78) **opt-9990**
16	**opt-11536_c**	(15/80) {------------} = 12 cM
28	**opt-8723**	(10/78) {-------} = 7 cM<br>(7/61){-------X---}(3/60) **(Seg)BCD1311**<br>(3/63){---X------}(7/62) **(Seg)BCD1802**<br>(8/58){--------X-}(1/57) **CDO1090A**<br>(11/78){--------X----}(6/76) **CDO1199A**<br>(15/59){------------------X--------------------}(16/58) **AC03.480**<br>(11/71){---------X-----}(6/69) **(Seg)ACO182C**<br>(11/71){---------X-----}(6/69) **(Seg)CDO358CDI**<br>(9/59){---------X-}(1/58) **(Seg)CDO407**<br>(12/79){----------X--}(4/77) **CDO595**<br>(6/61){-----X-----}(5/60) **(Seg)e6m4_16x**<br>(11/62){-----------X-------}(7/61) **ISU1755A**<br>(7/49){--------X---}(3/48) **re3m7_24x**<br>(12/59){-------------X--------}(8/58) **re4m2_6x**<br>(5/53){-----X-----}(5/52) **re6m4_17x**<br>(11/62){-----------X-------}(7/61) **UMN136**<br>(7/60){-------X--------}(8/59) **Wacacac1140**<br>(1/78){-X---------}(11/76) **opt-2647**<br>(1/78){-X---------}(11/76) **opt-2647_c**
36	**opt-6402**	(10/78) {-------} = 7 cM

43	**opt-2953_c**	(1/76) {-} = 1 cM<br>(18/78){---------------X---------------}(17/74) **(Dup?)(>65%)opt-11352**<br>(0/80){X-}(1/76) **opt-2953**<br>(9/73){-------X--------}(9/69) **opt-3258**
44	**opt-15558**	(2/73) {-} = 1 cM<br>(4/58){----X-}(1/59) **AC06.480**<br>(8/49){----------X------}(5/50) **re1m2_4x**<br>(1/73){-X}(0/75) **opt-5563**<br>(3/40){----X-}(1/40) **(Seg)R4_1_CRV**<br>(3/60){---X--}(2/61) **III2.18a**
45	**opt-1172**	(3/60) {---} = 3 cM<br>(3/60){---X}(0/61) **CDO1467B**<br>(8/76){-----X}(0/60) **CDO1378B**<br>(4/51){----X------}(5/50) **re5m5_1ax**<br>(10/71){--------X-------}(7/58) **(Dup?)(>65%)opt-4120**<br>(10/70){--------X------}(6/56) **(Dup?)(>65%)opt-4120_c**
48	**ISU1146A**	(9/61) {---------} = 9 cM
56	**opt-6986**	(9/75) {-------} = 7 cM
63	**opt-12813**	(5/71) {----} = 4 cM<br>(2/73){--X--}(2/72) **ACO193ARV**<br>(3/59){---X-}(1/62) **CDO56**<br>(5/58){-----X---}(3/61) **UMN361**
67	**BCD1872B**	(9/70) {-------} = 7 cM
74	**opt-16916**	(4/60) {----} = 4 cM<br>(2/64){--X--}(2/61) **CDO420C**<br>(1/74){-X---}(4/62) **opt-17158**<br>(3/66){--X----}(4/53) **AME105**
79	**CDO1313**	(2/58) {-} = 1 cM<br>(2/61){--X-}(1/58) **BCD1939**<br>(4/60){---X--}(2/58) **CDO1357B**<br>(2/60){-X----}(4/57) **ACO220DI**<br>(6/58){------X-------}(7/56) **e5m7_3x**<br>(4/54){---X--}(2/51) **PGD**<br>(4/59){----X----}(4/57) **UMN815B**<br>(0/59){X--}(2/56) **Wacgcac164**
80	**opt-5330**	(5/73) {----} = 4 cM<br>(10/69){---------X--------}(10/74) **CDO1419**<br>(0/74){X----}(5/73) **opt-2810_c**<br>(4/74){---X-}(1/79) **opt-6657**

84	**opt-6657_c**	(4/75) {---} = 3 cM<br>(9/76){-------X---}(4/73) **CDO82**<br>(1/76){-X----}(5/73) **(Dup?)(ML?)opt-10258**<br>(0/74){X---}(4/71) **opt-10359**<br>(0/77){X---}(4/74) **opt-10359_c**<br>(0/78){X---}(4/76) **opt-10973**<br>(0/77){X---}(4/74) **opt-12177**<br>(0/68){X---}(4/67) **opt-5924**<br>(0/78){X---}(4/74) **opt-7113**<br>(7/72){-----X-------}(8/69) **AME045**
86	**opt-5861**	(5/69) {----} = 4 cM<br>(3/58){---X-}(1/58) **ACO117DI**<br>(2/49){--X------}(5/49) **e5m5_12**<br>(5/69){----X}(0/73) **opt-12737_c**
90	**opt-10823**	(5/56) {-----} = 5 cM<br>(6/56){------X------}(6/55) **ISU1372A**<br>(7/59){-------X------}(6/58) **UMN356**
95	**PGI**	(2/57) {--} = 2 cM<br>(1/58){-X----}(4/59) **BCD1340**<br>(1/58){-X---}(4/66) **CDO1473A**<br>(1/58){X---}(4/59) **CDO309B**<br>(1/58){-X---}(4/66) **CDO1385B**<br>(4/58){---X-----}(6/59) **avnC**<br>(3/55){---X-----}(5/56) **CDO708A**<br>(2/59){-X----}(4/60) **CDO718A**<br>(4/54){----X------}(6/55) **e1m3_28x**<br>(7/57){-------X--------}(8/58) **e2m2_8x**<br>(3/59){---X----}(4/60) **e3m7_8x**<br>(5/53){-----X----}(4/54) **e8m8_5x**<br>(5/56){-----X-----}(6/57) **estA**<br>(1/56){-X--}(2/57) **estC**<br>(8/59){-------X-----------}(10/60) **ISU1774A**<br>(2/58){--X-----}(5/59) **ISU916B**<br>(2/57){-X----}(4/58) **MOG12ADI**<br>(1/57){-X---}(3/58) **re3m7_13x**<br>(2/59){-X----}(4/60) **UMN162**<br>(2/59){-X----}(4/60) **UMN249**<br>(4/57){----X------}(8/72) **UMN295A**<br>(1/55){-X----}(4/57) **UMN341CDI**<br>(2/58){--X-----}(7/73) **UMN420**<br>(2/58){--X-----}(7/73) **UMN753**<br>(1/55){-X----}(4/57) **Wacacaa205**

		(0/56){X---}(4/70) **opt-1116**<br>(0/59){X----}(5/73) **opt-11276**<br>(0/56){X---}(4/70) **opt-13393**<br>(1/58){-X--}(3/73) **opt-15437**<br>(0/55){X--}(3/69) **opt-17123**<br>(0/58){X--}(3/73) **opt-18057**<br>(0/54){X}(0/70) **opt-3771**<br>(1/59){-X--}(3/74) **opt-3944**<br>(2/58){--X-}(2/74) **opt-4219**<br>(2/58){--X-}(1/73) **opt-4219_c**<br>(0/56){X---}(4/70) **opt-5768**<br>(1/57){-X-}(1/73) **opt-6334**<br>(1/57){-X--}(3/73) **opt-7194**<br>(0/58){X----}(5/72) **opt-7195**<br>(2/57){--X}(0/75) **opt-8163**<br>(0/57){X---}(4/70) **opt-9221**<br>(1/37){-X---}(2/39) **2AB1-5**<br>(2/53){--X---}(4/69) **ALrk1A1_D**<br>(1/37){-X----}(3/39) **LR10_1B**
97	**opt-8163_c**	

Go to top of this map

Linkage Group: 7_10_28

Total cM: 172

Framework Map		Placed markers (informative recombination fraction is shown in brackets)
**cM**	**Marker**	
0	**re1m6_22x**	(8/55) {---------} = 9 cM<br>(4/46){-----X---}(3/50) **e1m2_9x**<br>(8/55){---------X-------}(7/61) **e4m6_9x**<br>(8/56){--------X-----}(6/62) **UMN409**
9	**BCD1856**	(8/62) {-------} = 7 cM<br>(11/47){----------------X-------------}(10/48) **e8m1_5x**
16	**CDO1388**	(4/60) {----} = 4 cM<br>(5/34){---------X---------------------------}(7/21) **CDO1461B**<br>(2/78){-X----}(4/60) **BCD1950A**<br>(2/80){--X------}(6/60) **CDO1328**<br>(1/79){X---}(3/59) **opt-4891**
20	**ACO142B**	(5/59) {-----} = 5 cM

25	**ISU1450**	(14/62) {----------------} = 16 cM
41	**CDO1461**	(6/58) {------} = 6 cM<br>(6/54){-------X---}(3/50) **U71PM15**
47	**UMN509A**	(4/55) {----} = 4 cM
51	**UMN5271_2B**	(2/57) {--} = 2 cM<br>(7/49){--------X------}(5/50) **e5m5_10b**<br>(7/55){-------X------}(6/57) **e8m3_1b**<br>(3/57){---X----}(5/75) **opt-14277**<br>(3/55){---X}(0/73) **opt-1551**<br>(6/58){------X----}(5/76) **opt-17671**<br>(6/58){------X----}(6/77) **opt-17672**<br>(7/59){-------X----}(6/78) **opt-4047**<br>(7/59){-------X----}(6/77) **opt-4047_c**
53	**opt-17040**	(8/78) {------} = 6 cM<br>(21/76){--------------------X---------------------------}(25/76) **(Dup?)** **(>65%)(K+0+)opt-14301**<br>(21/76){--------------------X---------------------------}(25/76) **(Dup?)** **(>65%)(K+0+)opt-14301_c**
58	**opt-0136_c**	(2/63) {--} = 2 cM<br>(1/79){-X---}(3/62) **opt-0136**<br>(0/76){X--}(2/63) **opt-1312**<br>(0/79){X--}(2/63) **opt-13939**<br>(1/78){-X---}(3/61) **opt-16914**<br>(0/78){X--}(2/62) **opt-17378**<br>(0/80){X--}(2/63) **opt-17397**<br>(0/73){X--}(2/63) **opt-4481**<br>(0/77){X--}(2/62) **opt-4498**
60	**CDO304**	(2/60) {--} = 2 cM<br>(2/62){--X---}(3/59) **BCD135A**<br>(4/60){----X----}(4/57) **CDO1445B**<br>(1/61){-X--}(3/74) **opt-8116**<br>(1/56){-X-}(1/69) **AME010**
62	**opt-1362**	(3/60) {---} = 3 cM
64	**CDO673A**	(4/63) {---} = 3 cM<br>(3/60){---X-}(1/60) **Wacacac236**

68	**opt-0427_c**	(4/79) {---} = 3 cM<br>(9/56){----------X----------}(9/55) **(Seg)CDO414b**<br>(4/49){----X----}(4/49) **e6m5_14x**<br>(4/63){---X-----}(6/62) **re1m6_6x**<br>(2/57){--X--}(2/57) **re6m5_7x**<br>(4/62){----X---}(3/61) **UMN464B**<br>(0/80){X---}(4/79) **opt-0427**<br>(0/79){X---}(4/79) **opt-14501**<br>(2/71){-X-}(1/71) **AME196**<br>(4/57){----X---}(3/57) **M470.c**
71	**opt-5695_c**	(1/78) {-} = 1 cM<br>(2/67){--X--}(3/68) **CDO1473C**<br>(2/67){--X--}(3/68) **CDO1385C**<br>(14/60){----------------X-----------------}(15/61) **CDO1385E**<br>(1/62){-X--}(2/62) **ACO134BRI**<br>(3/59){---X---}(3/60) **BCD1049**<br>(2/62){--X---}(3/63) **CDO962B**<br>(6/59){------X-------}(7/60) **e2m2_14x**<br>(4/57){----X----}(4/58) **e4m2_8x**<br>(7/59){-------X------}(6/60) **e8m3_1d**<br>(4/54){----X-----}(5/55) **HKT1a**<br>(2/76){--X--}(4/76) **ISU707BRV**<br>(8/60){--------X-------}(7/61) **re1m3_1x**<br>(3/60){---X----}(5/61) **re1m3_9x**<br>(4/62){---X----}(5/63) **re3m7_6x**<br>(2/46){--X-}(1/46) **re8m1_8**<br>(13/73){-----------X-----------}(13/73) **UMN364B**<br>(2/62){--X---}(3/63) **UMN5497A**<br>(3/58){---X--}(2/59) **Wacacac483**<br>(2/53){--X-}(1/54) **WG420B**<br>(1/78){-X}(0/78) **opt-0114**<br>(0/78){X-}(1/78) **opt-0344**<br>(0/70){X-}(1/70) **opt-0713**<br>(0/78){X-}(1/78) **opt-0975**<br>(0/79){X-}(1/78) **opt-10721**<br>(1/76){-X-}(2/75) **opt-11606**<br>(0/79){X-}(1/78) **opt-13157**<br>(1/74){-X-}(1/74) **(Dup?)(>65%)opt-13271**<br>(2/75){-X}(0/75) **opt-14642**<br>(0/72){X-}(1/73) **opt-15006**<br>(1/79){-X}(0/78) **(Dup?)(>65%)opt-15145**<br>(0/77){X-}(1/77) **opt-1530**

		(0/76){X-}(1/76) **opt-15338**<br>(1/79){-X}(0/78) **(Dup?)(>65%)opt-16012**<br>(1/79){-X}(0/78) **(Dup?)(>65%)opt-16012_c**<br>(0/73){X}(0/72) **(Dup?)(>65%)opt-16462**<br>(0/77){X-}(1/77) **opt-16596**<br>(1/78){-X}(0/78) **(Dup?)(>65%)opt-16725**<br>(1/78){-X}(0/78) **(Dup?)(>65%)opt-16725_c**<br>(3/72){--X---}(4/72) **(Dup?)(>65%)opt-17171**<br>(3/72){--X---}(4/72) **(Dup?)(>65%)opt-17171_c**<br>(0/75){X-}(1/75) **opt-2626**<br>(1/78){-X}(0/78) **opt-3225**<br>(0/79){X-}(1/79) **opt-3909**<br>(1/78){-X-}(2/77) **(Dup?)(>65%)opt-4192**<br>(0/79){X-}(1/79) **opt-4542**<br>(0/77){X-}(1/77) **opt-5113**<br>(0/79){X-}(1/78) **opt-5695**<br>(2/70){-X--}(3/70) **(Dup?)(>65%)opt-5713**<br>(0/77){X-}(1/77) **opt-5883**<br>(7/72){-----X-----}(6/71) **(Dup?)(>65%)(K+0+)(ML?)opt-6974_rp**<br>(1/73){-X}(0/73) **opt-7105**<br>(1/73){-X}(0/73) **opt-7105_c**<br>(1/79){-X}(0/79) **opt-8960**<br>(0/79){X-}(1/79) **opt-9445**<br>(0/79){X-}(1/79) **opt-9445_c**<br>(0/76){X-}(1/75) **opt-9450**<br>(1/79){-X-}(2/79) **opt-9667**<br>(0/78){X-}(1/78) **opt-9667_c**<br>(3/60){---X----}(4/61) **M470.b**
71	**opt-8960_c**	(4/60) {---} = 3 cM<br>(8/63){-------X----}(4/60) **ISU2192A**<br>(4/57){----X----}(4/54) **UMN5489_2**
74	**BCD1280A**	(2/59) {-} = 1 cM<br>(1/58){-X--}(2/60) **ACO192CDI**<br>(4/55){---X----}(4/56) **re6m5_7bx**<br>(1/57){X--}(2/59) **Wacgcac265**<br>(2/59){-X--}(2/61) **L7M2.2c**<br>(2/59){-X---}(3/61) **L7M2.1**
76	**(Dup?)(ML?)opt-12959**	(3/69) {--} = 2 cM

78	**(Dup?)(>65%)opt-13235**	(13/69) {------------} = 12 cM<br>(19/53){-------------------------------X----------------}(14/59) **(Seg) Waaccac126**<br>(14/71){-------------X---}(4/78) **(Dup?)(>65%)opt-1225**<br>(14/70){-------------X---}(4/77) **(Dup?)(>65%)opt-15666**<br>(14/71){-------------X---}(4/78) **(Dup?)(>65%)opt-17867**<br>(14/71){-------------X---}(4/78) **(Dup?)(>65%)opt-17867_c**<br>(14/69){-------------X---}(4/76) **(Dup?)(>65%)opt-8576**
90	**(Dup?)(>65%)opt-10002**	(20/72) {--------------------} = 20 cM<br>(18/77){----------------X-------------------------------}(26/72) **(Dup?)(>65%)opt-5250**
110	**(Dup?)(>65%)opt-2102**	(6/69) {-----} = 5 cM<br>(1/71){-X-----------}(13/74) **(Dup?)(>65%)opt-5423**<br>(1/71){-X-----------}(13/74) **(Dup?)(>65%)opt-5423_c**<br>(0/70){X-----------}(13/74) **(Dup?)(>65%)opt-7308**
115	**BCD1643B**	(4/60) {----} = 4 cM<br>(2/61){--X----}(4/60) **BCD1797B**<br>(2/71){-X---}(3/56) **BCD808A**<br>(4/61){----X-}(2/60) **CDO122**<br>(8/62){-------X----}(4/60) **CDO348B**<br>(16/57){---------------------X--------------}(12/56) **e4m2_12x**<br>(5/58){-----X-------}(7/57) **phytoA**<br>(8/73){------X---}(3/59) **opt-10571**<br>(6/71){-----X--}(2/57) **opt-5847**<br>(7/74){-----X--}(2/59) **opt-5847_c**
118	**PGM**	(7/58) {-------} = 7 cM
125	**BCD1968A**	(7/65) {------} = 6 cM<br>(2/60){-X-------}(8/61) **BCD1797C**<br>(6/59){------X-----}(7/72) **opt-7599**
131	**ISU1961**	(10/62) {----------} = 10 cM
142	**CDO1168B**	(2/59) {-} = 1 cM<br>(4/62){---X--}(2/60) **BCD1842A**<br>(2/62){-X--}(2/59) **CDO457A**

143	**ACO138RI**	(3/60) {---} = 3 cM<br>(7/56){-------X----}(4/59) **re5m7_15x**<br>(2/60){--X-}(1/80) **opt-11700**<br>(4/57){----X---}(4/77) **opt-12717**<br>(2/60){--X-}(2/79) **opt-13630**<br>(3/59){---X}(0/79) **opt-13970**<br>(2/59){--X-}(1/79) **opt-4349**<br>(2/60){--X-}(2/80) **opt-4349_c**<br>(3/59){---X}(0/79) **opt-6549**
146	**opt-13970_c**	(12/77) {---------} = 9 cM
155	**opt-16018**	(2/71) {-} = 1 cM<br>(2/61){--X--}(2/58) **BCD1212**<br>(2/76){-X---}(4/73) **opt-9348**
156	**opt-3160**	(3/72) {--} = 2 cM<br>(2/56){--X-}(1/59) **BCD1153**<br>(3/57){---X--}(2/60) **BCD1186**<br>(4/71){---X}(0/75) **opt-3400**
159	**opt-17860**	(6/73) {----} = 4 cM<br>(4/75){---X----}(5/74) **CDO506A**<br>(10/69){---------X-------}(8/66) **(Dup?)(>65%)opt-11072**
163	**opt-3333**	(9/58) {---------} = 9 cM
172	**e2m2_1f**	

Go to top of this map

Linkage Group: 8

Total cM: 30

Framework Map		Placed markers (informative recombination fraction is shown in brackets)
**cM**	**Marker**	
0	**BCD1950B**	(10/58) {-----------} = 11 cM<br>(2/61){--X--------------}(12/56) **S5S2.2.11**
11	**BCD1102**	(3/56) {---} = 3 cM<br>(12/56){-------------X-------------}(12/58) **opt-14383_c_rp**<br>(11/54){-------------X------------}(11/56) **opt-17717_rp**

14	**Waaccac169**	(3/60) {---} = 3 cM<br>(9/54){----------X--------}(8/56) **e6m6_19x**<br>(8/59){--------X---------}(9/61) **re1m3_24x**<br>(4/56){----X-----}(5/56) **Waaccac641**
17	**(Dup?)(K+0+)opt-6825_c**	(3/75) {--} = 2 cM<br>(7/59){-------X-------}(7/60) **e4m6_7**<br>(6/60){------X-------}(7/61) **re1m3_18x**<br>(3/58){---X---}(3/59) **re1m6_11x**<br>(5/62){----X----}(5/63) **re6m6_1x**<br>(2/56){--X--}(2/57) **Wacacac242**<br>(0/71){X--}(3/72) **opt-0112**<br>(2/69){-X-----}(6/72) **(Dup?)(K+0+)opt-1611**<br>(0/73){X--}(3/74) **(Dup?)(K+0+)opt-6825**
19	**opt-10115**	(4/79) {---} = 3 cM<br>(5/80){---X---}(5/79) **BCD1160**<br>(8/68){-------X-------}(8/67) **CDO1436B**<br>(6/71){----X----}(6/70) **CDO780**<br>(4/71){---X--}(3/71) **opt-1232**<br>(1/78){-X---}(5/77) **opt-5045**<br>(1/78){-X---}(5/77) **opt-5045_c**<br>(4/70){---X----}(5/69) **AME162**
21	**opt-14889**	(1/79) {-} = 1 cM<br>(7/61){-------X------}(7/62) **BCD1307**<br>(3/59){---X---}(3/60) **Wacccac505**<br>(16/73){--------------X---------------}(17/74) **opt-14383_rp**
22	**opt-7254**	(8/59) {--------} = 8 cM
30	**Wacccac433**	

Go to top of this map

Linkage Group: 9

Total cM: 24

Framework Map		Placed markers (informative recombination fraction is shown in brackets)
**cM**	**Marker**	
0	**opt-4728**	(11/67) {----------} = 10 cM

10	**opt-17699**	(3/62) {---} = 3 cM<br>(5/59){-----X----}(4/56) **CDO1445A**<br>(3/62){---X}(0/63) **BCD1828**<br>(2/60){--X-}(1/57) **BCD1108**<br>(8/74){------X----------}(10/61) **opt-13852**<br>(0/68){X---}(3/57) **opt-4483**<br>(8/69){-------X-----}(5/57) **AME023b**
12	**BCD978**	(8/56) {--------} = 8 cM<br>(7/62){------X-----}(6/61) **BCD1237**<br>(5/56){-----X-------}(7/61) **BCD135B**<br>(6/55){------X-------}(7/60) **CDO1367**
21	**ACOR209A**	(4/62) {---} = 3 cM<br>(5/57){-----X-----}(6/58) **e6m5_1bx**
24	**CDO456**	

Go to top of this map

**Linkage Group: 11_41_20_45**<br><br>**Total cM: 106**		
**Framework Map**		**Placed markers**<br>**(informative recombination fraction is shown in brackets)**
**cM**	**Marker**	
0	**(Seg)UMN5254_2A**	(6/56) {------} = 6 cM
6	**(Dup?)(>65%)opt-15537_c**	(3/70) {--} = 2 cM<br>(0/70){X-}(1/68) **(Dup?)(>65%)(ML?)opt-12485**<br>(0/75){X--}(3/70) **(Dup?)(>65%)opt-15537**
		(3/73) {--} = 2 cM<br>(2/57){--X--}(2/59) **(Seg)BCD1270**<br>(3/59){---X---}(3/61) **(Seg)CDO1502**<br>(2/66){--X---}(4/69) **(Seg)CDO1509B**<br>(2/72){-X-}(2/76) **(Seg)CDO1414A**<br>(2/72){--X---}(4/76) **(Seg)Accase1**<br>(2/59){--X--}(2/61) **CDO572B**<br>(2/71){-X---}(4/75) **(Seg)CDO665B**<br>(2/57){--X-}(2/59) **(Seg)CDO783RV**<br>(1/56){-X-}(1/58) **(Seg)CSU36c**<br>(3/57){---X-----}(5/59) **e2m2_13x**<br>(3/52){---X----}(4/55) **(Seg)e8m8_6x**

8	**opt-3550**	(2/59){--X--}(2/61) **(Seg)ISU2287A**<br>(4/59){----X--}(2/61) **(Seg)pBCD175**<br>(2/57){--X--}(2/59) **(Seg)RZ69**<br>(1/53){-X-}(1/56) **(Seg)UAZ213c**<br>(2/55){-X}(1/56) **(Seg)UMN5032_1A**<br>(2/52){--X--}(2/55) **(Seg)Wacgcac612**<br>(1/70){-X--}(3/74) **opt-0473**<br>(1/74){-X--}(3/78) **opt-0473_c**<br>(16/73){--------------X-------------------}(20/76) **opt-11063**<br>(1/72){-X--}(3/76) **(Dup?)(ML?)opt-7687**<br>(1/73){-X--}(3/77) **opt-7854**<br>(10/59){----------X---------}(9/61) **ubc364Os**<br>(2/58){--X}(0/60) **(Seg)S3S1.24a**<br>(8/67){-------X-------}(8/69) **(Seg)AME054**<br>(8/67){-------X--------}(9/69) **cnl127**
10	**(Dup?)(>65%)opt-11634_c**	(3/75) {--} = 2 cM<br>(2/59){--X--}(2/58) **(Seg)CDO1479RI**<br>(5/58){-----X---}(3/56) **(Seg)re5m5_1**<br>(2/72){-X-}(2/70) **(Dup?)(>65%)opt-0096**<br>(0/75){X--}(3/72) **(Dup?)(>65%)opt-11634**<br>(0/78){X--}(3/75) **(Dup?)(>65%)opt-13168**<br>(3/77){--X-}(2/75) **(Dup?)(>65%)(ML?)opt-14299**<br>(3/77){--X-}(2/74) **(Dup?)(>65%)opt-1446**<br>(1/77){-X-}(2/74) **(Dup?)(>65%)opt-15784**<br>(2/74){-X-}(2/72) **(Dup?)(>65%)opt-17300**<br>(2/77){-X-}(1/74) **(Dup?)(>65%)(ML?)opt-18186**<br>(3/76){--X-}(2/73) **(Dup?)(>65%)opt-8495**<br>(3/70){--X--}(2/68) **AME019b**
12	**opt-6514**	(2/57) {--} = 2 cM<br>(2/52){--X}(0/50) **ACO164**<br>(6/52){-------X----}(4/54) **re6m4_1d**<br>(3/57){---X---}(3/59) **re6m6_5x**<br>(7/55){-------X-----}(5/57) **UAZ192**<br>(5/54){-----X--}(2/55) **UMN5192A**
14	**UMN5112_2**	(5/61) {----} = 4 cM<br>(4/58){----X----}(4/60) **CDO1281**<br>(6/55){------X-----}(5/57) **PKABA1**

19	**(Seg)CDO1436C**	(4/68) {---} = 3 cM<br>(6/68){----X-----}(7/78) **opt-1032**<br>(4/69){---X-}(1/79) **opt-12680**<br>(4/66){---X-}(1/75) **opt-13153**<br>(3/66){--X}(0/77) **opt-13497**<br>(3/66){--X}(0/77) **opt-13620**<br>(4/69){---X-}(1/79) **(Dup?)(>65%)opt-1568**<br>(5/67){----X----}(6/77) **(Dup?)(>65%)opt-2998**<br>(4/66){----X}(0/75) **(Dup?)(>65%)opt-4069**<br>(3/68){--X}(0/78) **opt-6877**
22	**opt-6877_c**	(8/79) {------} = 6 cM<br>(7/60){-------X---}(3/61) **CDO1464**<br>(6/53){------X----}(4/54) **re2m5_6x**<br>(7/77){-----X}(0/78) **opt-10163**<br>(7/77){-----X}(0/78) **opt-17524**
27	**opt-10163_c**	(12/61) {------------} = 12 cM<br>(13/65){-------------X-}(1/57) **BCD880A**<br>(21/59){------------------------------X---------------}(13/57) **(Seg) Wacacaa131**
40	**CDO836ARV**	(13/61) {--------------} = 14 cM
54	**opt-10660_c**	(4/78) {---} = 3 cM<br>(8/61){-------X---}(4/60) **OG19**<br>(5/59){-----X-}(1/58) **Wacacaa179**<br>(5/60){-----X-}(1/59) **Wacgcaa1089**<br>(15/74){-------------X-----------------}(18/73) **(Dup?)(>65%)(K+0+) opt-0026_rp**<br>(0/76){X---}(4/74) **opt-10660**<br>(0/79){X---}(4/78) **opt-15681**<br>(0/77){X---}(4/76) **opt-16298**<br>(11/72){---------X-----}(7/71) **(Dup?)(>65%)opt-1644**<br>(0/77){X---}(4/76) **opt-2561**<br>(5/75){----X}(0/73) **opt-4063**<br>(4/77){---X}(0/77) **opt-6501**<br>(4/72){---X------}(7/70) **(Dup?)(K+0+)opt-9645_rp**<br>(17/77){---------------X------------------}(19/75) **(Dup?)(>65%)opt-17375_rp**
56	**opt-12686**	(16/74) {--------------} = 14 cM<br>(9/59){---------X----------}(10/60) **UMN5960**

70	**CDO1090C**	(4/74) {---} = 3 cM<br>(2/62){--X-----}(6/62) **BCD1502**<br>(0/59){X----}(4/58) **BCD1632B**<br>(0/76){X---}(4/74) **CDO203A**<br>(6/63){-----X--------}(8/62) **BCD1716B**<br>(6/61){------X-------}(8/60) **BCD1931**<br>(4/49){----X------}(5/48) **BCD1959**<br>(10/60){-----------X--------------}(12/59) **(Seg)BCD421A**<br>(4/62){---X-------}(8/63) **ACO245B**<br>(0/76){X---}(4/74) **CDO57B**<br>(6/60){------X--------}(8/59) **ISU1543A**<br>(7/58){-------X------}(6/57) **re5m5_15x**<br>(9/75){-------X----------}(13/77) **UMN364A**<br>(6/61){-----X----------}(10/60) **UMN5110**
73	**opt-14264**	(3/72) {--} = 2 cM<br>(3/78){--X}(0/73) **opt-16610**<br>(2/69){-X---}(4/64) **(Dup?)(K+0+)opt-7041_rp**<br>(0/75){X--}(3/72) **opt-9850**<br>(5/54){-----X--}(2/50) **S5S1.1.6**
75	**opt-16202_c**	(2/71) {-} = 1 cM<br>(0/73){X-}(2/71) **opt-16202**
77	**opt-7857**	(3/78) {--} = 2 cM<br>(8/62){--------X--------}(8/62) **CDO1428A**
79	**opt-8774**	(2/79) {-} = 1 cM<br>(9/72){-------X------}(8/72) **AM42**<br>(10/58){-----------X-----------}(10/58) **e5m5_21x**<br>(6/54){------X------}(6/54) **e6m5_10x**<br>(5/61){----X----}(5/61) **re1m3_12x**<br>(3/78){--X-}(2/78) **opt-12729**<br>(4/77){---X--}(3/78) **opt-13969**<br>(3/77){--X-}(2/78) **opt-3049**<br>(3/76){--X-}(2/77) **opt-3049_c**
80	**opt-17088**	(2/70) {-} = 1 cM<br>(9/62){---------X-----}(4/54) **BCD1826**<br>(8/62){-------X----}(4/54) **BCD1860**<br>(10/54){------------X-------}(6/48) **CSU100**<br>(5/61){----X--}(2/53) **re3m7_1bx**<br>(12/62){------------X---------}(8/54) **re6m4_21x**<br>(23/77){-----------------------X----------------}(16/68) **(Dup?)(>65%) opt-2890**

82	**opt-15363**	(2/60) {--} = 2 cM
83	**opt-10664**	(1/63) {-} = 1 cM<br>(4/46){-----X---------}(8/53) **e8m3_12x**<br>(2/50){--X---}(3/55) **ubc375s_DdeI**
84	**opt-14292**	(15/74) {-------------} = 13 cM
97	**opt-8509_c**	(9/63) {--------} = 8 cM<br>(0/59){X--------}(7/54) **e3m7_19x**<br>(7/60){-------X}(0/55) **Wacgcaa368**<br>(0/74){X---------}(9/59) **(Dup?)(ML?)opt-1518**<br>(0/79){X--------}(9/63) **opt-8509**
106	**BCD1882A**	

Go to top of this map

Linkage Group: 14

Total cM: 55

**Framework Map**		**Placed markers (informative recombination fraction is shown in brackets)**
**cM**	**Marker**	
0	**e1m3_10x**	(7/57) {-------} = 7 cM<br>(14/61){---------------X--------------------}(17/59) **UMN5101_3B**<br>(13/53){-----------------X------------------}(17/67) **(Seg)AME159**
7	**CDO1090E**	(7/72) {-----} = 5 cM<br>(0/74){X-----}(7/72) **CDO203D**<br>(0/74){X-----}(7/72) **CDO57D**<br>(4/48){-----X---}(3/50) **e1m2_7x**<br>(5/47){------X-------}(6/48) **e5m3_5**<br>(4/73){---X--------}(10/77) **opt-0679**<br>(5/69){----X--}(3/73) **opt-11850**<br>(6/73){----X--}(3/77) **opt-16366**<br>(1/69){-X----}(5/71) **opt-17644**<br>(1/70){-X----}(6/73) **opt-2151**<br>(4/67){---X---------}(10/69) **opt-4076**<br>(4/74){---X-------}(10/78) **opt-4076_c**<br>(6/70){-----X}(0/74) **opt-5357**<br>(0/68){X----}(5/71) **opt-5506**<br>(0/64){X-----}(6/66) **(Dup?)(K+0+)opt-7651**<br>(0/72){X-----}(7/76) **opt-9137**<br>(0/73){X----}(6/77) **opt-9137_c**

12	**opt-11595_c**	(2/62) {--} = 2 cM<br>(1/57){-X--}(2/58) **CSU43b**<br>(7/59){-------X------}(6/60) **CSU43d**<br>(8/60){--------X----------}(10/61) **e6m6_8x**<br>(2/55){--X--}(2/56) **ISU1540**<br>(0/73){X--}(2/60) **opt-10221**<br>(1/76){-X--}(2/62) **opt-10879**<br>(1/77){-X--}(2/63) **opt-10879_c**<br>(5/76){----X---}(4/62) **opt-10910**<br>(6/78){----X----}(5/63) **opt-10910_c**<br>(5/76){----X---}(3/61) **opt-11076**<br>(0/78){X--}(2/62) **opt-11595**<br>(0/76){X--}(2/61) **opt-12360**<br>(0/76){X--}(2/62) **opt-12862**<br>(8/75){------X----}(5/61) **opt-1512**<br>(8/76){------X----}(5/62) **opt-15496**<br>(0/75){X--}(2/61) **opt-1558**<br>(0/78){X--}(2/63) **opt-1558_c**<br>(7/76){-----X----}(5/63) **opt-4003**<br>(8/77){------X----}(5/63) **opt-4898**<br>(1/78){-X---}(3/63) **opt-5036**<br>(1/78){-X--}(2/63) **opt-6964**<br>(1/75){-X--}(2/61) **opt-7259**<br>(7/74){-----X---}(3/60) **opt-8984**<br>(8/76){------X----}(5/63) **opt-9025**
14	**UMN339A**	(5/63) {----} = 4 cM
18	**CDO400**	(5/75) {----} = 4 cM<br>(0/63){X---}(4/63) **WG719A**<br>(7/75){-----X----}(5/71) **opt-11549**<br>(9/79){------X----}(6/75) **opt-2638**<br>(11/62){-----------X--------------}(13/62) **II2.3a**
22	**BCD1184**	(3/70) {--} = 2 cM<br>(2/63){--X---}(3/59) **CDO1495**<br>(2/63){--X--}(2/59) **CDO1359**<br>(3/75){--X----}(5/73) **CDO64**<br>(8/74){------X-------}(9/73) **opt-5020**<br>(8/75){------X-------}(9/74) **opt-9025_c**<br>(5/63){----X-----}(5/59) **ubc109As**

24	**opt-12121**	(2/69) {-} = 1 cM<br>(2/43){--X--}(2/42) **BCD1968D**<br>(3/67){--X--}(3/69) **opt-16924**<br>(16/67){----------------X---------------}(15/67) **(Seg)AME137**
26	**opt-13557**	(8/70) {-------} = 7 cM
33	**BCD1729**	(6/64) {-----} = 5 cM<br>(2/62){-X----}(4/59) **CDO1430**<br>(1/57){-X-----}(4/54) **BCD1842C**<br>(7/66){------X}(0/59) **CDO665A**
37	**CDO1509A**	(6/54) {------} = 6 cM
44	**UMN5353A**	(2/55) {-} = 1 cM
45	**CDO1502BRV**	(2/60) {-} = 1 cM<br>(4/57){----X------}(6/58) **CSU46**<br>(4/54){---X----}(4/55) **re8m8_3**<br>(1/58){X--}(2/59) **Wacacac460**<br>(4/54){-----X-----}(6/68) **opt-4175**
46	**opt-0760**	(10/70) {--------} = 8 cM<br>(6/38){---------X}(0/34) **(Seg)CDO1414D**<br>(16/60){-------------------X---------}(8/55) **ISU2287BDI**<br>(19/72){------------------X--------}(9/67) **opt-3538_rp**
55	**opt-8928**	

Go to top of this map

Linkage Group: 15

Total cM: 88

**Framework Map**		**Placed markers (informative recombination fraction is shown in brackets)**
**cM**	**Marker**	
0	**BCD1823B**	(3/62) {---} = 3 cM<br>(1/62){-X---}(3/63) **CDO1345**
3	**opt-4587**	(4/79) {---} = 3 cM<br>(2/74){--X---}(4/73) **ACO226A**<br>(3/78){--X}(0/78) **opt-16444**<br>(4/77){---X}(0/77) **opt-6975**
5	**opt-6975_c**	(9/78) {-------} = 7 cM

12	**(Dup?)(ML?)opt-10153**	(3/79) {--} = 2 cM<br>(3/61){---X---}(3/62) **CDO1403B**<br>(2/75){-X}(0/75) **opt-12331**<br>(5/75){----X-}(2/76) **opt-14868**<br>(2/75){-X----}(5/76) **opt-1583_rp**<br>(2/76){-X}(0/77) **opt-15938**<br>(1/77){-X-}(1/78) **opt-17714**<br>(1/76){-X-}(1/77) **opt-17746**<br>(2/76){-X-}(2/77) **opt-1789**<br>(2/76){-X--}(3/77) **opt-18251**<br>(3/77){--X-}(2/78) **opt-2748**<br>(3/75){--X}(0/76) **opt-4815**<br>(1/75){-X-}(2/76) **opt-8239**<br>(3/76){--X-}(1/77) **opt-8678**
14	**opt-15938_c**	(7/77) {-----} = 5 cM
19	**CDO460B**	(11/76) {---------} = 9 cM
27	**opt-14840_c**	(10/78) {-------} = 7 cM<br>(5/48){------X-----}(4/48) **e8m3_11**<br>(0/77){X--------}(10/76) **opt-14050**<br>(0/77){X-------}(9/76) **opt-14840**<br>(2/77){-X--------}(10/77) **opt-2439**<br>(3/76){--X--------}(10/76) **(Dup?)(K+0+)opt-4234_rp**<br>(0/74){X------}(8/75) **opt-6926**
35	**opt-8866**	(2/55) {--} = 2 cM<br>(0/68){X--}(2/56) **CDO1435C**<br>(11/56){------------X------------}(10/50) **e1m6_20x**<br>(5/52){-----X---}(2/47) **e8m3_4x**<br>(13/57){---------------X-------------------}(14/51) **(Seg)re1m6_13**<br>(12/59){-------------X----------}(8/53) **re2m2_6**<br>(2/59){--X----}(4/55) **Wacccac609**<br>(3/78){--X}(1/55) **opt-11190**<br>(3/78){--X}(1/55) **opt-11190_c**<br>(10/78){-------X---}(4/55) **opt-15557**<br>(8/77){------X--}(2/55) **opt-15592**<br>(10/79){-------X---}(4/56) **opt-15592_c**
37	**UMN5214_3**	(10/56) {----------} = 10 cM

47	**opt-11075_c**	(3/79) {--} = 2 cM<br>(4/78){---X--}(2/77) **BCD115C**<br>(10/61){----------X-------}(7/61) **e1m3_17x**<br>(5/46){------X------}(5/46) **e8m1_8x**<br>(7/63){------X-------}(8/63) **UMN128**<br>(0/79){X-}(2/78) **opt-11075**<br>(0/78){X-}(2/77) **opt-13250**<br>(9/80){------X-------}(10/79) **opt-13953**<br>(9/80){------X-------}(10/79) **opt-13953_c**<br>(3/79){--X}(0/78) **opt-14513**<br>(2/78){-X---}(5/78) **opt-17109**<br>(8/79){------X-------}(10/78) **opt-17754**<br>(9/79){------X-------}(9/78) **opt-2477**<br>(9/79){------X-------}(9/78) **(Dup?)(ML?)opt-2523**<br>(1/74){-X-}(1/74) **opt-9407**<br>(9/80){------X-------}(10/79) **opt-9487**<br>(9/71){-------X-------}(9/71) **AME077**
49	**opt-8925**	(8/68) {------} = 6 cM<br>(7/79){-----X---}(4/69) **CDO54**<br>(5/58){-----X-------}(8/58) **e4m2_11x**
56	**CDO1385A**	(2/68) {--} = 2 cM<br>(2/68){--X-}(2/78) **BCD127**<br>(2/64){-X----}(5/73) **BCD1380A**<br>(2/62){-X-}(1/61) **BCD1819A**<br>(2/57){--X--}(2/56) **CDO118RI**<br>(2/61){--X--}(2/63) **ACO176**<br>(6/62){------X-----}(6/61) **e2m2_1e**<br>(4/55){---X---}(3/54) **e2m5_2**<br>(4/59){---X----}(4/58) **e4m2_4x**<br>(2/56){-X--}(2/55) **e5m5_8**<br>(0/55){X-}(1/54) **e6m6_19**<br>(2/61){--X---}(3/61) **IDH_1**<br>(1/55){X-}(1/54) **re5m5_7**<br>(6/62){------X--------}(8/61) **re6m6_2x**<br>(2/68){-X---}(4/78) **UMN44**<br>(12/63){-------------X---------------}(14/62) **UMN5101_3A**<br>(3/53){---X----}(4/52) **UMN5310**<br>(2/60){--X-}(1/59) **Waaccac183**

58	**opt-17391_c**	(3/74) {--} = 2 cM<br>(2/77){-X-}(2/72) **UMN430**<br>(0/77){X--}(3/72) **opt-0138**<br>(0/77){X-}(2/72) **opt-17391**<br>(1/77){-X-}(2/72) **opt-3221**<br>(1/79){-X-}(2/74) **opt-3221_c**<br>(1/78){-X-}(2/73) **opt-4061**<br>(1/73){-X-}(2/71) **opt-4850**<br>(1/75){-X-}(1/72) **opt-5351**<br>(1/76){-X-}(2/71) **opt-6279**<br>(1/72){-X-}(2/70) **opt-7031**<br>(1/78){-X-}(2/73) **opt-8827**<br>(1/79){-X-}(2/74) **opt-8976**
60	**opt-5213**	(5/73) {----} = 4 cM<br>(4/58){----X--}(2/60) **BCD1145**<br>(5/59){-----X---}(3/62) **CDO585A**<br>(3/45){----X--}(2/48) **e5m3_2a**<br>(10/50){-------------X------------}(10/54) **UMN5276_3**
63	**BCD1127A**	(13/61) {--------------} = 14 cM
77	**e4m6_13x**	(11/62) {-----------} = 11 cM
88	**opt-12302**	

Go to top of this map

Linkage Group: 16_23

Total cM: 114

**Framework Map**		**Placed markers (informative recombination fraction is shown in brackets)**
**cM**	**Marker**	
0	**ISU582B**	(4/62) {---} = 3 cM<br>(2/52){---X----}(4/52) **ACO237DI**<br>(2/57){--X--}(2/57) **ACOR254B**

3	**opt-17274**	(2/76) {-} = 1 cM<br>(3/72){--X---}(4/72) **CDO1508B**<br>(2/76){-X}(0/77) **opt-1185**<br>(2/76){-X}(0/78) **(Dup?)(ML?)opt-12925**<br>(2/76){-X-}(1/78) **opt-13480**<br>(2/76){-X}(0/77) **(Dup?)(ML?)opt-13547**<br>(3/77){--X}(0/78) **opt-14675**<br>(4/77){---X}(0/77) **opt-17564**<br>(2/76){-X}(0/78) **opt-3413**<br>(2/77){-X-}(1/78) **opt-3804**<br>(2/77){-X-}(1/78) **opt-3804_c**<br>(2/76){-X}(0/78) **opt-5738**<br>(2/74){-X-}(1/74) **opt-9107**
5	**opt-5738_c**	(2/61) {--} = 2 cM
7	**CDO1402B**	(11/61) {-----------} = 11 cM
18	**opt-16084**	(1/79) {-} = 1 cM<br>(1/78){-X}(0/79) **opt-16130**<br>(1/78){-X}(0/79) **opt-17966**<br>(1/77){-X-}(1/78) **opt-2798**<br>(1/79){-X}(0/79) **opt-4643**<br>(0/78){X}(0/79) **opt-7690**
19	**opt-16130_c**	(3/78) {--} = 2 cM<br>(8/49){----------X--------}(7/49) **ISU1795**<br>(3/78){--X}(0/76) **(Dup?)(ML?)opt-11481**<br>(2/79){-X-}(1/77) **(Dup?)(ML?)opt-11481_c**
21	**opt-6090**	(9/56) {----------} = 10 cM<br>(12/74){----------X----}(4/56) **CDO58C**<br>(11/60){-----------X-------}(7/57) **ISU563A**
30	**UMN5655**	(8/53) {---------} = 9 cM<br>(4/58){----X-------}(7/58) **UMN339B**<br>(6/54){------X---------}(8/55) **UMN5419**

39	**opt-18371**	(4/71) {---} = 3 cM<br>(2/54){--X--}(2/58) **BCD1632C**<br>(4/55){---X---}(4/59) **BCD385**<br>(4/55){----X---}(3/59) **CDO1192A**<br>(2/57){--X--}(2/61) **CDO1416**<br>(4/55){----X---}(3/59) **BCD1126**<br>(2/56){--X--}(2/60) **BCD1088**<br>(6/50){-------X-----}(5/53) **CSU154b**<br>(4/51){----X----}(4/54) **CSU43a**<br>(5/58){-----X---}(4/62) **e6m4_1**<br>(4/54){----X----}(4/58) **re2m2_7**<br>(4/39){------X-----}(4/42) **re2m3_12a**<br>(7/48){---------X---------}(8/52) **U8PM26**
42	**opt-18282**	(2/71) {-} = 1 cM<br>(3/60){---X---}(3/55) **BCD1544**<br>(4/73){---X---}(4/70) **CDO203C**<br>(4/73){---X---}(4/70) **CDO1090D**<br>(1/69){-X--}(2/65) **ACO182A**<br>(1/69){-X--}(2/65) **CDO358BRI**<br>(0/60){X--}(2/57) **CDO412B**<br>(4/73){---X---}(4/70) **CDO57C**<br>(4/56){----X-----}(5/54) **CSU43c**<br>(2/60){--X----}(4/56) **e1m3_3**<br>(7/62){------X--------}(8/58) **e2m3_5**<br>(1/74){-X-}(1/69) **opt-10878**<br>(1/77){-X-}(1/72) **opt-10878_c**<br>(1/77){-X-}(1/72) **opt-12553**<br>(1/74){-X-}(2/71) **opt-13092**<br>(2/73){-X--}(2/68) **opt-16131**<br>(13/74){-----------X----------}(12/70) **(Dup?)(K+0+)opt-17318_rp**<br>(1/72){-X-}(2/69) **opt-17903**<br>(1/75){-X-}(2/71) **opt-17903_c**<br>(2/76){-X-}(2/71) **opt-2635**<br>(2/76){-X--}(3/72) **opt-2635_c**<br>(1/76){-X-}(2/72) **opt-4385**<br>(1/72){-X-}(1/67) **opt-4671**<br>(12/74){----------X----------}(12/71) **(Dup?)(K+0+)opt-5121_rp**<br>(1/76){-X-}(1/70) **opt-5427**<br>(1/77){-X-}(1/73) **opt-5729**<br>(1/77){-X-}(2/72) **opt-8809**<br>(1/77){-X-}(1/73) **opt-9329**<br>(1/74){-X-}(1/69) **opt-9496**

		(3/66){--X---}(3/63) **AME154**<br>(5/69){----X----}(5/65) **AME062**<br>(5/70){----X------}(7/66) **AME048**
44	**opt-14781**	(16/72) {---------------} = 15 cM
59	**opt-17022_c**	(8/59) {-------} = 7 cM<br>(5/77){---X------}(6/58) **opt-11575**<br>(5/75){----X-----}(6/59) **opt-13249**<br>(4/78){---X------}(6/59) **opt-15119**<br>(0/76){X-------}(8/58) **opt-15436**<br>(0/76){X---------}(8/57) **opt-17022**<br>(5/72){----X-------}(6/54) **opt-3345**<br>(5/79){---X------}(6/60) **opt-3345_c**
66	**BCD709**	(3/60) {---} = 3 cM
69	**CDO1360**	(2/63) {-} = 1 cM<br>(2/56){--X-}(1/56) **BCD1430**<br>(9/78){-------X-----}(6/62) **CDO1509C**<br>(6/61){-----X---}(3/47) **CDO665C**<br>(6/55){------X-------}(7/55) **CSU36d**<br>(4/58){----X----}(4/58) **CSU36e**<br>(6/57){-----X------}(6/57) **UAZ213d**<br>(4/59){----X-----}(5/59) **UAZ213e**<br>(6/78){----X--}(2/63) **WG466**<br>(4/76){---X---}(3/60) **opt-0373**<br>(4/73){---X--}(2/59) **opt-10150**<br>(6/76){----X----}(4/61) **opt-4457**<br>(6/77){----X----}(4/61) **opt-4457_c**<br>(4/69){---X--}(2/55) **(Dup?)(ML?)opt-7342**
70	**BCD1695**	(14/61) {----------------} = 16 cM<br>(10/40){-----------------X---------}(6/39) **CDO586A**<br>(11/60){-----------X-----------}(10/58) **Waaccac96**
86	**BCD1265**	(8/57) {---------} = 9 cM<br>(2/60){-X----------}(12/73) **BCD110**<br>(10/59){-----------X------}(6/57) **e1m3_12n**<br>(10/48){-------------X-------------}(10/48) **e8m1_4**<br>(1/54){-X---------}(10/67) **AME184**
95	**UMN13**	(11/75) {---------} = 9 cM

103	**opt-13565_c**	(6/78) {----} = 4 cM<br>(8/68){-------X---}(4/66) **CDO1092**<br>(6/63){-----X----------}(10/62) **BCD1117**<br>(0/79){X---}(5/77) **opt-13565**<br>(0/80){X----}(6/78) **opt-15462**<br>(2/72){-X------}(7/70) **opt-16883**<br>(0/79){X---}(5/77) **opt-17321**<br>(0/77){X----}(6/75) **opt-6367**<br>(0/79){X---}(5/77) **opt-7639**
108	**opt-5834**	(7/61) {-------} = 7 cM<br>(12/58){-------------X---------}(9/57) **PDS2**<br>(9/76){-------X----}(5/61) **UMN5904A**<br>(10/57){-----------X------}(6/56) **UMN5990_2**<br>(3/59){---X------}(6/60) **Wacgcac416**<br>(7/68){------X-----}(5/58) **opt-7304**
114	**re4m6_2x**	

Go to top of this map

Linkage Group: 17

Total cM: 72

Framework Map		Placed markers (informative recombination fraction is shown in brackets)
**cM**	**Marker**	
0	**opt-13800_c**	(21/70) {-----------------------} = 23 cM<br>(4/61){----X-------------------------}(18/57) **CDO1527B**<br>(0/76){X-----------------------}(20/67) **opt-13638**<br>(0/77){X--------------------}(19/68) **opt-13800**<br>(4/75){---X----------------------------}(23/68) **opt-6432**
23	**(Dup?)(>65%)opt-15391**	(8/67) {-------} = 7 cM<br>(20/66){-----------------------X-------------------}(18/68) **(Seg)AM7**<br>(7/46){---------X-----------------------}(14/47) **(Seg)HKT1d**<br>(3/52){---X-----------}(9/52) **(Seg)Wacgcaa137**<br>(0/63){X------}(7/65) **(Dup?)(>65%)opt-0732**<br>(6/69){-----X---------------}(16/70) **(Dup?)(>65%)opt-6668**

30	**opt-3019**	(4/67) {---} = 3 cM<br>(2/58){--X----}(4/56) **BCD1280B**<br>(1/47){-X-----}(4/45) **e1m2_6**<br>(6/57){------X-------}(7/56) **e6m6_3x**<br>(4/53){----X------}(5/50) **e8m3_17x**<br>(3/55){---X-----}(5/53) **Waaccac376**<br>(1/56){-X-----}(5/54) **Wacgcac225**<br>(1/73){-X--}(3/71) **opt-11552**<br>(1/72){-X--}(3/70) **opt-13793**<br>(5/63){----X---------}(9/62) **(Dup?)(K+0+)opt-13965_rp**
33	**opt-11469**	(4/71) {---} = 3 cM<br>(6/54){------X------}(6/56) **UMN5078**
36	**ISU707CRV**	(2/74) {--} = 2 cM<br>(1/59){X-}(2/60) **CDO341**<br>(2/59){--X----}(4/60) **ACO135RV**<br>(2/54){--X---}(4/55) **ACO192ADI**<br>(0/59){X---}(3/62) **CDO608B**<br>(2/59){--X---}(4/62) **CDO673B**<br>(0/59){X--}(2/62) **CDO962A**<br>(3/56){---X------}(6/59) **CSU154c**<br>(4/57){----X------}(6/60) **e1m3_10**<br>(5/57){-----X-------}(7/60) **e1m3_14a**<br>(3/57){---X-----}(5/60) **e1m3_14x**<br>(1/53){-X---}(3/56) **e4m6_19x**<br>(4/53){----X-------}(7/56) **e5m5_3**<br>(0/58){X--}(2/61) **HKT1b**<br>(5/56){-----X-----}(5/59) **re1m3_16**<br>(4/50){----X----}(4/53) **re2m5_6**<br>(3/51){---X-----}(5/54) **re8m3_14**<br>(3/52){---X------}(6/55) **re8m3_3x**<br>(2/55){--X--}(2/57) **UMN370**<br>(3/58){---X---}(3/61) **UMN464A**<br>(12/59){------------X-------------}(13/62) **UMN5101_3C**<br>(4/57){----X---}(3/60) **UMN5649**<br>(2/55){--X---}(3/58) **Waaccac195**<br>(2/56){--X----}(4/59) **Wacacaa453**<br>(7/52){-------X---------}(9/55) **Wacacaa790**<br>(0/57){X--}(2/60) **Wacgcaa218**<br>(0/56){X--}(2/59) **Wacgcac280**<br>(0/56){X--}(2/59) **Wacgcac87**<br>(7/44){---------X---------------}(11/45) **waxy**<br>(2/55){--X----}(4/58) **WG420A**

		(2/73){-X--}(3/77) **opt-3314**<br>(3/58){---X----}(4/61) **L7M2.2a**<br>(1/56){-X-}(1/60) **M470.a**
38	**opt-8219_c**	(2/77) {-} = 1 cM<br>(5/62){----X---}(3/62) **CDO1467A**<br>(6/78){----X----}(6/77) **CDO1340**<br>(8/58){--------X------}(6/58) **CDO1378A**<br>(4/60){---X}(1/60) **ACO193ERV**<br>(4/58){----X---}(3/59) **ACOR221**<br>(4/61){----X---}(3/61) **Acp1**<br>(4/58){----X---}(3/58) **Acp2**<br>(4/51){----X--}(2/51) **e1m2_3a**<br>(6/61){-----X----}(4/61) **e4m6_4**<br>(10/60){----------X--------}(8/59) **e5m7_7**<br>(5/62){----X---}(3/62) **ISU1146BDI**<br>(12/62){------------X-----------}(11/62) **ISU1364A**<br>(11/52){--------------X-----------}(9/52) **re5m5_12x**<br>(8/50){----------X-------------}(10/50) **(Seg)re8m1_1x**<br>(11/58){------------X-------------}(12/58) **(Seg)UMN5497**<br>(11/70){---------X-------}(8/69) **UMN97A**<br>(4/60){----X----}(4/60) **Waaccac89**<br>(1/53){-X-}(1/53) **Waagcac95**<br>(2/60){--X-}(1/59) **Wacgcaa152**<br>(3/77){--X}(0/77) **opt-10224**<br>(5/79){---X-}(1/78) **opt-10224_c**<br>(2/76){-X}(0/76) **opt-10683**<br>(2/77){-X-}(2/76) **opt-11249**<br>(2/78){-X-}(2/77) **opt-11249_c**<br>(2/77){-X-}(2/76) **opt-11468**<br>(2/77){-X-}(2/76) **opt-11705**<br>(0/73){X-}(1/72) **opt-12024**<br>(0/75){X-}(1/74) **opt-12932**<br>(0/75){X-}(2/75) **opt-13367**<br>(2/77){-X}(0/78) **opt-13870**<br>(2/76){-X-}(2/75) **opt-14589**<br>(0/76){X-}(1/74) **opt-14899**<br>(2/74){-X-}(2/74) **(Dup?)(ML?)opt-15097**<br>(4/78){---X}(0/77) **opt-1586**<br>(3/77){--X--}(3/76) **opt-16384**<br>(0/75){X-}(2/75) **opt-16821**<br>(4/68){---X-}(1/67) **opt-1724**<br>(3/78){--X--}(3/77) **opt-1729**

		(0/76){X-}(1/74) **opt-1769**<br>(0/74){X-}(1/73) **opt-3184**<br>(4/79){---X---}(4/78) **opt-3314_c**<br>(2/76){-X-}(2/75) **(Dup?)(ML?)opt-4358**<br>(0/78){X-}(2/76) **opt-4598**<br>(2/74){-X-}(2/73) **opt-5825**<br>(3/77){--X--}(3/76) **opt-6175**<br>(4/78){---X}(0/77) **opt-6278**<br>(0/77){X-}(1/75) **opt-7483**<br>(0/71){X-}(1/70) **opt-7512**<br>(0/71){X-}(1/70) **opt-7512_c**<br>(2/76){-X-}(2/75) **opt-7570**<br>(0/77){X-}(2/76) **opt-7724**<br>(0/79){X-}(2/78) **opt-7724_c**<br>(0/76){X-}(2/75) **opt-8219**<br>(6/62){-----X-------}(8/62) **ubc264KAs**<br>(1/52){-X--}(2/52) **U8PM1**<br>(3/61){---X-}(2/61) **III2.18b**
39	**opt-10683_c**	(2/76) {-} = 1 cM<br>(11/50){--------------X--------------}(11/50) **e1m2_6x**<br>(6/52){-------X----}(4/52) **e4m2_11ax**<br>(3/77){--X}(0/76) **opt-10382**<br>(3/77){--X}(0/76) **opt-10382_c**<br>(2/76){-X-}(1/76) **opt-12866**<br>(2/76){-X}(0/77) **opt-14345**<br>(2/71){-X}(0/71) **(Dup?)(>65%)opt-15198**<br>(6/76){----X----}(6/76) **opt-16029**<br>(2/77){-X-}(1/76) **opt-16117**<br>(3/77){--X}(0/77) **opt-16821_c**<br>(2/77){-X-}(1/76) **opt-16836**<br>(17/72){----------------X------------}(14/73) **(Dup?)(>65%)opt-17178**<br>(17/72){----------------X------------}(14/73) **(Dup?)(>65%)opt-17178_c**<br>(4/71){---X--}(3/70) **opt-17296**<br>(3/74){--X---}(4/75) **opt-3494**<br>(3/76){--X-}(1/76) **opt-3797**<br>(3/74){--X}(0/73) **(Dup?)(ML?)opt-6316**<br>(2/76){-X}(0/77) **opt-6327**

41	**opt-14345_c**	(5/77) {---} = 3 cM<br>(4/62){---X-}(1/62) **BCD1338B**<br>(4/61){----X-}(1/61) **BCD1851B**<br>(4/61){----X--}(2/62) **BCD421C**<br>(6/77){----X--}(3/78) **CDO1199B**<br>(4/60){---X-}(2/60) **ACO123**<br>(4/62){---X-}(1/62) **BCD1124**<br>(4/60){---X-}(2/60) **CDO358EDI**<br>(8/75){------X----}(5/76) **CDO53**<br>(1/38){-X}(0/39) **CDO539C**<br>(6/61){-----X---}(3/61) **CDO99**<br>(10/60){----------X---------}(9/60) **e1m3_11**<br>(6/59){------X---}(3/59) **e2m2_9x**<br>(6/62){-----X---}(3/62) **e3m7_5x**<br>(5/62){----X--}(2/62) **e3m7_7x**<br>(7/61){-------X----}(4/61) **e4m6_1a**<br>(4/58){----X-}(1/59) **e6m6_14a**<br>(6/59){------X---}(3/59) **e6m6_7**<br>(9/61){---------X-----}(6/61) **ISU1755B**<br>(4/49){----X----}(4/49) **re2m5_8a**<br>(12/57){--------------X---------}(9/57) **re3m7_20x**<br>(4/62){---X--}(2/62) **re3m7_4x**<br>(6/57){------X-----}(5/57) **re5m5_10**<br>(6/59){------X---}(3/60) **re6m6_13**<br>(8/77){-----X--}(4/78) **UMN441A**<br>(5/58){-----X--}(2/58) **UMN5828_2**<br>(3/77){--X-}(2/77) **opt-10269**<br>(3/77){--X-}(2/78) **opt-10269_c**<br>(1/75){-X-}(1/76) **opt-11790**<br>(2/75){-X-}(2/76) **opt-13288**<br>(2/72){-X-}(2/74) **opt-1424**<br>(2/73){-X-}(1/74) **opt-14369**<br>(5/77){---X}(0/78) **opt-15417**<br>(5/76){----X}(0/78) **(Dup?)(ML?)opt-16251**<br>(4/72){---X-}(1/73) **opt-17535**<br>(5/74){----X}(0/75) **opt-2428**<br>(8/67){-------X---}(4/67) **(Dup?)(K+0+)opt-6385_rp**<br>(6/70){-----X-}(2/70) **(Dup?)(K+0+)(ML?)opt-6421_rp**<br>(4/72){---X}(0/72) **opt-6852**<br>(5/78){---X}(0/79) **opt-6852_c**<br>(2/75){-X-}(2/76) **opt-7232**<br>(5/77){---X}(0/79) **opt-7371**

		(5/75){----X}(0/76) **opt-9239**<br>(5/77){---X}(0/78) **opt-9239_c**<br>(2/53){--X-}(1/53) **U8PM2**<br>(2/47){--X-}(1/48) **U8PM7**<br>(4/48){-----X-}(1/49) **U8PM8**<br>(8/70){------X----}(5/71) **AME067-2**<br>(7/68){------X---}(4/69) **AME016**
44	**(Dup?)(ML?)opt-16251_c**	(8/68) {-------} = 7 cM<br>(9/53){----------X--------------}(10/46) **e2m5_7x**<br>(11/49){---------------X----------}(7/41) **re4m8_12x**
51	**opt-4041**	(12/51) {----------------} = 16 cM
67	**ISU1254B**	(6/56) {-----} = 5 cM<br>(2/53){-X--------}(7/54) **CDO1357A**<br>(2/59){--X--------}(8/60) **CDO414**<br>(2/59){--X-------}(8/60) **CDO420A**<br>(4/59){---X----------}(10/60) **CDO545**<br>(0/59){X-----}(6/56) **ISU1247B**<br>(4/56){---X--------}(8/57) **ISU1736**<br>(10/59){-----------X------------------------}(19/60) **ISU2013**
72	**ISU1900A**	

Go to top of this map

Linkage Group: 19+25+27

Total cM: 73

Framework Map		Placed markers (informative recombination fraction is shown in brackets)
**cM**	**Marker**	
0	**opt-3655_c**	(5/80) {---} = 3 cM<br>(0/78){X---}(5/78) **opt-3655**<br>(5/78){---X}(0/78) **opt-5740**<br>(0/79){X---}(5/79) **opt-6993**<br>(4/79){---X}(0/79) **opt-8186**
3	**opt-5740_c**	(3/66) {--} = 2 cM<br>(4/61){----X}(0/58) **ISU1247A**<br>(4/61){----X}(0/58) **ISU1254A**

6	**CDO420B**	(7/65) {------} = 6 cM<br>(9/66){--------X-}(1/79) **opt-13747**<br>(9/66){--------X-}(1/79) **opt-13747_c**<br>(9/66){--------X-}(1/79) **opt-17272**
12	**opt-13460**	(19/74) {------------------} = 18 cM<br>(16/74){--------------X---------------}(16/71) **BCD1872A**<br>(16/76){--------------X------------}(14/73) **ACO193BRV**<br>(14/58){----------------X---------------------}(16/57) **(Seg)CDO942**<br>(9/58){---------X---------------}(13/57) **re6m4_18x**<br>(10/60){----------X---------------}(13/59) **Wacgcaa344**<br>(16/77){-------------X----------}(12/73) **opt-10485**<br>(16/77){-------------X----------}(12/75) **opt-15257**<br>(16/79){-------------X----------}(12/75) **opt-15257_c**<br>(17/75){---------------X-----------}(13/72) **opt-15904**<br>(15/74){-------------X-----------------}(17/70) **opt-17126_rp**<br>(14/73){------------X----------}(12/72) **opt-6137**
30	**opt-16182**	(4/75) {---} = 3 cM<br>(10/66){--------X---------}(10/68) **CDO1508A**<br>(11/61){-----------X------------}(12/62) **KSU_A1**<br>(7/58){-------X---------}(9/59) **re6m4_25ax**<br>(3/54){---X--}(2/55) **Waagcac236**<br>(12/55){--------------X-------------------}(15/56) **Wacacac1032**<br>(7/73){-----X--}(3/75) **opt-0028**<br>(6/75){----X-}(2/77) **opt-13973**<br>(4/75){---X}(0/77) **(Dup?)(ML?)opt-14172**<br>(6/72){-----X-}(2/74) **opt-14984**<br>(6/74){----X-}(2/76) **opt-14995**<br>(6/73){----X-}(2/75) **opt-15647**<br>(6/75){----X-}(2/77) **opt-16107**<br>(6/74){----X-}(2/76) **opt-16408**<br>(6/74){----X-}(2/76) **opt-16660**<br>(4/74){---X}(0/75) **(Dup?)(ML?)opt-17866**<br>(5/73){----X-}(2/75) **opt-2019**<br>(6/73){----X-}(2/75) **opt-2978**<br>(4/72){---X-}(2/74) **opt-4084**<br>(6/75){----X-}(2/77) **opt-4084_c**<br>(10/68){---------X--------------}(15/69) **(Dup?)(>65%)opt-4209_rp**<br>(5/74){----X-}(2/76) **opt-5897**

33	**(Dup?)(ML?)opt-17866_c**	(5/74) {----} = 4 cM<br>(8/61){-------X-------}(8/61) **CDO1402A**<br>(7/69){------X-}(1/69) **ACO182B**<br>(12/72){----------X---------}(11/71) **ACO182D**<br>(7/69){------X-}(1/69) **CDO358ARI**<br>(12/72){----------X---------}(11/71) **CDO358DDI**<br>(10/74){--------X-------}(9/73) **CDO58B**<br>(13/56){----------------X------------}(11/56) **e6m5_4ax**<br>(4/59){----X----}(4/59) **ISU582A**<br>(5/62){----X---}(3/62) **UMN339C**<br>(16/60){------------------X----------------}(14/60) **(Seg)UMN5249_1**<br>(3/57){---X---}(3/57) **Waagcac333**<br>(2/59){--X-}(1/59) **Wacacac172**<br>(2/59){--X----}(4/59) **Wacgcac790**<br>(8/71){------X--}(3/73) **(Dup?)(K+0+)opt-2785_rp**<br>(9/77){-------X-}(2/76) **opt-3999**<br>(3/73){--X-----}(7/73) **opt-5217**<br>(2/73){-X-----}(6/72) **opt-6961**<br>(8/71){------X----}(5/70) **opt-7231**<br>(7/74){-----X-}(2/73) **opt-9270**<br>(6/71){-----X--}(3/70) **(Dup?)(ML?)opt-5271_rp**
36	**opt-3600**	(19/73) {------------------} = 18 cM
55	**opt-6935**	(9/51) {-----------} = 11 cM
66	**BCD1235**	(6/51) {-------} = 7 cM<br>(3/53){---X--------}(8/59) **CDO412A**
73	**Waaccac273**	

Go to top of this map

Linkage Group: 21_46_31_40

Total cM: 125

Framework Map		Placed markers (informative recombination fraction is shown in brackets)
**cM**	**Marker**	
0	**Waaccac422**	(4/60) {----} = 4 cM<br>(5/60){-----X----}(5/63) **UMN51C**<br>(1/58){-X-----}(5/59) **Waaccac384**
4	**CDO541**	(20/75) {------------------} = 18 cM

22	**ACO190RV**	(5/59) {-----} = 5 cM<br>(9/73){-------X-}(2/57) **CDO419D**<br>(8/75){------X--}(2/59) **opt-11096**<br>(8/75){------X--}(2/59) **opt-11096_c**<br>(8/74){-------X--}(2/59) **opt-11219**<br>(8/75){------X--}(2/59) **opt-11779**<br>(8/74){-------X--}(2/59) **opt-12166**
27	**CDO1469BDI**	(3/58) {---} = 3 cM<br>(13/57){---------------X------------------}(15/58) **BCD1764**<br>(1/37){-X----}(3/38) **CDO586B**<br>(7/58){-------X--------}(8/58) **e6m4_22x**<br>(9/48){------------X----------}(8/48) **re4m8_10x**<br>(3/54){---X------}(8/70) **opt-0232**
29	**opt-17786**	(2/60) {-} = 1 cM<br>(5/59){-----X---}(4/60) **BCD782A**<br>(3/75){--X---}(4/61) **CDO1407A**<br>(11/59){------------X----------}(10/60) **e1m3_8x**<br>(3/42){----X----}(4/43) **e2m2_23ax**<br>(4/52){----X-----}(4/52) **e2m5_10x**<br>(5/53){-----X------}(6/53) **e2m5_5x**<br>(4/57){----X---}(4/58) **re1m3_27x**<br>(1/42){-X--}(2/43) **re2m2_23bx**<br>(5/60){-----X-----}(6/60) **re4m6_8x**<br>(4/47){-----X-----}(4/48) **re5m3_7x**<br>(5/74){----X-}(1/61) **UMN441B**<br>(0/70){X-}(2/56) **opt-7051**<br>(3/74){--X----}(4/60) **opt-9184**<br>(2/67){--X--}(2/53) **AME102**<br>(8/65){-------X-------}(6/51) **AME053**<br>(3/70){--X---}(4/55) **AME023c**
31	**BCD1250**	(4/58) {---} = 3 cM<br>(7/55){-------X-----}(5/52) **e1m6_18x**<br>(6/57){------X-}(1/67) **opt-16233**
34	**opt-4849**	(18/72) {-----------------} = 17 cM
51	**opt-18005**	(1/75) {-} = 1 cM
52	**opt-2772**	(12/76) {---------} = 9 cM

61	**opt-6863_c**	(4/79) {---} = 3 cM<br>(6/62){-----X--------}(8/63) **BCD1230B**<br>(4/62){---X------}(6/63) **BCD1897B**<br>(6/62){-----X----}(5/63) **CDO1246A**<br>(4/60){----X------}(6/61) **ACO127RI**<br>(4/78){---X}(0/79) **opt-0499**<br>(4/79){---X}(0/79) **opt-12727**<br>(4/78){---X}(0/79) **opt-14893**<br>(0/79){X---}(4/79) **opt-17220**<br>(0/77){X---}(4/77) **opt-17248**<br>(0/73){X--}(3/73) **(Dup?)(>65%)opt-2231**<br>(0/78){X---}(4/78) **opt-2707**<br>(0/76){X--}(3/76) **opt-3795**<br>(4/78){---X}(0/78) **opt-3930**<br>(0/78){X--}(3/78) **opt-6863**<br>(4/77){---X}(0/78) **opt-8555**<br>(4/76){---X}(0/77) **opt-9634**<br>(19/72){------------------X------------------}(19/73) **opt-9958**
64	**opt-12727_c**	(10/79) {-------} = 7 cM
71	**opt-0031_c**	(2/77) {-} = 1 cM<br>(10/62){----------X-----------}(11/62) **CDO189**<br>(5/61){----X---}(3/61) **ISU1958B**<br>(3/62){---X-}(1/62) **UMN442B**<br>(0/75){X-}(2/74) **opt-0031**<br>(1/77){-X--}(3/77) **opt-10313**<br>(0/77){X-}(2/76) **opt-10313_c**<br>(0/78){X-}(2/77) **opt-11056**<br>(3/78){--X---}(4/77) **opt-14530**<br>(0/78){X-}(1/76) **opt-5998**<br>(0/75){X-}(2/75) **opt-7664**
72	**CDO1312A**	(2/72) {--} = 2 cM<br>(4/76){--X}(0/73) **opt-16915_c**<br>(4/76){--X}(0/73) **opt-16939**<br>(4/74){--X-}(1/71) **opt-4211**
74	**opt-17877**	(9/52) {-----------} = 11 cM
85	**Wacccac263**	(1/52) {-} = 1 cM
86	**opt-9949**	(5/67) {----} = 4 cM
90	**opt-14755_c**	(4/68) {---} = 3 cM<br>(0/77){X----}(5/68) **opt-14755**<br>(0/75){X---}(4/66) **opt-4951**

93	**CDO1436A**	(8/59) {--------} = 8 cM<br>(10/59){-----------X---------}(8/55) **(Seg)e2m2_12**<br>(10/65){---------X--}(2/56) **opt-10488**<br>(10/69){---------X----}(4/59) **opt-15993**<br>(10/67){--------X----}(4/57) **opt-15993_c**<br>(8/66){-------X--}(2/57) **opt-17121**<br>(9/68){--------X--}(2/58) **opt-17121_c**<br>(11/64){-----------X-------}(6/54) **(Dup?)(>65%)opt-2056**
101	**UMN5254_2C**	(18/59) {------------------------} = 25 cM<br>(22/51){!!!X-------------}(11/54) **(Seg)Wacgcac695**<br>(18/59){------------------------X}(0/80) **(Dup?)(>65%)opt-12825**<br>(18/59){------------------------X}(0/79) **(Dup?)(>65%)opt-17026**<br>(18/59){------------------------X}(0/80) **(Dup?)(>65%)opt-5185**<br>(18/52){---------------------------X---------------}(16/70) **(Dup?)(>65%)(K+0+)opt-5497**<br>(16/55){-----------------------X-}(1/75) **(Dup?)(>65%)opt-6258**<br>(18/58){------------------------X}(0/79) **(Dup?)(>65%)opt-7136**<br>(18/59){------------------------X}(0/80) **(Dup?)(>65%)opt-7545**<br>(18/59){------------------------X}(0/80) **(Dup?)(>65%)opt-8901**
125	**(Dup?)(>65%)opt-7136_c**	

Go to top of this map

Linkage Group: 22_44_18

Total cM: 178

Framework Map		Placed markers (informative recombination fraction is shown in brackets)
**cM**	**Marker**	

0	**opt-9086_c**	(3/78) {--} = 2 cM<br>(5/62){----X-}(1/61) **ACO146**<br>(5/58){-----X--}(2/57) **UMN5327_3**<br>(7/53){--------X-----}(5/53) **UMN6003A**<br>(3/58){---X}(0/57) **Wacgcac300**<br>(9/79){------X----}(6/77) **opt-0075**<br>(9/80){------X----}(6/78) **opt-0075_c**<br>(0/78){X--}(3/76) **opt-10396**<br>(13/72){-----------X---------}(11/70) **(Dup?)(>65%)opt-11959**<br>(15/76){-------------X----------}(12/74) **(Dup?)(>65%)(K+0+)opt-12658_rp**<br>(19/77){-----------------X----------------}(18/75) **(Dup?)(>65%)opt-15408_c**<br>(4/80){---X-}(1/78) **opt-15814**<br>(9/79){------X----}(6/77) **opt-15874**<br>(3/77){--X}(0/75) **opt-16745**<br>(19/74){------------------X-----------------}(18/72) **(Dup?)(>65%)opt-17501**<br>(19/74){------------------X-----------------}(18/72) **(Dup?)(>65%)opt-17501_c**<br>(19/79){----------------X----------------}(18/77) **(Dup?)(>65%)opt-4292**<br>(3/79){--X}(0/77) **opt-6636**<br>(3/80){--X}(0/78) **opt-6636_c**<br>(4/79){---X-}(1/77) **opt-6949**<br>(9/79){------X----}(6/77) **opt-8819**<br>(19/77){-----------------X---------------}(17/75) **(Dup?)(>65%)opt-9019**<br>(0/79){X--}(3/77) **opt-9086**<br>(9/78){-------X----}(6/76) **opt-9440**<br>(11/63){-----------X-------}(8/62) **ubc352Os**
2	**CDO482B**	(18/59) {------------------------} = 24 cM<br>(19/71){-------------------X-----------------}(13/53) **(Seg)AME114**
26	**UMN5596_1**	(23/60) {------------------------------------} = 36 cM<br>(3/52){---X---------------------------------------}(28/70) **CDO482A**<br>(9/54){----------X---------------------------}(19/57) **ISU1372B**<br>(11/56){------------X--------------------------------}(21/59) **ISU1900B**<br>(6/50){--------X---------------------------------}(20/53) **UMN6003C**<br>(7/60){-------X!!!}(33/78) **opt-0898**<br>(8/59){--------X-----------------------------------}(29/78) **opt-16578**<br>(7/55){-------X!!!}(29/70) **opt-16709**

62	**UMN5004**	(6/61) {-----} = 5 cM<br>(2/61){--X----}(4/59) **CDO1469ADI**<br>(6/63){-----X------}(6/61) **CDO1523B**<br>(4/63){---X-----}(6/61) **CDO113**<br>(14/62){---------------X-----------------------}(18/60) **ISU2124A**<br>(10/58){-----------X------------}(11/56) **re5m7_7x**
67	**ISU1507**	(6/59) {------} = 6 cM
73	**opt-9724**	(2/74) {-} = 1 cM<br>(2/77){-X-}(2/75) **opt-16618**
74	**opt-13454**	(2/76) {-} = 1 cM<br>(4/74){---X---}(5/78) **BCD115B**<br>(1/51){-X}(0/54) **CDO419E**<br>(3/57){---X----}(4/60) **CDO608CRV**<br>(4/50){----X----}(4/52) **e2m3_5x**<br>(9/52){-----------X---------}(8/53) **(Seg)e4m8_3x**<br>(14/56){-----------------X----------------}(14/59) **(Seg)e5m7_4x**<br>(4/60){----X----}(5/63) **ISU1537**<br>(2/74){-X}(0/77) **opt-10101**<br>(2/74){-X}(0/78) **(Dup?)(>65%)opt-10632**<br>(2/73){-X}(0/77) **(Dup?)(>65%)opt-12991**<br>(2/69){-X-}(2/72) **(Dup?)(>65%)opt-14690**<br>(2/75){-X}(0/79) **opt-15397**<br>(1/74){-X}(0/78) **(Dup?)(>65%)opt-16165**<br>(2/74){-X}(0/78) **(Dup?)(>65%)opt-16431**<br>(2/74){-X}(0/78) **(Dup?)(>65%)opt-18281**<br>(2/73){-X}(0/77) **opt-3030**<br>(6/66){-----X-----}(6/70) **(Dup?)(>65%)(ML?)opt-3613_rp**<br>(2/73){-X}(0/77) **opt-4523**<br>(2/75){-X}(0/79) **opt-4523_c**<br>(2/74){-X}(0/78) **(Dup?)(>65%)opt-7417**<br>(14/74){------------X----------}(13/78) **(Dup?)(>65%)opt-8483**<br>(14/74){------------X----------}(13/78) **(Dup?)(>65%)opt-8483_c**<br>(8/68){-------X-----}(6/72) **(Seg)AME065**
76	**(Dup?)(>65%)opt-7417_c**	(5/79) {---} = 3 cM<br>(7/61){-------X----}(5/61) **BCD180**
79	**opt-8196**	(12/72) {----------} = 10 cM

89	**opt-18069**	(5/56) {-----} = 5 cM<br>(3/56){---X----}(4/61) **CDO1246B**<br>(5/67){----X---}(3/61) **CDO506B**<br>(5/57){-----X-----}(6/62) **CDO770B**<br>(7/57){-------X----}(5/62) **ISU1163**<br>(12/55){--------------X----------}(10/60) **(Seg)ISU1958A**<br>(2/57){--X--}(2/62) **UMN28A**<br>(6/67){-----X----}(4/57) **(Dup?)(>65%)opt-10092**<br>(2/71){-X----}(4/58) **opt-16706**<br>(3/62){---X------}(6/55) **(Dup?)(>65%)(K+0+)opt-1843_rp**
94	**CDO484A**	(10/62) {----------} = 10 cM<br>(4/60){----X-----}(6/67) **CDO1437B**<br>(4/62){----X------}(9/79) **CDO419**
104	**opt-1641_c**	(4/73) {---} = 3 cM<br>(7/58){-------X-----------}(9/52) **BCD1842B**<br>(5/61){----X--------}(8/56) **UMN5207A**<br>(5/79){---X-}(1/72) **opt-12704**<br>(5/79){---X-}(1/72) **opt-12704_c**<br>(4/78){---X-}(1/71) **opt-13230**<br>(8/78){------X-----}(7/71) **(Dup?)(ML?)opt-14317**<br>(0/78){X--}(3/71) **opt-15990**<br>(0/71){X--}(2/65) **opt-1641**<br>(8/76){------X----}(5/69) **opt-16678**<br>(8/78){------X----}(5/71) **opt-16885**<br>(0/76){X--}(3/69) **opt-17493**<br>(8/79){------X----}(5/72) **(Dup?)(ML?)opt-18190**<br>(8/79){------X-----}(6/72) **(Dup?)(ML?)opt-18190_c**<br>(7/76){-----X----}(5/70) **opt-18277**<br>(9/79){------X----}(5/72) **opt-8771**<br>(9/79){------X----}(5/72) **opt-8771_c**<br>(2/78){-X--}(3/71) **opt-9132**<br>(8/79){------X-----}(6/72) **AM102**<br>(9/73){-------X----------}(11/67) **ksum139**
107	**opt-17202**	(5/69) {----} = 4 cM
111	**opt-6646**	(1/72) {-} = 1 cM<br>(0/75){X-}(1/74) **opt-14144**
112	**opt-5664**	(3/74) {--} = 2 cM<br>(3/57){---X----}(4/61) **re6m4_4x**

114	**opt-0526**	(1/76) {} = 0 cM<br>(5/74){----X----}(5/72) **BCD1643C**<br>(1/59){-X-}(1/59) **CDO1505b**<br>(1/58){-X-}(1/56) **BCD1734**<br>(3/51){---X---}(3/49) **BCD1797A**<br>(1/67){-X-}(2/65) **BCD1968C**<br>(9/63){--------X---------}(9/61) **CDO542**<br>(10/63){----------X----------}(10/61) **ISU1877**<br>(6/57){------X------}(6/55) **re6m5_2x**<br>(0/61){X}(0/59) **UAZ177**<br>(3/60){---X---}(3/58) **Waaccac239**<br>(1/58){-X-}(1/56) **Wacacac300**<br>(5/75){----X----}(6/73) **opt-10013**<br>(1/77){-X}(1/75) **opt-12921**<br>(1/79){-X}(1/77) **opt-13431**<br>(1/78){-X}(1/76) **opt-1373**<br>(1/79){-X}(1/77) **opt-1373_c**<br>(4/74){---X---}(4/72) **opt-15048**<br>(5/79){---X----}(6/77) **opt-15048_c**<br>(0/75){X}(1/74) **(Dup?)(ML?)opt-16436**<br>(5/76){----X----}(6/74) **opt-16979**<br>(2/77){-X-}(2/75) **opt-17223**<br>(5/76){----X----}(5/75) **opt-2754**<br>(2/76){-X-}(2/74) **opt-4877**<br>(5/77){---X----}(6/75) **opt-5642**<br>(1/75){-X}(1/73) **opt-7238**<br>(5/77){---X----}(6/75) **opt-7774_c**
114	**ISU1961CRV**	(5/60) {-----} = 5 cM<br>(5/59){-----X--}(2/60) **CDO1471**<br>(4/61){----X}(1/60) **ACO165RV**<br>(6/56){------X---}(3/57) **e5m5_10x**<br>(8/56){--------X-------}(7/57) **re5m5_11x**<br>(5/57){-----X-}(1/58) **UMN401**<br>(6/74){----X-}(1/59) **opt-17658**<br>(6/65){-----X-}(2/53) **opt-7774**
119	**CDO708B**	(10/58) {----------} = 10 cM

128	**ACO168A**	(4/55) {----} = 4 cM<br>(6/59){-----X--}(2/57) **ACO142A**<br>(4/62){----X----}(4/59) **CDO669B**<br>(6/47){-------X-------}(6/47) **re8m3_19x**<br>(4/62){---X--}(2/59) **UMN114**<br>(8/59){-------X---}(4/59) **UMN589B**<br>(8/61){-------X--}(2/59) **opt-12320**<br>(8/62){-------X--}(2/59) **opt-12320_c**<br>(6/59){------X--}(2/57) **opt-14316**<br>(6/59){------X--}(2/56) **opt-3348**<br>(5/59){-----X-}(1/56) **opt-7424**<br>(6/61){------X--}(2/58) **opt-9160**<br>(6/52){-------X---}(3/49) **AME088**
133	**CDO608DRV**	(9/57) {---------} = 9 cM
142	**opt-15453**	(1/76) {-} = 1 cM<br>(8/61){--------X---------}(9/61) **UMN302**<br>(4/69){---X----}(5/68) **AME138**
143	**opt-8693**	(2/77) {-} = 1 cM<br>(11/75){---------X-------}(9/76) **UMN857**<br>(3/51){---X---}(3/53) **U8PM29**
144	**opt-5272**	(2/79) {-} = 1 cM<br>(4/38){------X------}(4/38) **CDO590C**<br>(6/77){----X---}(4/76) **CDO795B**<br>(9/61){---------X-------}(7/60) **e6m6_11x**<br>(2/59){--X}(1/58) **Wacacaa285**<br>(2/79){-X}(0/79) **opt-11426**<br>(2/79){-X}(0/79) **opt-11625**<br>(0/79){X-}(2/79) **opt-12339**
146	**opt-11625_c**	(4/79) {---} = 3 cM<br>(5/59){-----X----}(4/60) **UMN5485**<br>(6/59){------X-----}(5/60) **WG232**<br>(3/78){--X}(0/79) **opt-4939**<br>(5/72){----X-}(1/73) **opt-5064**<br>(4/79){---X}(0/80) **opt-9929**<br>(4/69){---X--}(3/69) **AME178**
148	**opt-9929_c**	(4/76) {---} = 3 cM
151	**opt-14108**	(5/75) {----} = 4 cM<br>(12/73){---------X--------}(10/75) **UMN363B**
155	**opt-6715**	(4/62) {---} = 3 cM

158	**CDO1196**	(10/63) {----------} = 10 cM<br>(10/63){----------X-}(2/63) **BCD1588**<br>(10/54){------------X------}(6/54) **e2m5_9x**<br>(12/59){------------X-----}(6/59) **e3m7_22ax**<br>(12/61){------------X---}(3/61) **UMN267**<br>(12/60){-------------X----}(4/60) **UMN287**<br>(12/62){-------------X----}(4/62) **UMN360A**<br>(9/59){---------X-}(1/59) **UMN575**<br>(10/60){----------X---}(4/60) **UMN856A**<br>(22/62){-------------------------------X-----------------}(16/62) **L7M4.9a**
167	**BCD327**	(10/55) {-----------} = 11 cM<br>(14/61){----------------X--------------------------}(17/53) **AM1**
178	**CSU25**	

Go to top of this map

Linkage Group: 24_26_34

Total cM: 93

**Framework Map**		**Placed markers (informative recombination fraction is shown in brackets)**
**cM**	**Marker**	
0	**UMN5245_3**	(6/57) {------} = 6 cM<br>(10/59){----------X----}(4/61) **BCD1555**<br>(11/56){------------X-----}(5/57) **AB02.410**<br>(12/56){--------------X-----}(7/73) **ACO118A**<br>(8/52){---------X---}(3/53) **estD**<br>(8/58){--------X----}(4/60) **ISU1463**<br>(7/58){-------X-----}(5/60) **ISU1874A**<br>(10/58){-----------X----}(4/59) **re1m6_10n**<br>(9/56){----------X---}(3/58) **re5m7_5x**<br>(10/58){-----------X------}(6/60) **UMN41**<br>(6/49){--------X-----}(4/51) **UMN6003B**<br>(6/57){------X-}(1/58) **Wacgcac261**<br>(8/57){--------X---}(4/73) **WG110A**<br>(10/57){-----------X---}(4/73) **opt-10033**<br>(6/57){------X-}(1/74) **opt-10980**<br>(6/56){------X}(0/72) **opt-13556**<br>(7/58){-------X-}(1/75) **opt-14769**<br>(15/59){-------------------X--------------}(16/76) **(Dup?)(>65%)(K+0+)(ML?)opt-15247_rp**

		(15/54){-------------------X--------------}(15/69) **(Dup?)(>65%)opt-15408**<br>(6/58){------X}(0/75) **opt-15673**<br>(6/59){------X}(0/75) **opt-15673_c**<br>(7/59){-------X-}(1/76) **opt-17610**<br>(7/59){-------X-}(1/76) **opt-17610_c**<br>(6/59){------X}(0/76) **opt-18117**<br>(7/58){-------X}(0/75) **opt-2747**
6	**opt-4800**	(16/76) {--------------} = 14 cM<br>(5/59){-----X---------}(9/61) **e6m4_2a**<br>(4/73){---X------------}(15/77) **opt-10543**
20	**opt-2130_c**	(4/78) {---} = 3 cM<br>(8/61){-------X----}(4/60) **Bglucanase**<br>(6/75){-----X----}(6/73) **ACO118B**<br>(3/74){--X---}(4/72) **opt-12952**<br>(5/79){---X-}(1/78) **opt-15072**<br>(5/79){---X-}(1/78) **opt-15072_c**<br>(0/76){X-}(2/74) **opt-15376**<br>(0/79){X---}(4/77) **opt-17989**<br>(0/77){X-}(2/75) **opt-2130**<br>(5/78){---X}(0/77) **(Dup?)(ML?)opt-8386**<br>(3/77){--X}(0/76) **opt-8434**
22	**(Dup?)(ML?)opt-8386_c**	(12/76) {---------} = 9 cM
		(6/72) {-----} = 5 cM<br>(6/60){-----X--}(2/57) **CDO1469CDI**<br>(3/62){---X-------}(7/59) **CDO1523A**<br>(4/60){----X---}(3/57) **CDO241**<br>(7/48){---------X-----}(4/45) **(Seg)CDO419B**<br>(6/61){------X--}(2/58) **CDO484B**<br>(5/47){------X--------------}(10/47) **e2m3_7x**<br>(4/60){----X---}(3/57) **UMN5207B**<br>(5/74){----X------}(8/71) **opt-0003**<br>(6/73){----X-------}(9/70) **opt-0220**<br>(7/73){-----X------}(8/70) **opt-0859**<br>(7/73){-----X---------}(11/70) **opt-11295**<br>(6/72){-----X--------}(9/69) **opt-1205**<br>(3/72){--X-------}(9/71) **opt-13087**<br>(5/73){----X------}(8/70) **opt-1357**<br>(4/75){---X-}(2/71) **opt-15383**<br>(4/75){---X-}(2/71) **opt-15383_c**<br>(2/76){-X------}(8/74) **opt-16191**

32	**opt-7581**	(2/76){-X------}(8/74) **opt-16191_c**<br>(6/72){-----X-------}(8/69) **opt-1660**<br>(1/75){-X-----}(7/72) **opt-18007**<br>(6/71){-----X--------}(9/69) **opt-1841**<br>(7/74){-----X--------}(10/71) **opt-1935**<br>(7/74){-----X--------}(10/71) **opt-1935_c**<br>(5/73){----X------}(8/70) **opt-2434**<br>(5/71){----X------}(7/68) **opt-2670**<br>(1/71){-X----}(5/69) **opt-3061**<br>(5/74){----X------}(8/71) **opt-3343**<br>(6/74){----X-------}(9/71) **opt-3558**<br>(5/73){----X------}(8/70) **opt-3731**<br>(6/73){----X-------}(9/70) **opt-3747**<br>(5/73){----X------}(8/70) **opt-4470**<br>(6/75){----X-------}(9/72) **opt-4593**<br>(8/77){------X---------}(11/75) **opt-4593_c**<br>(5/73){----X------}(8/70) **opt-5065**<br>(5/73){----X------}(8/70) **opt-5402**<br>(6/73){----X-------}(9/70) **opt-5694**<br>(5/70){----X------}(7/67) **opt-5816**<br>(6/74){----X-------}(9/71) **opt-5841**<br>(6/72){-----X-------}(8/69) **opt-6092**<br>(6/71){-----X-------}(8/68) **opt-6437**<br>(5/73){----X------}(7/70) **opt-7143**<br>(6/71){-----X--------}(9/68) **opt-7693**<br>(2/75){-X-----}(7/72) **(Dup?)(K+0+)opt-8210_rp**<br>(6/72){-----X-------}(8/69) **opt-8378**<br>(6/72){-----X-------}(8/69) **opt-9233**<br>(6/75){----X-------}(9/72) **opt-9466**<br>(4/74){---X-----}(7/72) **opt-9657**<br>(6/74){----X-------}(9/71) **opt-9688**<br>(7/58){-------X---}(3/55) **AM87**

36	**opt-18063**	(6/72) {-----} = 5 cM<br>(3/59){---X-----}(6/62) **CDO1437A**<br>(7/54){--------X---}(3/57) **CDO1505a**<br>(12/56){--------------X---------}(9/59) **BCD1797D**<br>(8/59){--------X---}(4/62) **BCD1968B**<br>(4/58){---X------}(6/61) **CDO1168A**<br>(10/59){-----------X--------}(8/62) **(Seg)BCD1103**<br>(4/63){----X------}(7/66) **CDO457B**<br>(7/43){----------X---------------}(10/45) **e8m3_12n**<br>(10/71){--------X---}(4/73) **ISU1961BRV**<br>(6/56){------X-----}(5/59) **(Seg)re1m3_13b**<br>(8/59){--------X----}(5/62) **UMN220**<br>(7/55){-------X-----}(5/58) **Wacacac1456**<br>(11/54){-------------X--------}(8/57) **(Seg)Wacacac312**<br>(8/70){------X---}(4/71) **(Dup?)(>65%)opt-10825**<br>(11/74){---------X----}(5/76) **opt-12736**<br>(7/72){-----X---}(4/74) **opt-14149**<br>(11/74){---------X---}(5/77) **opt-17023**<br>(8/73){------X---}(4/74) **opt-3270**<br>(1/71){-X----}(5/71) **opt-5851**<br>(5/75){----X---}(5/77) **opt-9519**<br>(7/65){------X------}(7/67) **AME152**
41	**opt-9567**	(13/75) {-----------} = 11 cM<br>(10/61){----------X----------------}(14/60) **(Seg)BCD1643A**<br>(10/71){--------X--------------}(15/71) **BCD808B**<br>(10/58){-----------X---------}(9/57) **AD04.810**<br>(9/58){---------X-------}(7/57) **UMN624**<br>(10/74){--------X------------}(14/75) **opt-4341**<br>(11/69){----------X--------------}(15/70) **RMS514**<br>(9/70){-------X----------}(12/71) **AME141**
52	**opt-12104**	(8/76) {------} = 6 cM<br>(2/61){--X------}(7/63) **CDO1326**<br>(5/60){-----X----------}(10/62) **ACO168B**<br>(2/61){--X------}(7/63) **CDO348A**<br>(3/76){--X--------}(10/76) **opt-8249**
57	**CDO590A**	(5/78) {---} = 3 cM<br>(3/61){---X----}(5/61) **UMN589A**<br>(4/77){---X-----}(7/78) **opt-13299**

61	**CDO795A**	(4/79) {---} = 3 cM<br>(2/61){--X----}(4/61) **e2m2_21x**<br>(3/69){--X-}(1/70) **(Dup?)(K+0+)opt-0795_rp**<br>(6/69){-----X-}(2/70) **opt-17901**
64	**opt-13764**	(7/79) {-----} = 5 cM
68	**opt-8018_c**	(2/76) {-} = 1 cM<br>(4/35){------X---}(2/33) **CDO1449A**<br>(13/62){--------------X---------}(9/60) **BCD897**<br>(6/60){------X-----}(5/58) **ACO139RV**<br>(10/58){-----------X-------}(7/57) **CDO353A**<br>(4/75){---X-}(1/73) **opt-10750**<br>(4/78){---X}(0/75) **opt-10750_c**<br>(2/76){-X}(0/76) **opt-12091**<br>(4/74){---X}(0/71) **opt-12472**<br>(2/75){-X}(0/75) **opt-13924**<br>(0/77){X-}(2/75) **opt-17213**<br>(2/76){-X}(0/77) **opt-5469**<br>(0/79){X-}(2/76) **opt-8018**<br>(2/75){-X}(0/75) **opt-9307**
70	**opt-13924_c**	(6/77) {----} = 4 cM<br>(8/60){--------X----}(4/61) **re2m2_12x**<br>(12/72){-----------X-----------}(14/74) **UMN363A**<br>(8/76){------X--}(3/78) **opt-10822**<br>(6/76){----X}(0/78) **opt-14251**<br>(8/74){------X--}(3/76) **opt-16967**<br>(9/76){-------X---}(4/78) **opt-1747**<br>(9/76){-------X---}(4/78) **opt-1747_c**<br>(8/74){------X--}(3/76) **opt-2398**<br>(8/76){------X---}(5/78) **opt-3936**<br>(7/77){-----X-}(1/79) **opt-7822**<br>(7/74){-----X--}(3/76) **opt-8015**<br>(6/76){----X}(0/78) **opt-8478**
74	**opt-14251_c**	(12/79) {---------} = 9 cM<br>(14/77){-----------X---------}(12/78) **CDO373**<br>(14/77){-----------X---------}(12/78) **CDO758**<br>(9/59){---------X---------------}(13/59) **Wacacaa378**<br>(16/79){-------------X---}(5/80) **opt-0542_c_rp**<br>(16/78){-------------X---}(5/79) **opt-0542_rp**<br>(16/79){-------------X---}(5/80) **opt-11018_rp**<br>(9/76){-------X-------}(9/77) **opt-3026**

83	**opt-5986**	(10/63) {----------} = 10 cM<br>(9/63){--------X-}(1/63) **BCD1413**<br>(9/60){---------X--}(2/60) **BCD1965**<br>(12/60){-------------X-------------}(12/60) **ACO183ARV**<br>(7/75){-----X----}(4/60) **CDO419F**<br>(16/62){------------------X----------}(10/62) **ISU1651**<br>(11/62){-----------X------}(7/62) **ISU563B**<br>(16/75){--------------X----}(5/61) **PTA71B**<br>(8/56){--------X--------}(8/56) **re1m6_17x**<br>(14/61){---------------X--------------}(13/61) **(Seg)re2m2_4**<br>(6/60){------X----}(4/60) **re4m2_1x**<br>(14/58){----------------X-------}(7/58) **UMN5393**<br>(8/78){------X---}(3/62) **opt-0638**<br>(7/72){-----X----------}(9/55) **opt-0865**<br>(8/76){------X---}(4/62) **opt-4191**<br>(6/77){----X---}(3/61) **opt-8899**<br>(7/77){-----X---}(3/61) **opt-8899_c**<br>(8/62){-------X-----}(6/62) **ubc221As**<br>(6/62){-----X---}(4/62) **L7M4.9b**<br>(7/62){------X------}(7/62) **II2.17**
93	**BCD961**	

Go to top of this map

Linkage Group: 29_43

Total cM: 46

Framework Map		Placed markers (informative recombination fraction is shown in brackets)
**cM**	**Marker**	
0	**UMN360BRV**	(1/49) {-} = 1 cM<br>(2/56){--X--}(2/51) **re3m7_18x**<br>(2/51){--X--}(2/60) **AME041**
1	**UMN856B**	(12/65) {------------} = 12 cM
13	**CDO58A**	(16/51) {-------------------------} = 25 cM
38	**CDO1338**	(3/49) {---} = 3 cM<br>(9/47){------------X-------------}(13/65) **AME007**

41	**opt-18157**	(6/71) {-----} = 5 cM<br>(3/70){--X------}(8/72) **CDO677**<br>(5/73){----X-}(1/74) **opt-11175**<br>(2/69){-X----}(5/71) **opt-14552**<br>(5/74){----X-}(1/76) **opt-4166**<br>(5/74){----X-}(1/76) **opt-4166_c**<br>(0/72){X----}(5/74) **opt-4497**
46	**opt-2826**	

Go to top of this map

Linkage Group: 32

Total cM: 40

Framework Map		Placed markers (informative recombination fraction is shown in brackets)
**cM**	**Marker**	
0	**re6m6_2cx**	(5/59) {-----} = 5 cM<br>(14/60){----------------X---------------------}(17/60) **(Seg)BCD1414B**<br>(15/61){-----------------X--------------------}(17/61) **(Seg)BCD1840B**
5	**CDO395A**	(7/58) {-------} = 7 cM<br>(10/57){-----------X------------------}(14/55) **(Seg)e4m2_9x**<br>(2/60){--X-----}(5/58) **Waaccac164**<br>(3/60){---X----}(4/58) **Waaccac214**
12	**BCD1632A**	(6/59) {------} = 6 cM<br>(0/58){X-----}(7/71) **CDO203B**<br>(0/58){X-----}(7/71) **CDO1090B**<br>(0/58){X-----}(7/71) **CDO57A**
18	**opt-14395**	(4/61) {----} = 4 cM<br>(1/79){-X-----}(6/62) **opt-15222**<br>(2/76){-X----}(4/60) **opt-6324**
22	**ACO181**	(4/59) {---} = 3 cM<br>(4/55){---X}(1/57) **ACO137DI**<br>(4/53){----X---}(4/54) **AME012**

25	**BCD1338A**	(10/63) {----------} = 10 cM<br>(6/44){---------X-----}(4/44) **e6m5_17x**<br>(7/62){------X-----}(7/78) **opt-1661**<br>(7/62){------X-----}(7/78) **opt-1661_c**<br>(8/62){-------X------}(8/78) **opt-17430**<br>(7/63){------X-----}(7/79) **opt-8886**<br>(8/61){--------X------}(8/77) **opt-9956**<br>(8/61){--------X------}(8/77) **opt-9956_c**
34	**opt-10190_c**	(5/78) {---} = 3 cM<br>(6/75){----X--------}(10/75) **BCD1871**<br>(5/60){-----X--------}(8/59) **ACO183BRV**<br>(4/62){---X-------}(7/61) **CDO517**<br>(5/51){-----X-----------}(9/51) **e5m3_1x**<br>(7/62){------X-----------}(11/61) **e6m4_27x**<br>(0/79){X---}(5/79) **opt-0918**<br>(0/78){X---}(5/78) **opt-10190**<br>(1/77){-X---}(5/77) **opt-10325**<br>(6/78){----X-}(1/77) **opt-11795**<br>(0/79){X---}(5/78) **opt-14832**<br>(5/77){---X}(0/77) **opt-15377**<br>(5/78){---X}(0/77) **opt-3337**<br>(1/73){-X---}(4/73) **opt-6750**<br>(6/79){----X}(0/79) **opt-7716**<br>(16/74){--------------X----------}(12/74) **opt-8113**<br>(2/79){-X----}(6/79) **opt-8744**
38	**opt-3337_c**	(2/54) {--} = 2 cM<br>(4/78){---X--}(2/55) **UMN207A**
40	**SAD1**	

Go to top of this map

Linkage Group: 33

Total cM: 33

Framework Map		Placed markers (informative recombination fraction is shown in brackets)
**cM**	**Marker**	
0	**BCD269**	(5/55) {-----} = 5 cM<br>(12/50){----------------X----------------}(13/56) **(Seg)re3m7_7**<br>(10/53){------------X-----------------}(18/73) **UMN202**

5	**opt-7387**	(2/72) {-} = 1 cM<br>(8/62){-------X------}(6/60) **CDO1428B**<br>(7/62){------X------}(6/60) **BCD421B**<br>(13/62){--------------X----------------}(14/60) **CDO1321B**<br>(2/39){---X----}(3/38) **ACO227DI**<br>(17/75){---------------X-----------------}(18/73) **(Seg)CDO772**<br>(2/76){-X-}(2/74) **opt-11001**<br>(2/77){-X-}(2/75) **opt-11001_c**<br>(2/74){-X-}(2/74) **opt-13141**<br>(1/76){-X-}(1/75) **opt-1427**<br>(2/76){-X-}(2/74) **opt-14507**<br>(18/74){-----------------X-----------------}(18/72) **(Dup?)(>65%)opt-14599**<br>(18/74){-----------------X-----------------}(18/72) **(Dup?)(>65%)opt-14599_c**<br>(3/75){--X--}(3/73) **opt-14960**<br>(4/71){---X--}(2/68) **opt-16786**<br>(2/76){-X-}(2/75) **opt-16849**<br>(2/76){-X-}(2/75) **opt-3015**<br>(2/77){-X-}(2/75) **opt-3782**<br>(2/77){-X-}(2/75) **opt-3782_c**<br>(18/72){-----------------X------------------}(18/71) **(Dup?)(>65%)opt-6787**<br>(0/74){X-}(2/71) **opt-7966**<br>(2/74){-X-}(1/74) **opt-8987**<br>(2/76){-X-}(2/75) **opt-8987_c**<br>(1/76){-X-}(1/74) **opt-9873**<br>(3/69){--X----}(5/67) **AME013**<br>(6/70){-----X-------}(8/68) **(Seg)AME117**
6	**opt-2121**	(6/72) {-----} = 5 cM
11	**opt-11714**	(3/77) {--} = 2 cM<br>(1/54){-X--}(2/56) **BCD1414A**<br>(1/61){X-}(2/63) **BCD1796**<br>(1/61){-X--}(2/63) **BCD1840A**<br>(1/61){-X--}(2/63) **CDO1091**<br>(1/61){-X--}(2/63) **CDO1255**<br>(1/60){-X--}(2/62) **CDO527**<br>(2/77){-X--}(3/79) **opt-10919**

13	**opt-14516**	(1/78) {-} = 1 cM<br>(1/68){-X-}(1/67) **BCD880B**<br>(3/61){---X--}(2/60) **CDO836BRV**<br>(2/58){--X-}(1/57) **e8m3_13x**<br>(11/74){---------X-------}(9/72) **opt-2430_rp**<br>(11/72){---------X-------}(9/70) **AME055**
14	**opt-13366**	(20/74) {-------------------} = 19 cM
33	**(Dup?)(>65%)opt-3486**	

Go to top of this map

Linkage Group: 36<br><br>Total cM: 32		
Framework Map		Placed markers<br>(informative recombination fraction is shown in brackets)
cM	Marker	
0	**opt-1813**	(4/73) {---} = 3 cM<br>(9/51){-----------X-----------}(9/51) **re1m2_1ax**
3	**CDO1380**	(7/62) {------} = 6 cM
9	**BCD1716A**	(6/63) {-----} = 5 cM<br>(2/61){-X------}(6/61) **ACO245A**<br>(7/60){-------X-------------}(12/60) **e6m6_8**<br>(4/63){---X------}(7/63) **UMN23**
14	**CDO1321A**	(4/61) {----} = 4 cM<br>(4/50){----X------}(6/49) **re5m3_3**
18	**CDO534DI**	(2/58) {-} = 1 cM<br>(2/59){--X---}(4/64) **BCD1882B**<br>(2/51){--X--}(2/50) **e1m6_23x**<br>(12/61){------------X-------------}(12/60) **e6m4_1a**<br>(1/50){-X}(0/50) **re2m3_3x**<br>(14/60){-----------------X----------------}(14/59) **re6m4_2**<br>(2/55){--X---}(3/55) **re6m5_3a**<br>(2/55){-X--}(3/71) **UMN498A**<br>(6/60){-----X----}(6/75) **opt-0988**<br>(6/60){-----X----}(6/75) **opt-0988_c**<br>(6/56){-------X------}(8/74) **opt-17948**<br>(6/56){-------X------}(8/74) **opt-17948_c**<br>(2/57){-X}(0/74) **opt-4119**

		(4/59){----X----}(6/75) **opt-4995**
20	**(Dup?)(ML?)opt-1097**	(6/75) {----} = 4 cM<br>(15/70){--------------X------}(8/70) **BCD1407**<br>(7/58){-------X----}(4/60) **e1m3_6an**<br>(13/58){---------------X----------}(10/59) **re6m4_1c**<br>(0/74){X----}(6/75) **opt-0233**<br>(6/72){-----X}(0/74) **opt-11294**<br>(6/74){----X}(0/77) **opt-12382**<br>(7/72){-----X-}(1/71) **AME205**<br>(5/72){----X-----}(6/71) **AME003**<br>(6/77){-----X--------}(10/78) **AM3**
24	**opt-12382_c**	(9/66) {--------} = 8 cM
32	**CDO669A**	

Go to top of this map

Linkage Group: 37

Total cM: 13

Framework Map		Placed markers (informative recombination fraction is shown in brackets)
**cM**	**Marker**	
0	**BCD1829B**	(7/61) {-------} = 7 cM<br>(7/59){-------X-----}(5/57) **CDO1414B**<br>(9/50){-----------X-----------}(9/49) **re5m3_4x**<br>(12/62){------------X----------}(10/60) **opt-11524**<br>(8/62){-------X------}(6/60) **opt-18396**<br>(7/63){------X----}(5/61) **opt-9004**<br>(11/62){-----------X---------}(9/60) **S3S1.24b**<br>(7/52){--------X------}(5/50) **AME019a**
7	**UMN5032_1C**	(7/61) {-------} = 7 cM<br>(9/61){---------X----}(6/74) **opt-10253**<br>(10/61){----------X----}(6/74) **(Dup?)(ML?)opt-15994**<br>(7/60){-------X----------}(12/73) **opt-8654**
13	**UMN51B**	

Go to top of this map

Linkage Group: 39

Total cM: 1

Framework Map		Placed markers (informative recombination fraction is shown in brackets)
**cM**	**Marker**	
0	**CDO1516DI**	(2/68) {-} = 1 cM<br>(2/38){--X------}(4/40) **CDO1437C**
1	**CDO586**	

Go to top of this map

Linkage Group: 42

Total cM: 10

Framework Map		Placed markers (informative recombination fraction is shown in brackets)
**cM**	**Marker**	
0	**opt-11824**	(2/74) {-} = 1 cM<br>(4/74){---X--}(4/76) **ISU707A**<br>(4/72){---X--}(3/74) **UMN5084A**<br>(1/72){-X--}(3/74) **opt-2148**<br>(3/75){--X-}(1/77) **opt-6446**<br>(2/69){-X}(0/71) **opt-7795**<br>(3/75){--X-}(1/77) **opt-7795_c**
1	**opt-16471**	(2/58) {--} = 2 cM
3	**ACO227**	(2/57) {--} = 2 cM
5	**HKT1c**	(4/58) {----} = 4 cM<br>(6/61){-----X---}(4/77) **opt-10409**<br>(5/60){-----X---}(4/76) **opt-10409_c**<br>(4/56){----X---}(4/72) **opt-2325**<br>(4/60){----X--}(3/76) **(Dup?)(ML?)opt-7123**<br>(4/54){-----X--}(2/66) **AME023a**
10	**opt-18119**	

Go to top of this map

Linkage Group: 46

Total cM: 32

**Framework Map**		**Placed markers<br>(informative recombination fraction is shown in brackets)**
**cM**	**Marker**	
0	**CDO1433**	(16/62) {-------------------} = 19 cM
19	**opt-7489**	(12/59) {-------------} = 13 cM<br>(8/71){------X------------}(10/54) **AME186**<br>(5/68){----X---------}(8/51) **AME168**<br>(5/69){----X---------}(8/51) **AME038**
32	**AB09.510**	

Go to top of this map

Linkage Group: 47

Total cM: 8

**Framework Map**		**Placed markers<br>(informative recombination fraction is shown in brackets)**
**cM**	**Marker**	
0	**opt-17731_c**	(11/80) {--------} = 8 cM<br>(0/77){X--------}(11/77) **opt-11559**<br>(10/71){--------X}(0/71) **opt-13105**<br>(8/71){------X-}(1/71) **opt-13765**<br>(20/74){------------------X----------------------------------}(28/74) **opt-14187**<br>(21/77){-------------------X----------------------------------}(29/77) **opt-14187_c**<br>(0/79){X--------}(11/79) **opt-17731**<br>(8/67){-------X-}(1/67) **opt-18055**<br>(11/73){---------X---}(4/73) **opt-2931**
8	**opt-13105_c**	

Go to top of this map

Linkage Group: 48

Total cM: 13

**Framework Map**	**Placed markers<br>(informative recombination fraction is shown in**

cM	Marker	brackets)
0	**opt-16112_c**	(12/59) {-------------} = 13 cM<br>(0/76){X-------------}(12/58) **opt-1385**<br>(0/76){X-------------}(12/58) **opt-16112**<br>(0/75){X--------------}(12/57) **opt-3694**<br>(0/76){X-------------}(12/58) **opt-7997**
13	**Waaccac246**	

Go to top of this map

Linkage Group: 50

Total cM: 3

Framework Map		Placed markers<br>(informative recombination fraction is shown in brackets)
**cM**	**Marker**	
0	**opt-2274_c**	(4/70) {---} = 3 cM<br>(13/57){---------------X------------}(10/54) **e2m2_13**<br>(9/58){---------X-----}(5/55) **UMN5254_2B**<br>(0/75){X----}(5/69) **opt-17262**<br>(0/77){X----}(5/70) **opt-2274**
3	**CDO405**	

Go to top of this map

Linkage Group: 51

Total cM: 8

Framework Map		Placed markers<br>(informative recombination fraction is shown in brackets)
**cM**	**Marker**	
0	**AC06.600**	(8/60) {--------} = 8 cM
8	**ac06.625**	

Go to top of this map

Linkage Group: 52<br><br>Total cM: 6		
Framework Map		Placed markers<br>(informative recombination fraction is shown in brackets)
cM	Marker	
0	**Wactcac220**	(5/50) {------} = 6 cM<br>(7/46){---------X------------------}(11/43) **U8PM5**
6	**(Seg)Wactcac221**	

Go to top of this map

UnPlaced Markers:

- **BCD1280C**
- **CDO189RV**
- **BCD372A**
- **BCD372B**
- **CDO1092A**
- **(Seg)CDO1358B**
- **AA03.875**
- **aa12.380**
- **AC06.1475**
- **(Seg)AM18**
- **AM41**
- **(Seg)CDO419A**
- **COLEO**
- **(Seg)CSU154a**
- **CSU36b**
- **(Seg)CSU40a**
- **LOX11**
- **pABG20**
- **re4m8_15x**
- **(Seg)re5m7_4**
- **UAZ213a**
- **UMC44**
- **UMN110**
- **(Seg)UMN147**
- **UMN159**

- **(Seg)UMN5301_3A**
- **UMN5301_3B**
- **UMN5425_1A**
- **UMN5425_1B**
- **UMN5560A**
- **UMN5924**
- **(Seg)Wacacac867**
- **Wactcac1258**
- **Wactcac338**
- **Wactcac354**
- **opt-15182**
- **opt-15182_rp**
- **opt-17139**
- **(Dup?)(K+0+)opt-2420**
- **(Dup?)(K+0+)opt-2420_rp**
- **(Dup?)(K+0+)opt-8349**
- **(Dup?)(K+0+)opt-8349_rp**
- **(Dup?)(>65%)opt-8840**
- **opt-9983**
- **(Seg)ALrk1A1_A**
- **ubc352KS**
- **U71PM4**
- **(Seg)U8PM6**
- **(Seg)AME086**
- **(Seg)AME074**
- **AME197**

Project: Itamar
Home Page: http://res.agr.ca/prc/quaker/index.htm
Email: tinkerna@agr.gc.ca
Generated by: Multiple Molecular Marker Map Manager (M5) Version: Sep-28-2004
Creation date: 12/9/2008
